# Physical activity trajectories and accumulation over adulthood and their associations with all-cause and cause-specific mortality: a systematic review and meta-analysis

**DOI:** 10.1136/bjsports-2024-109122

**Published:** 2025-07-10

**Authors:** Ruyi Yu, Stephanie L Duncombe, Yuta Nemoto, Raphael HO Araujo, Hsin-Fang Chung, Gregore I Mielke

**Affiliations:** 1 School of Public Health, The University of Queensland, Brisbane, Queensland, Australia; 2 Department of Preventive Medicine and Public Health, Tokyo Medical University, Shinjuku-ku, Japan; 3 School of Health Innovation, Kanagawa University of Human Services, Kanagawa, Japan; 4 Londrina State University, Londrina, Brazil; 5 Health Research and Innovation Science Centre, Faculty of Health Sciences, Klaipeda University, Klaipeda, Lithuania

**Keywords:** Epidemiology, Health, Physical activity, Death, Cardiovascular Diseases

## Abstract

**Objective:**

To understand the associations of trajectories and accumulation of physical activity (PA) over adulthood with all-cause, cardiovascular disease (CVD), and cancer mortality.

**Design:**

Systematic review and meta-analyses.

**Data sources:**

PubMed, Embase, Scopus, Cochrane Library, Web of Science, CINAHL, MEDLINE, SPORTDiscus, and reference lists of included studies.

**Eligibility criteria:**

Population-based prospective studies with (1) non-clinical adult population, (2) PA assessed ≥2 timepoints as exposure, (3) all-cause, CVD or cancer mortality as outcomes with risk measures, and (4) publication up to 9 April 2024 and in English.

**Results:**

85 studies with three main types of PA exposure (ie, trajectory, time-varying, and cumulative/average) were included. Of these, 77 assessed all-cause mortality, 34 assessed CVD mortality, and 15 assessed cancer mortality. Overall, higher PA was associated with lower risks of all outcomes. Consistently and increasingly active individuals had around 20–40% lower risk of all-cause mortality and 30-40% lower risk of CVD mortality; however, the associations with decreasing PA patterns were less evident. Time-varying and cumulative/average PA illustrated similar inverse associations between higher PA levels and all-cause and CVD mortality. The associations were weaker and less robust for cancer mortality. Non-linear dose-response associations suggested risk reductions in all-cause and CVD mortality for meeting PA guidelines, but consistent/increasing PA below the guidelines also provided health benefits.

**Conclusions:**

Consistently/increasingly accumulated PA over adulthood can reduce the risk of all-cause and CVD mortality, while the health benefits from decreasing PA patterns require further exploration.

**WHAT IS ALREADY KNOWN ON THIS TOPIC**

- Higher physical activity (PA) levels are generally associated with a lower risk of all-cause and cause-specific mortality.
- Meeting PA guidelines is generally recommended to reduce adverse health outcomes.
- PA level is not constant across the life course, but most studies examining the associations between PA and health outcomes only measured PA at a single timepoint.

**WHAT THIS STUDY ADDS**

- This review provides the first and currently largest meta-analysis of quantitative estimates of risk reduction for different mortality outcomes based on different PA patterns.
- Repeated PA measurements enable more diverse approaches to model PA accumulation over time and allow for more accurate effects estimates for health outcomes compared with single PA measurements.
- Being consistently active is associated with around 30–40% risk reduction in all-cause mortality, while increasing PA is associated with 20–25% lower risk of all-cause mortality.
- Binary categorisations of PA possibly conceal the benefits of decreasing PA from high to low, whereas using ≥3 PA categories may provide greater insight for future research.
- In consistent and increasing PA patterns, the most substantial risk reduction occurred below the PA guidelines, while limited additional benefits were seen above the guidelines, particularly in increasing patterns.

**HOW THIS STUDY MIGHT AFFECT RESEARCH, PRACTICE OR POLICY**

- Initiating PA at any point in adulthood can provide health benefits. In the meantime, future PA interventions may not only target inactive people, but also support active people to maintain their activity.

## Introduction

 Physical inactivity is associated with a range of non-communicable diseases and is the fourth leading risk factor for mortality globally.^1^ Currently, the WHO recommends that adults should aim for 150 to 300 min per week of moderate-intensity physical activity (PA), or 75 to 150 min per week of vigorous-intensity activity, or a combination of the two.^2^ These recommendations were based on the best evidence available, making use of up-to-date systematic reviews from a wide range of databases.^2^ However, the current evidence predominantly includes prospective studies with a one-time-point assessment of PA,^3^ which might hide the changing patterns of PA during adulthood. A previous study examining the stability of PA habits suggests that almost half of the adult population changed their PA levels over a course of a decade, which can affect the interpretation of the long-term health benefits of PA when only measured once.^4^ There is also evidence suggesting that repeated measurements of PA may better predict adverse health outcomes than a single measurement of recent PA.^5^ In addition, as a range of PA patterns was identified in adult populations,^3 6^ it has been suggested that different PA patterns might be associated with different health outcomes.^7^ Therefore, the Guideline Development Group emphasised the importance of more research identifying different PA patterns over time from various intensities and domains like household, work and leisure time.^8^

Several reviews have examined the longitudinal association between PA and health outcomes, including the incidence of chronic diseases,^9^ mental health,^10^ and all-cause and cause-specific mortality.^11^,^13^ However, these reviews did not restrict the number of PA assessments in their inclusion criteria (ie, most findings were based on a single measurement of PA). This might result in misclassification due to the dynamic nature of PA^14^ and limit the ability to assess the changing patterns of PA. Some reviews have explored general changes in PA over a certain period or across the life course,^15^,^17^ and how different factors relate to the changes.^16^ However, there were limited studies on the associations between changes in PA across adulthood and health outcomes in older ages.

A previous systematic review that summarised 57 studies (up to January 2021) examining PA across adulthood and the mortality risks from all-cause, cardiovascular disease (CVD) and cancer in later life^3^ suggested that being consistently or increasingly active over adulthood was associated with a lower risk of all-cause and CVD mortality compared with being consistently inactive, while only five studies examined the associations between changes in PA with cancer mortality and showed inconclusive results. Several methods were identified to model repeated PA assessments, including trajectories, time-varying exposures, and cumulative/average PA volumes. Due to the high level of heterogeneity in PA assessments/categorisation, exposure modelling and statistical analyses, the authors did not perform meta-analyses. Although the authors presented the risk ratios using forest plots, the incomparability between PA categories across studies remained a concern. Since this systematic review, more studies were published in the following years, which might help overcome the limitations the authors observed. Additionally, although it has been suggested that PA levels above the recommended guidelines might give additional health benefits, the evidence base was limited.^2^ Understanding the dose-response relationships between different PA patterns across adulthood and mortality risks would be beneficial for developing public health strategies aiming at increasing population PA levels.

Therefore, the aim of this study was to conduct a systematic review with meta-analyses to understand better the associations of trajectories and accumulation of PA over adulthood with all-cause, CVD and cancer mortality. To overcome the challenges posed by different analytical methods, the analyses were performed separately for each type of PA exposure modelling (ie, trajectories, time-varying, and cumulative/average). The secondary aim was to explore the dose-response gradients between the volumes of accumulated PA over adulthood and mortality risk for different types of PA exposure modelling.

## Methods

The study protocol was registered with the PROSPERO database (Registration ID: CRD42023452686).

### Eligibility criteria

Studies were eligible for inclusion if: (1) participants were at least 18 years old at recruitment and were not selected based on clinical conditions or pre-existing diseases; (2) studies were population-based, prospective, and observational; (3) the exposures of interest were physical activity (of any type) assessed at two or more time points; (4) the outcomes of interest were all-cause, CVD, or cancer mortality, with reported effect estimates as hazard ratios (HR), odds ratios (OR) or relative risks (RR) with 95% confidence intervals (95% CI); and (5) studies were peer reviewed, published up to 9 April 2024 and in English. Studies were excluded if they: (1) measured sedentary behaviours only; (2) measured fitness only (eg, cardiorespiratory fitness, physical fitness, or exercise capacity); (3) combined physical activity with other risk factors; (4) included convenience sampling method; and (5) were systematic reviews or meta-analyses.

### Search strategy

We performed a systematic search in eight databases (PubMed, Embase, Scopus, Cochrane Library, Web of Science, CINAHL, MEDLINE and SPORTDiscus) to retrieve relevant studies up to 9 April 2024. Keywords relevant to ‘physical activity’, ‘mortality’, ‘longitudinal’, and ‘prospective study’ were adopted for literature retrieval. The search terms are listed in online supplemental tables 1A and 1B. Boolean logic was used to group the keywords: ‘AND’ was used among the groups of keywords, and ‘OR’ was used within each group. After removing duplicates, four reviewers (RY, GM, YN, SD) screened the search results independently to exclude irrelevant studies based on the title. Then, six reviewers (RY, GM, YN, HC, SD, RA) independently reviewed the abstract and full text of the papers in detail to assess eligibility. The entire screening and selection process was performed in Covidence. Each article was assessed by two reviewers for inclusion/exclusion decisions and the reason for exclusion. Studies with conflicts were listed separately, which were resolved via discussion between reviewers. Reference lists of the included studies and relevant systematic reviews were also screened for additional relevant studies.

### Data extraction

Data were extracted by one author and checked for consistency by a second author in 10% of the cases. Discrepancies were checked against the full text and resolved by discussion with co-authors. The data extracted from each study included first author, publication year, study design, study country, time points of assessments, years of follow-up, participant characteristics (gender and age), assessment of physical activity (methods, type, measurements, and categories), mortality ascertainment (methods and cause of death), statistical model, covariates in final models, and the effect estimates for mortality risk (HR, OR, or RR). As we assumed HRs to be the primary effect estimates for our meta-analyses, relevant authors were contacted for HR estimates if other risk measures were reported. When this approach was not feasible, RRs were used as summary estimates in the meta-analyses, with random-effects models used to account for different risk measures.^18^ In the meta-analyses, results stratified by gender or other attributes (eg, age and ethnicity) were included as separate associations. When there were multiple sport types in a single study, we selected the most prevalent one in the cohort. When results were stratified by activity intensities, only risk estimates for moderate/vigorous intensity were extracted for meta-analyses. For studies that assessed multiple domains of PA (eg, household, occupational, transport, and leisure-time), we only extracted the risk estimates for total and/or leisure-time PA, which are most frequently reported across studies. Effect estimates were extracted from the fully adjusted model. For studies using data from the same cohort, we accounted for double counting of participants in analyses by choosing one study from each cohort if they had overlapping follow-up periods and measured the same outcomes. Generally, studies were selected independently for each outcome, based on more recent data, longer length of follow-up and larger sample sizes. When studies from the same cohort had no overlapping follow-up periods or measured different outcomes, their results were retained for analyses. For studies using pooled results from multiple cohorts, we extracted the risk estimates for individual cohorts if they were reported; otherwise, we excluded the studies from meta-analyses.

### Risk of bias assessment

Two authors independently assessed the risk of bias using the Newcastle-Ottawa quality assessment scale (NOS) for cohort studies. The results of the assessments were mostly aligned. Discrepancies were double-checked against the full text and resolved by discussion. Nine items were used to assess the quality across three major domains (ie, selection, comparability, and outcome).^19^ The selection domain contains four items, comparability contains two items, and outcome contains three items. Details of the risk assessment scale are shown in online supplemental table 2A.

According to the Agency for Healthcare Research and Quality (AHRQ) standards of NOS, the main domains of the risk of bias assessment were classified as good quality (three or four stars in selection domain AND one or two stars in comparability domain AND two or three stars in outcome domain), fair quality (two stars in selection domain AND one or two stars in comparability domain AND two or three stars in outcome domain), or poor quality (zero or one star in selection domain OR zero stars in comparability domain OR zero or one star in outcome domain).

We used a directed acyclic graph to determine the confounding factors of the association between long-term PA over adulthood and mortality risks (online supplemental figure 1), which included sociodemographic factors, lifestyle factors, baseline health status or pre-existing chronic diseases, and adiposity indicators. This contributed to the risk of bias assessment for the sufficiency of covariates adjustment.

### Equity, diversity and inclusion

This review included all eligible peer-reviewed studies irrespective of gender, race or ethnicity of participants. The co-author team comprised 50% females and a broad range of ethnic, racial and socio-cultural backgrounds.

### Statistical analysis

Different analytical approaches were deployed to derive trajectories and accumulations of PA based on repeated measurements. These included categorising PA patterns based on trajectories (eg, increasing, decreasing, and consistent), treating PA as a time-varying exposure, and calculating cumulative/average PA levels from each assessment. As the methodologies were heterogeneous and their results were incomparable, we split our meta-analyses into three parts.

The first part of the meta-analyses is for the studies with trajectory exposures, in which three sub-analyses were performed for ‘consistently active’, ‘increasing PA’ and ‘decreasing PA’. We defined the reference group as ‘consistently inactive’ in all studies. When there were different reference groups, we used the method developed by Orsini^20^ to convert the risk estimates and confidence intervals. Since some studies determined the mortality risks at multiple levels of exposure, we considered the ‘highest versus lowest’ meta-analyses. Among the studies with three or more PA categories, risk estimates were extracted from ‘consistently inactive (lowest PA level)’ (reference group), ‘consistently active (highest PA level)’, ‘increased from lowest to highest PA level’ (increasing PA), and ‘decreased from highest to lowest PA level’ (decreasing PA). If the start or end points of PA patterns were not explicitly stated in multi-level exposure (eg, increase to at least medium PA), the results were excluded from the meta-analyses to avoid the impact of medium PA levels. For studies using group-based trajectory modelling (GBTM), we selected trajectories with distinct patterns such as ‘consistently low’, ‘consistently high’, ‘high-low’, and ‘low-high’ based on categorisations given in papers. The results for fluctuating PA (eg, low-high-low) were excluded from meta-analyses.

The second part of the meta-analyses is for the studies with time-varying and cumulative PA, in which analyses were performed separately for each modelling. In these studies, PA accumulation generated from repeated measurements was used to predict mortality outcomes. Therefore, we used the ‘highest versus lowest’ method and defined the reference group as the lowest PA accumulation. Risk estimates were extracted from the lowest PA accumulation (reference group) and the highest PA accumulation. Studies with continuous PA exposure (n=2) were not included in the analyses.

As the unit of PA varied largely across studies, original PA assessments in different studies were incomparable. To provide a comparable indication of different PA categories across studies, we performed PA data harmonisation for all studies included in the meta-analyses. The comprehensive harmonisation process was adapted from previous reviews,^10 11^ and the flow chart is illustrated in online supplemental figure 2. PA exposures were harmonised into marginal MET-hours per week (mMET.h/week), which is the energy expenditure above resting metabolic rate. Briefly, PA reported in frequency, duration and intensities was converted into weekly duration, assuming 0.75 hour/session. Light, moderate, and vigorous intensities were assigned mMET values of 1.5, 3.5, and 7.0, respectively. For PA reported in energy expenditure, MET equivalents were calculated using the equation: 1 MET=1 kcal/kg/hour. If weight was not reported, we calculated the weight from body mass index (BMI) and height. For PA reported in gross MET values, 1 MET.hour was subtracted for each hour of activity. If duration was unavailable, the conversion equation shown in online supplemental figure 2 was used. WHO recommended levels of PA (150–300 min of moderate-to-vigorous PA per week) were converted to 8.75 and 17.5 mMET.h/week, respectively. Harmonised PA data for each study are shown in our OSF repository.

Based on the harmonised PA data, we conducted the third part of the meta-analyses, which explored the dose-response relationships using studies with three or more PA categories. This is performed because extracting the risk estimates from only the upper and lower levels of exposure can lead to loss of information. For the studies examining trajectory exposures, we extracted data for (1) consistent PA at different levels (eg, consistently low/medium/high); (2) increasing from inactive to different levels of higher PA (eg, low-medium/low-high); and (3) decreasing from different levels of higher PA to inactive (eg, medium-low/high-low). The reference group comprised the people who were consistently inactive. For studies with time-varying and cumulation exposures, data were extracted for PA accumulation at different levels (eg, low/medium/high). For all dose-response gradients, the linearity was tested by the Wald test. If the linearity hypothesis was rejected, we fit the data using non-linear models. When the original publication did not provide the information needed for dose-response analyses (eg, cases in each category), data were found from other studies using the same cohort, by contacting authors, or by the imputation procedures commonly used by previous reviews.^10 11^

Since there has been evidence suggesting a ‘PA paradox’, where PA from different domains may impact health differently,^21^ we stratified our analyses (when applicable) according to total and leisure-time PA to account for potential heterogeneity. The inverse variance weighted method was applied to combine the extracted effect estimates and their 95% CIs. Random-effects models were used to account for the effect of between-study heterogeneity. Heterogeneity was assessed by using the I² statistic and the p value from χ^2^-based Cochran’s Q test. High heterogeneity between studies was defined by I² values >50% or p value <0.10.^22^ Publication bias and small-study effects were evaluated by funnel plots and Egger regression symmetry tests. Since the results from our meta-analyses may have been affected by single studies, we conducted influence analyses to identify the ‘outlying’ studies that might lead to biases and yield misleading results.^23^ Further, we also estimated the potential impact of a new study on our results by displaying statistical contours.^24^ For unmeasured confounding bias, we calculated E-values, which quantified the magnitude of unmeasured confounding capable of reducing the associations to the null.

### Subgroup analysis

We identified potential sources of heterogeneity through prespecified and post hoc subgroup analyses for main associations (ie, PA trajectories with all-cause mortality). Prespecified subgroup analyses were performed based on whether studies adjusted for adiposity-related covariates (eg, BMI, waist circumference, etc) (yes vs no) and excluded the first few years of follow-up (yes vs no). These analyses were based on the evidence that obesity is sometimes considered a mediator between PA and health outcomes,^25^ and that people with severe diseases might have less active behaviour and higher mortality risk, and this reverse causation might affect associations up to 6–8 years of follow-up.^26^ Post hoc subgroup analyses were performed based on: (1) gender composition (0–49% vs 50–100% women); (2) age range at baseline: young-mid age (18–50 years), mid-age (30–69 years), mid-old age (40–92 years), old age (60–96 years), and all ages; (3) the number of PA categories (2 vs ≥3); (4) PA measurement duration (<7 (median) vs ≥7 years) and mortality follow-up duration (<10 (median) vs ≥10 years); (5) study continent (Europe, North America, Asia, and Oceania); and (6) study quality assessment (good, fair, poor). We used the Instrument to assess the Credibility of Effect Modification ANalyses (ICEMAN)^27^ in our subgroup analyses to understand the credibility of subgroup findings. Detailed ICEMAN criteria are shown in our OSF repository.

### Sensitivity analysis

As the main meta-analyses used the ‘highest versus lowest’ approach, which could result in inconsistent definitions of ‘high PA’ and ‘low PA’ across studies, we performed additional analyses based on the harmonised PA data. ‘Low PA’ was defined as below half of the minimum recommended PA level (≤4.4 mMET.h/week) and ‘high PA’ was defined as meeting the guidelines (≥8.75 mMET.h/week). The results were compared with main analyses. Since few studies (n=5) reported risk measures other than HRs, RRs were used as the overall effect estimates in the meta-analyses. To test the robustness of results, a comparison between the main analyses and the results including only HRs was performed. Given the concerns with small study bias^10^ and recent changes in patterns and norms in global PA,^28^ we further performed sensitivity analyses by excluding studies with a sample size <500 and published 20 years ago. Lastly, regarding the concern about the sensitivity of PA questionnaires to detect changing PA levels over time, we performed sensitivity analyses using only the studies with PA questionnaires specifically validated for measuring changes (n=22) and compared these with the main analyses.

The significance level for all statistical analyses was set at 0.05, and all analyses were conducted using Stata 18.0 (StataCorp, College Station, TX, USA).

## Results

### Searching results

**Figure 1:**
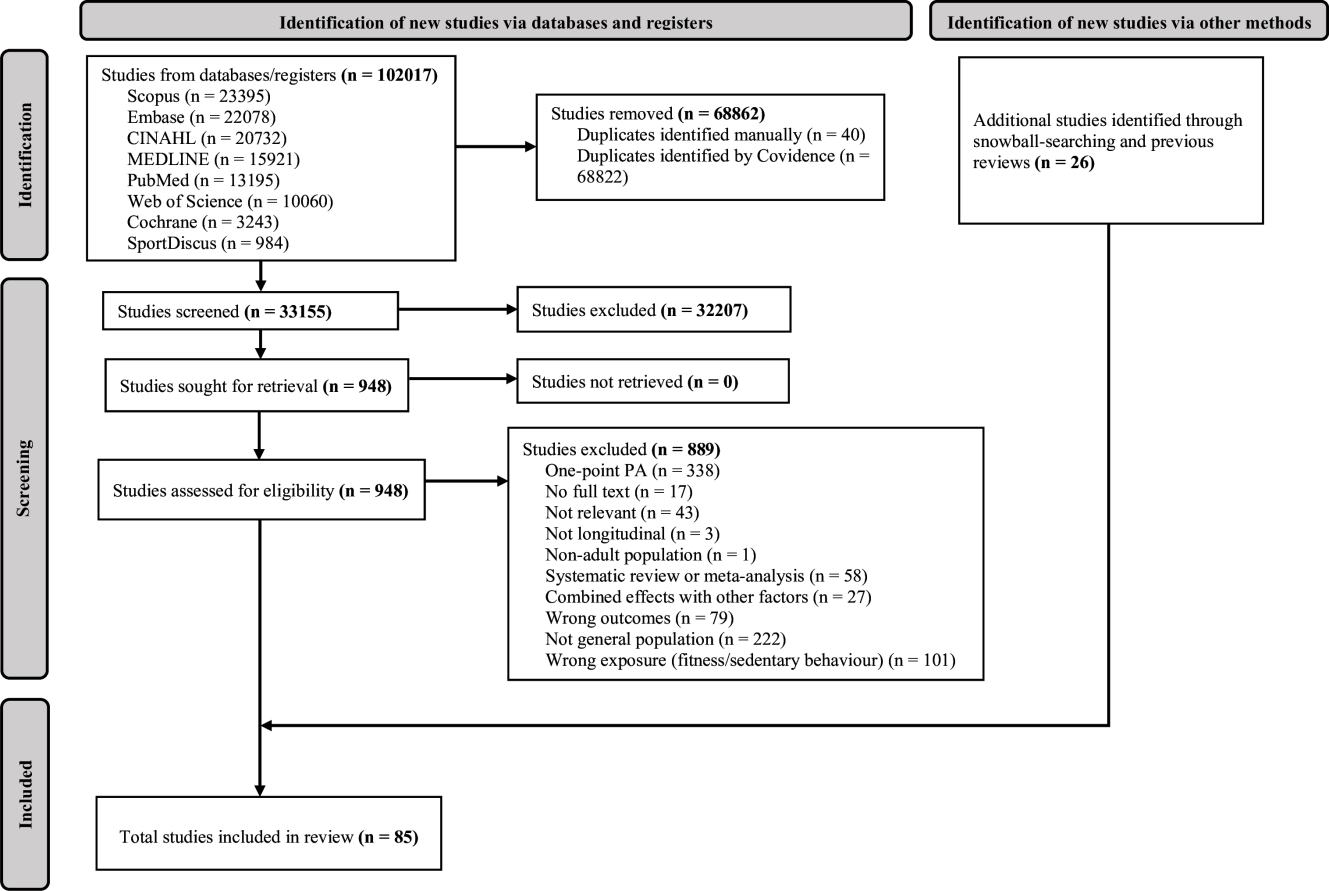
PRISMA (Preferred Reporting Items for Systematic reviews and Meta-Analyses) flow diagram for systematic review.

After removing duplicates, 33 155 potentially relevant titles were identified. Initial screening was based on titles, resulting in 948 papers being selected for further evaluation based on abstracts and full texts. The full-text assessment identified 59 studies. After including 26 additional studies identified from the snowball method and previous reviews, our review included 85 studies in total. The PRISMA (Preferred Reporting Items for Systematic reviews and Meta-Analyses) flow diagram is shown in figure 1. Details of excluded studies were in our OSF repository.

### Characteristics of included studies

The summary characteristics of the included studies are shown in table 1. The detailed characteristics are provided in online supplemental table 3. The types of analytical methodology used in the studies were identified as follows: 59 studies classified longitudinal PA into trajectories;^29^,^87^ 16 studies treated PA as a time-varying exposure and used time-varying Cox regression models;^4083 88^,^101^ and 11 studies analysed PA using average/cumulative scores.^65102^,^111^ Two studies used both trajectory and time-varying exposure modelling,^40 83^ and one study used both trajectory and cumulative PA modelling. PA trajectories were modelled via two approaches: 52 studies categorised PA trajectories into trends (ie, increasing/decreasing/consistent) or adherence/non-adherence to PA guidelines,^30^,^5860 62 63 65^ while seven studies used latent class/GBTM to determine naturally occurring PA trajectories during follow-up periods.^29 59 61 64 73 74 85^ Two studies used neither of the modelling methods, which included adjustment for past PA^112^ and independent analyses for PA at different time points.^113^ Among the included studies, 77 assessed all-cause mortality,^29^,^3638^ 34 assessed CVD mortality,^2931 36 37 41^,^43 46 49 51 52 54 58 59 61 62 65 66 68 69 73 78 79 82 88 90 96 97 99 106^ and 15 assessed cancer mortality.^31 46 51 59 61 73 79 84 88 92 96 99 106 107 110^ The analytical sample size of the individual studies ranged from 357 to 6 572 984. The proportion of women ranged from 7% to 73% among the studies including participants from both genders. Six studies included only women^46 65 80 85 99 106^ and 14 studies were performed in men exclusively.^29 38 40 43 48 50 51 59 71 82 93 97 103 110^ PA was reported in various ways, including hours/minutes per week, MET values, energy expenditure, and others. Five types of age ranges were found among the included studies: young-mid age (18–50 years),^56 80 104^ mid-age (30–69 years),^29 30 36 40 48 50 54 65 70 77 78 82 83 92 97 98 102 103 106 107^ mid-old age (40–92 years),^37 45 47 52 57 64 67 71 73 84 88 93 94 108 110 113^ old-age (60–96 years),^32 34 35 38 44 46 49 51 59 63 74 85 87 100 111 112^ and all ages.^3133 39 41^,^43 53 55 58 60^ Eighty-three studies assessed mortality outcomes via record linkage or death certificates, while two studies did not report the methods of death data collection.^61 81^ Cox regression models were used in 76 studies to derive the HRs of mortality risks. Other effect measures include ORs from generalised estimating equations/logistic regressions (n=2),^48 51^ risk ratios from parametric g-formula (n=2),^83 86^ marginal rate ratios from marginal structural models (n=2),^78 102^ incident rate ratios from Poisson regression (n=2),^71 104^ and event-time ratios from Weibull regression models (n=1).^87^ Some studies used data from the same cohort, in which the Copenhagen City Heart Study (n=5),^33 72 75 76 81^ the HUNT study (n=4),^39 58 66 69^ the Taiwan MJ cohort study (n=4),^31 61 79 109^ and the Aerobics Centre Longitudinal Study (n=3)^43 62 68^ were the most common cohorts in this review. To account for double counting of participants in the meta-analyses, we selected one study from each cohort for each outcome, as shown in online supplemental table 4.

**Table 1:** Summary characteristics of included studies (N=83)*

	Number of studies in each type of modelling		
	Trajectory PA (n=59) n (%)	Time-varying PA (n=16) n (%)	Cumulative/average PA (n=11) n (%)
Outcomes			
All-cause mortality	54 (91.5)	14 (87.5)	10 (90.9)
CVD mortality	24 (40.7)	5 (31.3)	5 (45.5)
Cancer mortality	8 (13.6)	4 (25.0)	3 (27.3)
Sample size range			
<5000	24 (34.6)	5 (31.3)	2 (18.2)
5000–9999	8 (15.4)	3 (18.8)	3 (27.3)
10 000–49 999	18 (32.7)	6 (37.5)	2 (18.2)
≥50 000	9 (17.3)	2 (12.5)	4 (36.4)
Baseline age range			
Young-mid age (18–50 years)	2 (3.4)	0 (0.0)	1 (9.1)
Mid-age (30–69 years)	13 (22.0)	5 (31.3)	5 (45.5)
Mid-old age (40–92 years)	10 (17.0)	3 (18.8)	2 (18.2)
Old age (60–96 years)	13 (22.0)	1 (6.3)	1 (9.1)
All ages	21 (35.6)	7 (43.8)	2 (18.2)
Length of measurement			
<5 years	8 (13.6)	0 (0.0)	1 (9.1)
5–9 years	23 (39.0)	4 (25.0)	3 (27.3)
≥10 years	25 (42.4)	12 (75.0)	7 (63.6)
Not specified	3 (5.1)	0 (0.0)	0 (0.0)
Length of follow-up			
<5 years	5 (8.5)	0 (0.0)	1 (9.1)
5–9 years	18 (30.5)	3 (18.8)	2 (18.2)
≥10 years	35 (59.3)	13 (81.3)	8 (72.7)
Not specified	1 (1.7)	0 (0.0)	0 (0.0)
Percentage of women			
0%	10 (16.9)	3 (18.8)	2 (18.2)
1–49%	12 (20.3)	5 (31.3)	0 (0.0)
50–99%	32 (54.2)	7 (43.8)	7 (63.6)
100%	4 (6.8)	1 (6.3)	2 (18.2)
Not specified	1 (1.7)	0 (0.0)	0 (0.0)
Study region			
Europe	34 (57.6)	7 (43.8)	7 (63.6)
North America	14 (23.7)	7 (43.8)	3 (27.3)
Asia	7 (11.9)	1 (6.3)	1 (9.1)
Oceania	4 (6.8)	1 (6.3)	0 (0.0)
Study quality			
Good	28 (47.5)	7 (43.8)	5 (45.5)
Fair	13 (22.0)	2 (12.5)	4 (36.4)
Poor	18 (30.5)	7 (43.8)	2 (18.2)

* Two studies using neither of the three modellings are not included. Details shown in Supplementary Table 3online supplemental table 3.

CVD, cardiovascular disease; PA, physical activity.

Only four studies used hypothetical intervention methods,^78 83 86 102^ which included g-formula and marginal structural models to determine the risk ratios from different hypothetical scenarios, such as being consistently active or initiating PA at various time points.

### Potential bias and quality assessment

The results of the risk of bias assessment are shown in online supplemental table 2B.

Among the included studies, 38 studies were of good quality in risk of bias assessment,^3033 39 40 43^,^45 47 51 53 58 60 63^ 19 were of fair quality,^3134 37 46 49 54^,^57 59 71 78 82 95 99 102 105 106 111^ and 28 were of relatively poor quality.^2932 35 36 38 41 42 48 50 52 61 62 70 75 77 81 84 85 91^,^94 96^ The major source of potential bias was the lack of adjustment for time-varying confounding. Around 44% of studies (38 out of 85) accounted for reverse causation by excluding the outcomes from the first few years of follow-up.^2930 37 40^,^45 47 51^

### Consistently active and all-cause mortality

**Figure 2:**
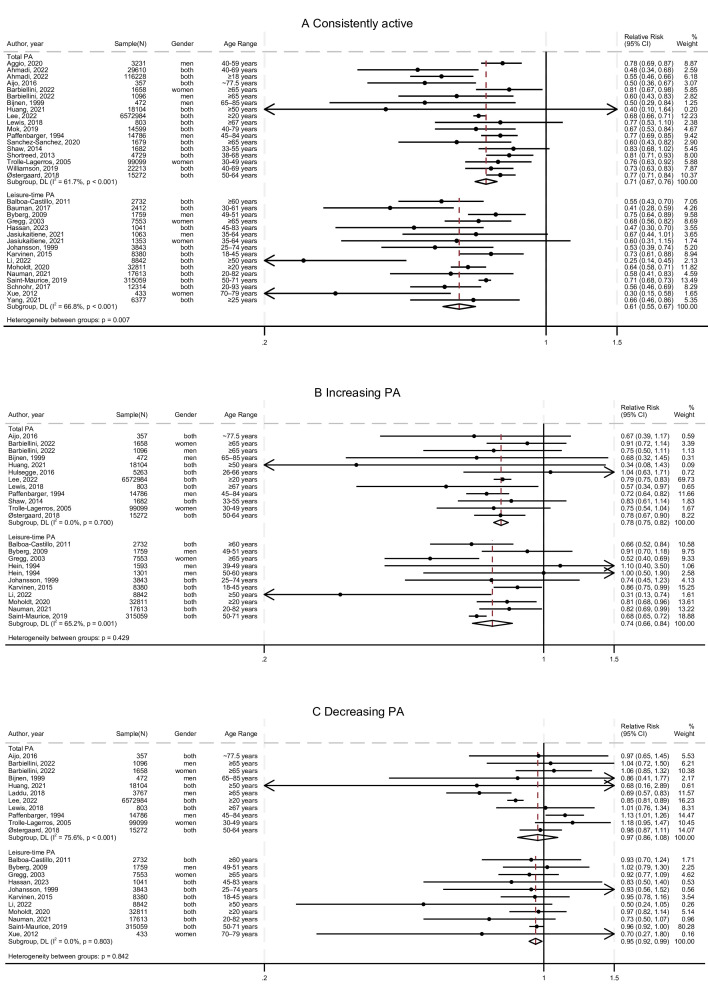
Meta-analyses of the associations between (A) consistently active, (B) increasing physical activity patterns, and (C) decreasing physical activity patterns and all-cause mortality, in comparison to consistently inactive. DL, DerSimonian-Laird random-effects model; PA, physical activity.

The meta-analyses examining the associations between consistent activity and all-cause mortality included 32 studies.^29^,^3234^ The summary relationships are illustrated in figure 2A. As suggested by the results, consistently active levels of total PA were associated with 29% lower risk of all-cause mortality (RR 0.71, 95% CI 0.67 to 0.76), while consistently active levels of leisure-time PA were associated with 39% lower risk (RR 0.61, 95% CI 0.55 to 0.67). The p value for the difference between domains is 0.007, suggesting that PA domains might be a source of heterogeneity. However, a high level of between-study heterogeneity was observed within each of the PA domains (total PA: I^2^=61.7%; leisure-time PA: I^2^=66.8%), making the validity of the difference between PA domains uncertain.

### Increasing PA and all-cause mortality

The meta-analyses examining the associations between increasing activity and all-cause mortality included 21 studies,^32 34 35 38 40 46 48 52 53 55 56 60 63 64 66 68 70 71 73 77 80^ as shown in figure 2B. Participants who increased from inactive to active levels of total PA had 22% lower risk of all-cause mortality compared with those who remained inactive (RR 0.78, 95% CI 0.75 to 0.82), while those who increased their leisure-time PA had 27% lower risk (RR 0.74, 95% CI 0.66 to 0.84). The level of heterogeneity (I^2^) was 0.0% for total PA and 65.2% for leisure-time PA.

### Decreasing PA and all-cause mortality

The meta-analyses examining the associations between decreasing activity and all-cause mortality included 21 studies,^32 34 35 38 40 46 47 52 55 56 59 60 63 64 66 68 70 71 73 80 85^ as shown in figure 2C. The participants who decreased their total PA levels from active to inactive did not have lower risk in all-cause mortality (RR 0.97, 95% CI 0.86 to 1.08). The risk reduction shown in decreasing patterns of leisure-time PA was very limited and close to null (RR 0.95, 95% CI 0.92 to 0.99). The value of I^2^ was very low (0.0%) between studies measuring leisure-time PA, but high (75.6%) between studies measuring total PA.

For the above associations, the impact of the addition of a new study was assessed via the funnel contours, as illustrated in online supplemental figure 3. As shown, all studies for consistent and increasing PA lay in the region of statistical significance, suggesting that their results are relatively robust to the addition of new studies, while the results for decreasing PA patterns seemed less robust. The influential analyses illustrated that the summary results of our meta-analyses were unlikely to be affected by a single outlying study. There was no evidence for publication bias in increasing/decreasing patterns, while the Egger’s test suggested potential publication bias in the association between being consistently active and all-cause mortality.

### Time-varying and cumulative PA and all-cause mortality

**Figure 3:**
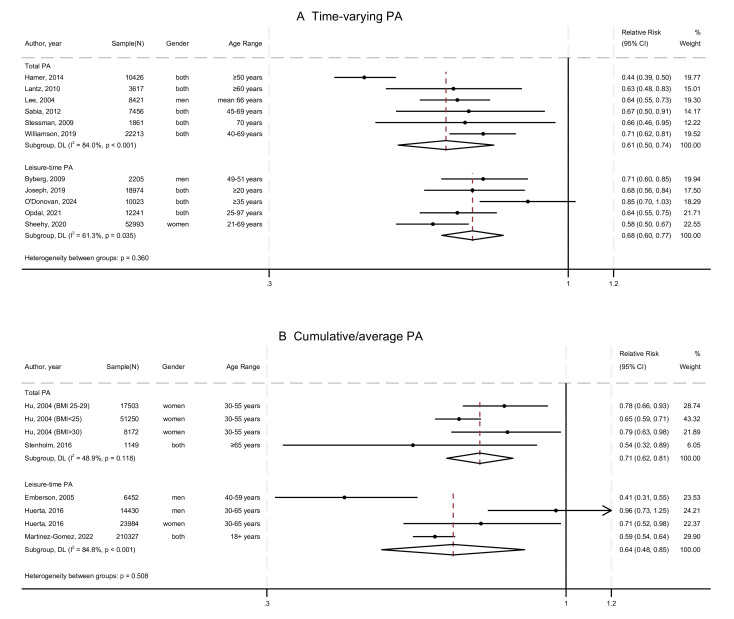
Meta-analysis of the associations between higher physical activity levels and all-cause mortality among studies with (A) time-varying and (B) cumulative/average physical activity exposure, in comparison to inactivity. DL DerSimonian-Laird random-effects model; PA, physical activity.

Figure 3 illustrates the results of the studies with (A) time-varying^4083 88 89 91 93^,^95 98^ and (B) cumulative/average^103 106 107 109 111^ PA exposures, respectively. As shown, higher PA was associated with lower risk of all-cause mortality than being inactive. Both time-varying and cumulative exposure suggested 30–40% lower risk for people who had high levels of PA. However, the summary associations might not be robust since the number of studies was limited (n=11 for time-varying PA; n=5 for cumulative PA), and the summary results for cumulative PA were mostly derived from Hu *et al*^106^ and Huerta *et al*.^107^

### Associations with cause-specific mortality

We performed additional analyses for PA patterns and CVD^29 31 36 37 41 42 46 49 52 54 59 68 73 75 78^ and cancer mortality,^31 46 59 73 84^ which are illustrated in online supplemental figures 4 and 5. Generally, the association between high PA and a reduced risk of mortality was more evident for CVD than cancer mortality. Compared with participants who were consistently inactive over time, those who were consistently active in either total or leisure-time domain had around 40% and 25% lower risk for CVD and cancer mortality, respectively. Inverse associations were shown in increasing PA in relation to CVD mortality, but not cancer mortality. The health benefits of decreasing PA patterns had a high level of uncertainty and were quite limited for both CVD and cancer mortality. In general, the evidence base for the associations between PA patterns and cause-specific mortality remained limited and inconclusive, especially for cancer mortality.

The results for time-varying and cumulative/average PA with CVD^88 99 106 107 109^ and cancer mortality^88 92 99 106 107 110^ are shown in online supplemental figures 6 and 7, which suggest a similar trend that the association between higher PA and lower mortality risk was more pronounced for CVD mortality than cancer mortality. However, since there were limited studies for cause-specific mortality, the magnitude of the observed associations might be affected by the addition of new studies. For all associations determined, the computed E-values are shown in online supplemental table 5. E-values were relatively high for the associations between being active with all-cause and CVD mortality, while the E-values were close to null (1) for decreasing PA patterns and cancer mortality.

### Dose-response relationship

**Figure 4:**
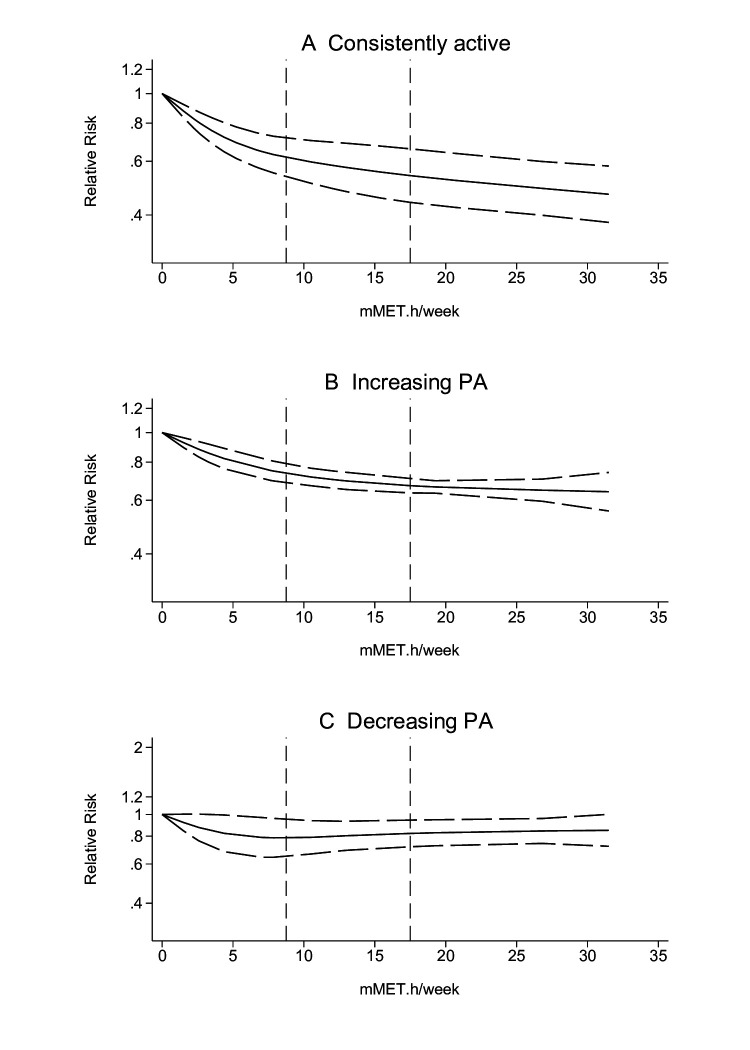
Dose-response associations between (A) consistently active, (B) increasing physical activity, and (C) decreasing physical activity patterns and all-cause mortality, in comparison to consistently inactive. Models adjusted for physical activity domains, trajectory years and follow-up years. Dotted lines are 95% CIs. Vertical lines at 8.75 and 17.5 mMET.h/week, respectively. PA, physical activity.

The dose-response analyses were performed for the associations in which four or more independent studies were included. The dose-response gradients between different PA patterns and all-cause mortality are shown in figure 4 for (A) consistently active (n=9);^3147 52 66^,^68 73 76 85^ (B) increasing PA (n=7);^33 42 52 66 68 73 76^ and (C) decreasing PA (n=5).^52 66 68 73 76^ Due to the limited number of studies, dose-response gradients could not be stratified into total and leisure-time domains. Inverse, curvilinear dose-response associations were observed for consistently active and increasing PA patterns. The dose-response associations were more evident from 0 to around 8.75 mMET.h/week. Compared with being consistently inactive, being consistently active at 8.75 mMET.h/week was associated with around 40% lower risk of all-cause mortality, and the risk reduction increased to around 46% at the upper bound of the recommended level (17.5 mMET.h/week). However, a further increase in PA levels to 30 mMET.h/week only had ~5% additional risk reduction.

Increasing PA from previously being inactive was also strongly associated with a lower mortality risk until the lower bound of recommended PA (8.75 mMET.h/week). Compared with being consistently inactive, increasing from 0 to 8.75 mMET.h/week was associated with around 25% lower risk of all-cause mortality; however, additional increases beyond 17.5 mMET.h/week did not seem to have additional risk reductions. Some risk reductions were also seen in the decreasing PA patterns, although the upper limit of confidence interval was close to null. Due to the limited number of included studies, the results were exploratory.

The dose-response gradients for time-varying^40 88 89 93 95 98 99^ and cumulative^103106 107 109^,^111^ PA volumes with all-cause mortality are shown in online supplemental figure 8. As illustrated, the risk of mortality reduced with increased PA volumes in both time-varying and cumulative PA, where meeting PA guidelines was associated with a 30–40% lower risk of all-cause mortality. Online supplemental figure 9 illustrates the dose-response associations between consistently active,^31 52 66 68 73 75^ increasing,^42 52 66 68 73^ and decreasing PA^52 66 68 73^ with CVD mortality. The results were similar to all-cause mortality for people with consistent or increasing PA; however, limited health benefits were shown on CVD mortality from decreasing PA patterns.

### Subgroup analyses

The results of subgroup analyses and assessment of credibility are shown in online supplemental tables 6 and 7. They suggested potential heterogeneity sources in this review were the number of PA categories, PA measurement duration, and mortality follow-up duration (moderate credibility). Generally, weaker associations were observed in binary PA categorisation, and longer lengths of exposure measurement and mortality follow-up in any of the PA patterns. The heterogeneity between age groups was significant in those who were consistently/increasingly active (p=0.03), with older individuals (ie, mid-old/old age) experiencing greater reductions in mortality risk than people in earlier life stages (eg, young-mid age). Heterogeneity was also observed across studies conducted in different continents, in which studies from Asia indicated stronger associations than those from other regions. However, the differences between age and region subgroups were of low credibility due to limited study numbers, so these subgroup results remained uncertain.

### Sensitivity analysis

Among a total of 56 studies included in meta-analyses, the PA units in 50 studies were able to be harmonised into mMET.h/week. Studies with harmonised PA levels were included in additional analyses for ‘low PA (≤ 4.4 mMET.h/week)’ versus ‘high PA (≥ 8.75 mMET.h/week)’, as shown in online supplemental figures 10 and 11. The results aligned with the main analyses. Excluding studies with risk measures other than HR did not alter the estimates from main analyses, as illustrated in online supplemental table 8. Further, results excluding studies with sample size <500 and published 20 years ago (online supplemental table 9), and including only studies specifically validated to measure changes (online supplemental tables 10A, 10B and 10C), also did not alter our main findings.

## Discussion

Our study reviewed the associations between long-term PA patterns and accumulation during adulthood and mortality risks later in life. It was the first and currently largest study to provide a meta-analysis quantifying risk reduction for different mortality outcomes based on various PA patterns, incorporating stratified methodologies. As determined, being consistently or increasingly active was associated with around 20–40% risk reduction in all-cause mortality and 30–40% risk reduction in CVD mortality. While the evidence of decreasing PA patterns remains inconclusive, our results indicate the number of PA categories may influence observed associations, which highlights key implications for future PA measurement and analysis. Through exploring different types of PA modelling and their associations with mortality outcomes, our review identified various ways to deal with longitudinal PA data. Further, this is also the first review that explored the dose-response gradients between different patterns of PA and mortality risks.

Several mechanisms may explain the reduced mortality risk with high PA, such as physical function improvement and anti-inflammatory effects.^114^ Greater risk reduction was observed in CVD mortality with consistent/increasing PA than cancer mortality. One explanation is that PA improves cardiovascular fitness, and it can directly affect the function and structure of the vascular system,^115^ which helps reduce cardiovascular risk. Cancers may respond differently to PA,^116^ and some cancers were found not to be associated with PA levels.^11^ In the main analyses, limited benefits were observed between decreasing PA patterns and mortality risk. However, subgroup analyses by the number of PA categories suggested that studies with more than three PA categories indicated a risk reduction of 17% (3–30%) between decreasing PA and all-cause mortality. This suggests a possibility that binary classifications of PA levels might dilute the ‘highest versus lowest’ associations, potentially leading to underestimation of the benefits. This finding partially supports the ‘bank saving’ hypothesis,^117^ which states that previously accumulated PA could bring some health benefits.

The associations between PA and mortality risks might vary depending on the PA domains. Our results suggested that risk reductions were more evident in people who were consistently active in leisure time than those with consistently high total PA levels. Although this difference requires further exploration, one possible explanation is that people who reported a high level of total PA might accumulate most of their daily PA at work, which may not provide the same health benefits as leisure-time PA, as suggested in a previous review.^21^ As the majority of PA studies only examined total and leisure-time PA, the summary associations between other PA domains (eg, occupational/household) and mortality still require further exploration.

The major sources of heterogeneity in this study came from the number of PA categories, the length of PA measurement and the length of follow-up for mortality. Stronger associations were observed in studies with more than three PA categories than those with two categories. Risk reductions decreased with longer lengths of PA measurement and mortality follow-up. This finding is consistent with the results from previous studies,^11 118^ suggesting that shorter follow-up periods may be more affected by reverse causation. Analyses by age groups suggested health benefits of PA in all age groups, with stronger associations observed in the old age group than the young-mid age group. This aligns with a previous finding that the inverse association between PA and mortality was greater as age increased;^119^ however, this difference requires further exploration. Our sensitivity analyses suggested that our results were relatively robust. E-values provided evidence for unmeasured confounding bias. The inverse associations between being consistently/increasingly active with all-cause and CVD mortality had relatively high E-values (≥2) and thus were less likely to be affected by unmeasured confounders. However, the associations for decreasing PA patterns and cancer mortality had E-values and confidence limits close to null (1), so their results were less robust due to unmeasured confounders. This implied relatively strong associations between being consistently/increasingly active and a lower risk of all-cause and CVD death.

The dose-response analyses estimated the non-linear gradients between PA volumes and the risk of mortality outcomes across various PA patterns. Our results suggested risk reductions among people consistently adhering to PA guidelines or increasing PA to the guidelines, compared with those being consistently inactive. In both consistent and increasing PA patterns, the most substantial risk reduction was observed up to 8.75 mMET.h/week, while additional PA beyond the recommended levels might not bring substantial additional benefits. This aligns with the evidence that PA levels lower than the guidelines can also bring appreciable health benefits,^39^ and the statement that some PA is always better than none.^2^ Notably, as compared with the effect estimates from previous reviews for meeting PA guidelines (15–30% risk reduction compared with inactivity),^11 120 121^ which were mostly based on one-time-point PA measurement, our results showed relatively stronger risk reductions in all-cause and CVD mortality with consistent adherence to PA guidelines (~40% risk reduction). This suggests potential underestimation of associations with one-time-point PA measurements and aligns with the hypothesis that repeated measurements of self-reported PA may show stronger and more robust effect estimates than single measurements.^122^

### Strengths and limitations

Our review has important strengths. First, our review searched eight databases and identified over 80 relevant studies, making it a comprehensive summary of current literature in this field. This is also the first study to perform meta-analyses for the associations between PA patterns and mortality risks. While a previous systematic review summarised these associations,^3^ we further quantified the effect estimates using meta-analyses, facilitating a detailed comparison between different trajectories and accumulation of PA across adulthood. Specifically, our review contributes to the literature about the associations between consistent, increasing and decreasing PA at different adulthood stages with mortality risks, enabling more accurate estimation of mortality risks for people with different PA patterns. We performed separate meta-analyses for three types of PA modelling, despite the studies investigating time-varying and cumulative exposures being limited. Additionally, we conducted thorough sensitivity analyses, which suggested that our findings were relatively robust. Through a comprehensive data harmonisation process, we facilitated between-study comparisons and enabled dose-response analyses across studies with various PA units.

Despite these strengths, this study has several limitations. First, most of our included studies used self-reported PA, which may have led to measurement error since the participants were likely to have recall bias. Although most of the studies used validated questionnaires, Ekelund *et al* suggested that questionnaire-based data might underestimate the associations when compared with more precise device-measured data.^123^ However, the wide use of device measurement could be challenging in large population-based observational studies^124^ and may also be at substantial risk of sample selection bias.^125^ Although there are concerns about the sensitivity of PA questionnaires to detect changes, a range of PA questionnaires has been shown with acceptable sensitivity to detect PA changes over time,^126^,^128^ indicating that they are an effective approach to assess PA patterns, especially when device-based measurements are not feasible.^129^ Some questionnaires (eg, PA questionnaire in the HUNT study)^130^ have shown relatively low validity in assessing light-intensity PA. However, the studies in this review mostly examined overall PA volume or moderate-to-vigorous PA, both of which are generally considered to be more accurately captured by self-reported questionnaires than light-intensity PA.^131^ Sensitivity analyses restricted to studies utilising PA questionnaires with established validity for assessing overall PA and moderate-to-vigorous PA, as well as for detecting changes in PA, illustrated similar results with the main analyses. Therefore, we believe that these limitations are unlikely to affect substantially the integrity and robustness of our findings. The other concern is related to residual confounders like frailty and diet, and the inconsistent level of covariate adjustment across studies. The high level of heterogeneity also has an impact on the comparability between studies and the summarisation of effect estimates. Since these studies varied largely in their exposure assessment and classification, the results of the ‘highest versus lowest’ meta-analyses might be influenced by relatively medium PA levels. However, in our supplementary analyses comparing harmonised PA levels for low (≤4.4 mMET.h/week) and high (≥8.75 mMET.h/week), our findings remained consistent. In addition, most of the studies looking at PA trajectories had relatively long duration of follow-up for mortality data, during which period PA was no longer assessed. Therefore, any changes in PA during the mortality follow-up were not accounted for in the analysis. Another limitation is due to the small number of studies with time-varying and cumulative exposures, and for cancer mortality outcomes, which limited the robustness of their summary estimates.

Time-varying confounding is another concern, which was also suggested by Yang *et al*.^3^ Since we were investigating the associations between longitudinal exposure and its outcomes, confounders like disease status and BMI were likely to change over time; therefore, controlling solely for baseline covariates might not be sufficient to determine the true association. Although some studies adjusted for time-varying covariates, underestimation might also occur since some covariates could be affected by the PA at previous time points. One appropriate way to solve this issue is to use g-methods,^132^ which were adopted by only four studies.

The dose-response analyses included a limited number of studies. However, our results are exploratory and can be developed further by future studies. Although the associations could be influenced by PA domains, length of PA measurement and length of follow-up, we were unable to stratify and compare the dose-response gradients by these factors due to limited studies. The dose-response gradients for cancer mortality could not be determined either. Additionally, although PA levels were harmonised to enhance comparability, some values were estimated based on assumed duration, frequency and intensity. This raised the level of uncertainty and might result in the underestimation of the associations.^133^ Moreover, due to the observational design of the included studies, we cannot conclude about the causal association between PA patterns and mortality, although the relatively high E-values implied relatively strong associations for consistent and increasing PA.

### Future studies

Our review identified three general ways to model longitudinal PA measurements; however, which methods provide better predictions of health outcomes is still under-explored. Therefore, future studies should consider comparing different methods of longitudinal PA modelling for their performance in predicting mortality and a range of health issues. The results from our review also suggest the need for additional investigations into the field of cumulative PA and cause-specific mortality. Further, given that current research has not made sufficient adjustments for time-varying confounding, future studies could adopt hypothetical interventions like g-methods to provide results with a lower risk of confounding bias. One study performed by Laddu *et al* adjusted for the PA levels at the latest follow-up examination,^59^ where the associations were attenuated to null; therefore, whether it is the PA trajectory or the most recent PA levels that are associated with mortality outcomes still remains under-explored. In addition, since self-report questionnaires struggle to capture light-intensity and intermittent activities,^11^ future studies should consider the use of devices like accelerometers to obtain more accurate PA measurements and more specific PA changing patterns. Considering the high costs and potential selection bias related to device-based measurements, one approach for future studies is to use device-measured PA as a reference standard on a sample of participants. This would help calibrate self-report PA categories through more precise estimates of actual PA levels,^129^ which would benefit both data interpretation and between-study comparisons. Moreover, our review found a potential association between decreasing PA patterns and mortality risks. However, this association was only observed in studies with more than three PA categories. Therefore, future research might explore how different classifications of PA impact associations in different PA patterns. Lastly, as the associations became weaker with longer follow-up periods, possibly due to reverse causation,^118^ future research should consider performing sensitivity analyses excluding cases from the first few years of follow-up to reduce potential bias from reverse causation.

### Public health implications

Our findings have important public health implications. First, our results emphasised the importance of PA across adulthood, indicating that initiating PA at any point in adulthood may provide survival benefits. Further, our additional analyses illustrated potential risk reductions for people with decreasing PA patterns, which suggested a possibility of the ‘bank-saving’ hypothesis, that previously accumulated PA might provide health benefits in later life stages.^117^ However, as the risk reduction decreased with increasing follow-up periods, the possibility of reverse causation cannot be ruled out. As being consistently active provides greater health benefits than being previously active (ie, no longer maintaining activity), this highlights the importance of sustained PA over time. Future PA interventions may not only target inactive people, but also support active people to maintain their activity. The findings from our dose-response analyses support the validity and effectiveness of current PA guidelines. While it is illustrated that consistent and increasing PA below the guidelines was also associated with survival benefits, our findings would be meaningful for older adults or individuals who might find it challenging to meet PA guidelines.

## Conclusion

Our meta-analyses suggest that consistent and increasing PA (total or leisure-time) were associated with around 20–40% lower risk of all-cause mortality and 30–40% lower risk of CVD mortality, while the association was uncertain for cancer mortality outcomes. A decreasing trend in PA (from active to inactive) might be associated with lower mortality risks, although there was a high level of uncertainty. Adhering to PA guidelines over time or increasing PA volumes to meet the guidelines was associated with reductions in mortality risks, but keeping active or increasing PA below the guidelines can also bring appreciable health benefits for individuals who are inactive. Our findings highlight the health impacts of long-term PA patterns, which have been concealed by research using one-time-point PA measurements in previous years. By capturing these patterns, this review may provide valuable insights for future research and public interventions aiming at promoting sustained PA in adults.

## Supplementary material

10.1136/bjsports-2024-109122 online supplemental file 1

10.1136/bjsports-2024-109122 online supplemental file 2

## Data Availability

Data are available upon reasonable request.
