# Supplementary material for: Physical activity trajectories and accumulation over adulthood and their associations with all-cause and cause-specific mortality: a systematic review and meta-analysis
Source: Br J Sports Med. 2025 Jul 10;59(17):e109122. doi: 10.1136/bjsports-2024-109122 (PMC12418563; doi:10.1136/bjsports-2024-109122)
Supplement: online supplemental file 1 [file bjsports-59-17-s001.pdf]

Supplementary Tables

Supplementary Table 1A. Searching terms and key words. 2

Supplementary Table 1B. Searching strategy in eight databases. 2

Supplementary Table 2A. Risk assessment scale. 5

Supplementary Table 2B. Risk bias assessment of included studies. 6

Supplementary Table 3. Basic characteristics of included studies. 9

Supplementary Table 4. Studies chosen from the same cohort for meta-analyses. 26

Supplementary Table 5. E-values of associations. 27

Supplementary Table 6. Sub-group analyses by potential sources of heterogeneity, for associations between PA trajectories and all-cause mortality. 28

Supplementary Table 7. Assessment of credibility of subgroup difference for the association between PA patterns and all-cause mortality based on ICEMAN. 30

Supplementary Table 8. Comparison between main analyses and studies only reporting hazard ratios. 32

Supplementary Table 9. Comparison between main analyses and results excluding sample size<500 and published before 2003 33

Supplementary Table 10A. Summary of PA questionnaires on validity and ability to detect changes over time. 34

Supplementary Table 10B. Evidence for 1) validity of questionnaires (in comparison to device-based measurements) to assess overall PA volume and MVPA; and 2) validity/ability of questionnaires to detect PA change. 36

Supplementary Table 10C. Comparison between main analyses and analyses using only the studies showing validity for overall PA/MVPA and ability to detect potential changes. 38

Supplementary Table 1A. Searching terms and key words.

Physical activity	Mortality	Longitudinal exposure	Study design
"Exercise"[MeSH]<br>"physical activity"<br>"physical inactivity"<br>"exercise"<br>"physical activities"<br>"physically active"<br>"physically inactive"	"Mortality"[MeSH]<br>"Death"[MeSH]<br>"Survival Rate"[MeSH]<br>"mortality"<br>"death"<br>"survival rate"	"chang*"<br>"increas*"<br>"decreas*"<br>"maintenance"<br>"remained"<br>"became"<br>"trajectory"<br>"trajectories"<br>"lifespan"<br>"life course"	"Cohort Studies"[MeSH]<br>"Prospective Studies"[MeSH]<br>"Longitudinal Studies"[MeSH]<br>"cohort"<br>"prospective"<br>"longitudinal"

Supplementary Table 1B. Searching strategy in eight databases.

**PubMed**		
1	Physical activity	"Exercise"[MeSH] OR "physical activity"[All Fields] OR "physical inactivity"[All Fields] OR "exercise"[All Fields] OR "physical activities"[All Fields] OR "physically active"[All Fields] OR "physically inactive"[All Fields]
2	All-cause/cause-specific mortality	"Mortality"[MeSH] OR "Death"[MeSH] OR "Survival Rate"[MeSH] OR "mortality"[All Fields] OR "death"[All Fields] OR "survival rate"[All Fields]
3	Longitudinal exposure	"chang*"[All Fields] OR "increas*"[All Fields] OR "decreas*"[All Fields] OR "maintenance"[All Fields] OR "remained"[All Fields] OR "became"[All Fields] OR "trajectory"[All Fields] OR " trajectories"[All Fields] OR "lifespan"[All Fields] OR "life course"[All Fields]
4	Study design	"Cohort Studies"[MeSH] OR "Prospective Studies"[MeSH] OR "Longitudinal Studies"[MeSH] OR "cohort"[All Fields] OR "prospective"[All Fields] OR "longitudinal"[All Fields]
		1 AND 2 AND 3 AND 4
**EMBASE**		
1	Physical activity	'exercise'/exp OR 'physical activity' OR 'physical inactivity' OR 'exercise' OR 'physically active' OR 'physically inactive' OR 'physical activities'
2	All-cause/cause-specific mortality	'mortality'/exp OR 'death'/exp OR 'survival rate'/exp OR mortality OR death OR 'survival rate'
3	Longitudinal exposure	chang* OR increas* OR decreas* OR 'maintenance' OR 'remained' OR 'became' OR trajectory OR trajectories OR lifespan OR 'life course'
4	Study design	'cohort studies'/exp OR 'prospective studies'/exp OR 'longitudinal studies'/exp OR cohort OR prospective OR longitudinal
		1 AND 2 AND 3 AND 4
**Scopus**		
1	Physical activity	TITLE-ABS-KEY ("exercise") OR TITLE-ABS-KEY ("physical activity") OR TITLE-ABS-KEY ("physical activities") OR TITLE-

		ABS-KEY ("physically active") OR TITLE-ABS-KEY ("physical inactivity") OR TITLE-ABS-KEY ("physically inactive")
2	All-cause/cause-specific mortality	TITLE-ABS-KEY (mortality) OR TITLE-ABS-KEY (death) OR TITLE-ABS-KEY ("survival rate")
3	Longitudinal exposure	TITLE-ABS-KEY (chang*) OR TITLE-ABS-KEY (increas*) OR TITLE-ABS-KEY (decreas*) OR TITLE-ABS-KEY (maintenance) OR TITLE-ABS-KEY (remained) OR TITLE-ABS-KEY (became) OR TITLE-ABS-KEY (trajectory) OR TITLE-ABS-KEY (trajectories) OR TITLE-ABS-KEY (lifespan) OR TITLE-ABS-KEY ("life course")
4	Study design	TITLE-ABS-KEY (cohort) OR TITLE-ABS-KEY (prospective) OR TITLE-ABS-KEY (longitudinal)
		1 AND 2 AND 3 AND 4
**Web of Science**		
1	Physical activity	ALL=("exercise" OR "physical activity" OR "physical inactivity" OR "physically active" OR "physically inactive" OR "physical activities")
2	All-cause/cause-specific mortality	ALL=(mortality OR death OR "survival rate")
3	Longitudinal exposure	ALL=(chang* OR increas* OR decreas* OR maintenance OR remained OR became OR trajectory OR trajectories OR lifespan OR "life course")
4	Study design	ALL=(cohort OR prospective OR longitudinal)
		1 AND 2 AND 3 AND 4
**Cochrane**		
1	Physical activity	"Exercise"[MeSH] OR (exercise) OR (physical activity) OR (physical inactivity) OR (physically active) OR (physically inactive) OR (physical activities)
2	All-cause/cause-specific mortality	"Mortality"[MeSH] OR "Death"[MeSH] OR "Survival Rate"[MeSH] OR (mortality) OR (death) OR (survival rate)
3	Longitudinal exposure	(chang*) OR (increas*) OR (decreas*) OR (maintenance) OR (remained) OR (became) OR (trajectory) OR (trajectories) OR (lifespan) OR (life course)
4	Study design	"Cohort Studies"[MeSH] OR "Prospective Studies"[MeSH] OR "Longitudinal Studies"[MeSH] OR (cohort) OR (prospective) OR (longitudinal)
		1 AND 2 AND 3 AND 4
**MEDLINE, CINAHL, SportDiscus**		
1	Physical activity	(MH "Exercise") OR (TX "exercise" OR "physical activity" OR "physical inactivity" OR "physically active" OR "physically inactive" OR "physical activities")
2	All-cause/cause-specific mortality	(MH "Mortality") OR (MH "Death") OR (MH "Survival Rate") OR (TX mortality OR death OR "survival rate")

3	Longitudinal exposure	(TI (chang* OR increas* OR decreas* OR maintenance OR remained OR became OR trajectory OR trajectories OR lifespan OR “life course”)) OR (AB (chang* OR increas* OR decreas* OR maintenance OR remained OR became OR trajectory OR trajectories OR lifespan OR “life course”))
4	Study design	(MH "Cohort Studies") OR (MH "Prospective Studies") OR (MH "Longitudinal Studies") OR (TX cohort OR prospective OR longitudinal)
		1 AND 2 AND 3 AND 4

Supplementary Table 2A. Risk assessment scale.

**Selection**	
1) Representativeness of the exposed cohort	a) Truly representative – random sampling & probability sampling (*)<br>b) Somewhat representative – random sampling only (*)<br>c) Selected group<br>d) No description
2) Selection of the non-exposed cohort	a) From the same community as exposed cohort (*)<br>b) From a different source<br>c) No description
3) Ascertainment of exposure	a) Secure record (*)<br>b) Structured interview (*)<br>c) Validated self-report questionnaire (*)<br>d) Not validated questionnaire<br>e) No description
4) Outcomes of interest were excluded from the first few years of follow-up<br>OR<br>Outcomes of interest were excluded from the first few years of follow-up in sensitivity analysis, which did not change the main results	a) Yes (*)<br>b) No
**Comparability**	
1) The study controlled for sociodemographic factors, baseline health status, lifestyle factors and adiposity.	a) Yes (*)<br>b) No
2) Hypothetical intervention approaches used (e.g., g-methods) to control time-varying confounding.	a) Yes (*)<br>b) No
**Outcome**	
1) Assessment of outcome	a) Independent blind assessment (*)<br>b) Record linkage (*)<br>c) No description
2) Was follow-up long enough for outcome to occur? (≥5 years)	a) Yes (*)<br>b) No
3) Adequacy of follow-up cohorts	a) Complete follow-up (*)<br>b) Lost to follow-up not likely to induce bias, or <20% (*)<br>c) lost to follow-up very likely to induce bias, or >20%<br>d) No description

Supplementary Table 2B. Risk bias assessment of included studies.

Study	Selection				Comparability		Outcome			Study quality
	1	2	3	4	1	2	1	2	3	
Aggio, 2020	-	*	*	*	-	-	*	*	-	Poor
Ahmadi, 2022	*	*	*	*	*	-	*	*	-	Good
Ahmadi, 2022	-	*	*	-	*	-	*	*	-	Fair
Aijo, 2016	*	*	*	-	-	-	*	*	*	Poor
Andersen, 2004	*	*	*	-	*	-	*	*	*	Good
Balboa-Castillo, 2011	*	*	-	-	*	-	*	*	-	Fair
Barbiellini, 2022	*	*	-	-	-	-	*	*	*	Poor
Bauman, 2017	*	*	*	-	-	-	*	*	-	Poor
Bergwall, 2021	-	*	-	*	*	-	*	*	*	Fair
Bijnen, 1999	*	*	*	-	-	-	*	*	*	Poor
Breidablik, 2023	*	*	*	-	*	-	*	*	-	Good
Byberg, 2009	*	*	*	*	*	-	*	*	-	Good
Cheema, 2023	-	*	-	*	-	-	*	*	-	Poor
Choi, 2022	*	*	*	*	*	-	*	-	-	Poor
Coelho-Ravagnani, 2021	-	*	*	*	*	-	*	*	-	Good
Duarte Junior, 2024	*	*	*	*	*	-	*	*	-	Good
Dwyer, 2015	*	*	*	*	*	-	*	*	-	Good
Gregg, 2003	-	*	*	-	*	-	*	*	*	Fair
Hassan, 2023	*	*	*	*	*	-	*	*	-	Good
Hein, 1994	*	*	-	-	-	-	*	*	-	Poor
Higueras-Fresnillo, 2017	*	*	-	-	*	-	*	*	*	Fair
Holme, 2015	-	*	*	-	-	-	*	*	*	Poor
Hsu, 2018	*	*	*	*	*	-	*	*	-	Good
Huang, 2021	-	*	*	*	-	-	*	*	-	Poor
Hulsegge, 2016	-	*	*	*	*	-	*	*	*	Good
Jasiukaitiene, 2021	-	*	*	-	*	-	*	*	-	Fair
Johansson, 1999	*	*	-	-	*	-	*	*	*	Fair
Karvinen, 2015	-	*	*	-	*	-	*	*	-	Fair
Keadle, 2015	-	*	-	*	*	-	*	*	-	Fair
Kieffer, 2019	-	*	*	*	*	-	*	*	-	Good
Laddu, 2018	-	*	*	-	*	-	*	*	*	Fair
Lee, 2014	-	*	*	*	-	-	*	*	-	Poor

Lee, 2022	*	*	*	-	*	-	*	*	-	Good
Lee, 2023	-	*	*	-	*	-	-	*	-	Poor
Lewis, 2018	*	*	*	-	*	-	*	*	*	Good
Li, 2022	*	*	-	*	*	-	*	*	-	Good
Lissner, 1996	*	*	-	*	*	-	*	*	*	Good
Moholdt, 2020	*	*	*	*	*	-	*	*	-	Good
Mok, 2019	-	*	*	*	*	-	*	*	*	Good
Nauman, 2021	-	*	*	*	*	-	*	*	-	Good
Nordstoga, 2019	*	*	*	-	*	-	*	*	-	Good
Paffenbarger, 1994	-	*	*	-	*	-	*	*	*	Fair
Petersen, 2012	*	*	*	-	*	-	*	*	*	Good
Saint-Maurice, 2019	*	*	-	*	*	-	*	*	-	Good
Sanchez-Sanchez, 2020	*	*	*	-	*	-	*	*	-	Good
Schnohr, 2003	*	*	*	-	*	-	*	*	-	Good
Schnohr, 2017	*	*	*	*	-	-	*	*	-	Poor
Shaw, 2014	*	*	-	*	-	-	*	*	*	Poor
Shortreed, 2013	*	*	-	-	*	*	*	*	*	Fair
Stamatakis, 2024	-	*	*	*	*	-	*	*	-	Good
Trolle-Lagerros, 2005	*	*	-	*	*	-	*	*	-	Good
Vaes, 2014	*	*	*	-	*	-	-	*	-	Poor
Wannamethee, 1998	-	*	*	-	*	-	*	-	*	Fair
Williamson, 2019	*	*	*	-	*	*	*	*	-	Good
Wolin, 2010	-	*	-	*	-	-	*	*	*	Poor
Xue, 2012	*	*	*	-	-	-	*	*	*	Poor
Yang, 2021	*	*	*	-	*	*	*	*	*	Good
Yin, 2023	*	*	-	*	*	-	*	-	*	Good
Østergaard, 2018	-	*	*	-	-	-	*	*	-	Poor
Hamer, 2014	-	*	*	-	*	-	*	*	-	Good
Joseph, 2019	*	*	-	*	*	-	*	*	*	Good
Kaplan, 1996	*	*	-	*	*	-	*	*	*	Good
Lantz, 2010	*	*	-	-	*	-	*	*	-	Poor
Lee, 2003	-	*	*	-	-	-	*	*	-	Poor
Lee, 2004	-	*	*	-	-	-	*	*	*	Poor
O'Donovan, 2024	*	*	*	*	-	-	*	*	*	Poor
Opdal, 2021	*	*	-	-	*	-	*	*	-	Fair
Patterson, 2020	*	*	-	*	-	-	*	*	-	Poor
Reinikainen, 2015	*	*	-	-	-	-	*	*	-	Poor

Sabia, 2012	-	*	*	-	-	-	*	*	*	Poor
Sheehy, 2020	*	*	-	-	*	-	*	*	-	Fair
Stessman, 2009	*	*	-	*	*	-	*	*	*	Good
Talbot, 2007	-	*	-	*	*	-	*	*	-	Good
Bembom, 2009	-	*	*	-	*	-	*	*	-	Fair
Emberson, 2005	*	*	-	-	-	-	*	*	*	Poor
Fontana, 2024	-	*	*	-	-	-	*	*	-	Poor
Holtermann, 2012	*	*	-	-	*	-	*	*	-	Fair
Hu, 2004	-	*	*	-	*	-	*	*	*	Fair
Huerta, 2016	-	*	*	*	*	-	*	*	-	Good
Lee, 2022	-	*	*	*	*	-	*	*	*	Good
Martinez-Gomez, 2022	*	*	*	*	*	-	*	*	*	Good
Orsini, 2009	*	*	*	-	*	-	*	*	*	Good
Stenholm, 2016	*	*	-	-	*	-	*	*	-	Fair
Hirvensalo, 2000	*	*	*	*	-	-	*	*	-	Poor
Sherman, 1999	*	*	*	-	*	-	*	*	-	Good

Supplementary Table 3. Basic characteristics of included studies.

Publication (first author, year)	Study; country; analytical sample size (N)	Gender and baseline age	PA assessed time points and follow-up period for mortality	Physical activity domain and assessments	Methods of mortality ascertainment	Physical activity categories	Analytical methods	Covariates adjusted	Health outcomes, N(deaths), and risk estimates (95%CI)
**Trajectory PA**									
Aggio, 2020[29]	British Regional Heart Study; UK; N= 3,231	0% women; 40-59 years	(20-year PA trajectory)<br><br>PA change between 1978-1980 and 12-year, 16-year, 20-year follow-ups; median follow-up of 16.4 years from the last follow-up	Total PA measured by questionnaires; assessed by summary scores (0-5) based on intensity and frequency	National Health Service central registers	Inactive (0), occasional (1), light (2), moderate (3), moderately vigorous (4), vigorous (5)	Group-based trajectory modelling (GBTM) and cox regression	Age, occupational class, marital status, alcohol consumption, smoking status, region, previous diagnosis of MI, stroke or diabetes, LDL, HDL, systolic blood pressure, insulin, waist circumference and FEV1.<br><br>(full model further included markers IL-6, vWf,, Hs-TnT and NT-proBNP; to reduce heterogeneity, model 2 was selected)	**All-cause mortality (N=1735):**<br>Low-decreasing (ref)<br>Light-stable (0.78, 0.69-0.87)<br>Moderate-increasing (0.71, 0.61-0.81)<br><br>**CVD mortality (N=610):**<br>low-decreasing (ref)<br>Light-stable (0.67, 0.56-0.81)<br>Moderate-increasing (0.58, 0.45-0.74)
Ahmadi, 2022[30]	UK Biobank study; UK; N= 29,610	52.8% women; 40-69 years	(6-year PA change)<br><br>PA change from 2006-2010 to 2012-2018; mean follow-up of 5.1±2.1 years	Total MVPA measured by questionnaire; assessed in MET-min per week	Death data linkage	Inactive (0 MET-min/wk), Insufficient (>0 to <600 MET-min/wk), sufficient (≥600 MET-min/wk)	Cox regression	Age, sex, physical activity baseline and change group (or BMI baseline and change group for physical activity as an exposure); smoking status, alcohol consumption, ethnicity, sleep pattern, education, fruits and vegetables consumption, deprivation index, and cancer diagnosis	**All-cause mortality (N=545):**<br>Inactive stable (1.53, 1.12-1.92)<br>Inactive increased (0.71, 0.47-1.09)<br>Insufficient decreased (1.39, 1.05-1.87)<br>Insufficient stable (ref)<br>Insufficient increased (0.64, 0.49-0.85)<br>Sufficient decreased (1.00, 0.78-1.29)<br>Sufficient stable (0.74, 0.60-0.92)
Ahmadi, 2022[31]	Taiwan MJ cohort; China; N= 116,228	46.2% women; ≥18 years	(4.6-year PA change)<br><br>PA change between 1998-2013 and repeated measures 4.6 years apart; followed up for mortality for 11.9 years	Total PA measured by questionnaire; assessed in MET.h/week	National Death file	inactive (<1 MET-h): insufficient (> 1 to <7.5 MET-h); and sufficient (≥7.5 MET-h)	Cox regression	Age, sex, BMI group, smoking status, alcohol consumption, sleep duration, diet (fruits and vegetables consumption), and education	**All-cause mortality (N=3838):**<br>Inactive stable (1.46, 1.28-1.67)<br>Inactive increased (0.85, 0.76-0.95)<br>Insufficient decreased (1.39, 1.18-1.65)<br>Insufficient stable (ref)<br>Insufficient increased (0.85, 0.74-0.96)<br>Sufficient decreased (1.12, 0.97-1.24)<br>Sufficient stable (0.80, 0.71-0.92)<br><br>**CVD mortality (N=607):**<br>Inactive stable (1.63, 1.17-2.31)<br>Inactive increased (0.78, 0.63-0.94)<br>Insufficient decreased (1.77, 1.21-2.59)<br>Insufficient stable (ref)<br>Insufficient increased (0.72, 0.55-0.93)<br>Sufficient decreased (1.17, 0.90-1.46)<br>Sufficient stable (0.71, 0.58-0.88)<br><br>**Cancer mortality (N=1777):**<br>Inactive stable (1.37, 1.15-1.65)<br>Inactive increased (0.87, 0.75-1.02)<br>Insufficient decreased (1.33, 1.08-1.66)<br>Insufficient stable (ref)<br>Insufficient increased (0.79, 0.65-0.96)<br>Sufficient decreased (1.02, 0.85-1.21)<br>Sufficient stable (0.79, 0.64-0.95)
Aijo, 2016[32]	Evergreen Project; Finland; N=357	44.3% women; 75+ years	(5-year PA change)<br><br>PA measured in 1989-90 and 1994-95; 18-year follow-up for mortality	Total PA measured by questionnaires; assessed in intensity, frequency, and duration	Official archives and archives of hospitals and homes for the elderly	Inactive (score 1,2); active (score 3,4,5,6)<br><br>1 = mainly sitting;<br>2 = light PAs;<br>3 = moderate PA of about 3 h/week;<br>4 = moderate PA over 4 h/week or intense PA up to 4 h/week;<br>5 = active sports at least 3 h/week; 6 = strenuous exercise several times a week	Cox regression	Age, gender, stroke, diabetes, cardiac diseases, other diseases, and walking speed over 10 m/s	**All-cause mortality (N=347):**<br>Remained inactive (ref)<br>Change to active (1.02, 0.51-2.03)<br>Change to inactive (1.34, 0.77-2.35)<br>Remained active (0.98, 0.62-1.56)
Andersen, 2004[33]	Pooled data from the Copenhagen City Heart Study, Glostrup Population	49% women; 20-93 years	(6.7-year PA change)<br><br>PA change between 2 time points starting in	Leisure-time PA measured by questionnaire	Danish Central Population Registry	Sedentary, moderately active, and highly active	Cox regression	Age and sex (adjusted for total cholesterol, SBP, education, smoking and BMI did not change the results)	**All-cause mortality (N=932):**<br>Remained sedentary (ref)<br>Inactive to moderately active (0.74, 0.61-0.89)<br>Inactive to highly active (0.74, 0.58-0.95)

	Studies and Copenhagen Male Study; Denmark; N= 14,820		1964, average 6.7 years apart; Mean 10.8 years (women) and 9.6 years (men) from the second time point						
Balboa-Castillo, 2011[34]	Cohort study of the non-institutionalized Spanish population; Spain; N= 2,732	57% women; ≥60 years	(2-year PA change)<br><br>PA change from 2001 to 2003; follow-up of 6 years from 2003	Leisure-time PA measured by questionnaire; assessed in PA frequency	National Death Index	Sedentary (no exercise), active (occasional or regular exercise)	Cox regression	Sex, age, education, smoking, alcohol, CHD, stroke, cancer, COPD, diabetes, hip fracture, SF36 physical summary, Mini-Mental State Examination, BMI, waist circumference, limitations in mobility, agility and in instrumental ADLs	**All-cause mortality (N=505):**<br>Remained sedentary (ref)<br>Sedentary to active (0.66, 0.52-0.84)<br>Active to sedentary (0.93, 0.70-1.24)<br>Remained active (0.55, 0.43-0.70)
Barbiellini, 2022[35]	Progetto Veneto Anziani (Pro.V.A.) cohort study; Italy; N= 2,754	60.2% women; aged ≥65 years	(7-year PA change)<br><br>PA change between 1995-1997 and follow-up visits after 4 and 7 years; followed up to 2018	Total MVPA measured by questionnaires; assessed in min/day	Hospital discharge records and mortality records	Inactive (≤20 min/day), active (>20 min/day)	Cox regression	Sex, education and number of members living in the same household, smoking and alcohol consumption, cancer, chronic obstructive pulmonary disease, asthma, hypoacusia, hypovisus, diabetes, chronic kidney disease, anaemia, Parkinson's disease, dyslipidaemia, osteoporosis with or without femur fracture, congenital mental retardation, osteoarthrosis, discopathy, orthostatic hypotension, urinary incontinence and bowel incontinence, CHD, heart failure, stroke, angina, transient ischaemic attack, atrial fibrillation, hypertension and peripheral artery disease, with the exclusion of the outcome of interest at baseline, and stratified by categorized year of birth.	**All-cause mortality (N=1355):**<br>Men:<br>Low-stable (ref)<br>High-decreasing (1.04, 0.72-1.50)<br>Low-increasing (0.75, 0.50-1.11)<br>High-stable (0.60, 0.43-0.83)<br><br>Women:<br>Low-stable (ref)<br>High-decreasing (1.06, 0.85-1.32)<br>Low-increasing (0.91, 0.72-1.14)<br>High-stable (0.81, 0.67-0.98)
Bauman, 2017[36]	Danish MONICA (MONItoring Trends and Determinants in Cardiovascular Disease); Denmark; N= 2,412	49% women; 30-61 years	(11-year PA pattern)<br><br>PA change within 5 or 11-year pattern (1983-1988 or 1983-1994); followed up until 2007	Leisure-time PA measured by questionnaires; assessed in weekly hours and intensity of exercise	National Registers of Hospital Discharge and Death Registry	Inactive (no exercise), moderately active (<4h moderate PA), highly active (≥4h moderate PA)	Cox regression	Age, sex and education	**All-cause mortality (11-year LTPA) (N=445):**<br>Remained inactive (ref)<br>Remained active (0.41, 0.28-0.59)<br>Mixed pattern (0.66, 0.50-0.87)<br><br>**CVD mortality (11-year LTPA) (N=185):**<br>Remained inactive (ref)<br>Remained active (0.61, 0.33-1.15)<br>Mixed pattern (1.08, 0.66-1.77)
Bergwall, 2021[37]	Malmö Diet and Cancer Study cohort; Sweden; N= 18,555	62% women; 44-74 years	(5-year PA change)<br><br>PA change between 1991–1996 and after 5 years; a mean of 20 years of follow-up	A range of leisure-time activities measured by questionnaire; assessed by participation	Civic registration	Not participated; participated	Cox regression	Age, sex, screening date, education, smoking status, alcohol, diet index, total energy intake and BMI	**CVD mortality (N=1514):**<br>Cycling:<br>Never (ref)<br>Stopped (0.98, 0.85-1.14)<br>Started (0.81, 0.65-1.02)<br>Continued (0.82, 0.73-0.92)
Bijnen, 1999[38]	The Zutphen Elderly Study; the Netherlands; N=472	0% women; 65–85 years	(5-year PA change)<br><br>PA measured between 1985 and 1990; 5-year follow-up from 1990	Total PA assessed via questionnaire; weekly duration and frequency assessed for different activities	Municipal registries	Active: walked or cycled for 20 minutes at least three times per week<br>Inactive: below the active PA level	Cox regression	Age, prevalence of disease (CVD, cancer, chronic non-specific lung diseases, diabetes mellitus), functional status, cigarette smoking and alcohol intake in 1990	**All-cause mortality (N=118):**<br>Remained inactive (ref)<br>Inactive-active (0.68, 0.32-1.45)<br>Active-inactive (0.86, 0.41-1.77)<br>Active-active (0.50, 0.29-0.84)
Breidablik, 2023[39]	HUNT Study; Norway; N= 123,005	52% women; 19-104 years	(33-year PA pattern)<br><br>PA change between HUNT1 (1984-1986) and HUNT4 (2017-2019); mortality followed up to 2020	Leisure-time PA measured by questionnaires; assessed in weekly frequency, intensity and duration	Death certificates	Inactive (<60 or <150 minutes per week), active	Cox regression	Sex, education, BMI, smoking, long-term somatic disease, long-term mental disease, and self-rated health	**All-cause mortality (N=N.A.):**<br>60-min threshold:<br>Persistently inactive pattern (ref)<br>Mixed PA pattern (0.86, 0.73-1.01)<br>Persistently active (0.57, 0.45-0.72)<br><br>150-min threshold:<br>Persistently inactive pattern (ref)<br>Mixed PA pattern (0.86, 0.77-0.97)<br>Persistently active (0.53, 0.29-0.97)
Byberg, 2009[40]	Uppsala Longitudinal Study of Adult Men; Sweden; N= 1,759	0% women; 49-51 years	(10-year PA change)<br><br>PA changes between age 50 and 60; follow-up of 22 to 26 years from age 60	Leisure-time PA measured by questionnaire	Swedish National Population Register	low (mostly sedentary); medium (often walking/cycling for pleasure); high (active sport at least 3 hours per week or hard physical training)	Cox regression	Smoking, obesity, height and weight, self-perceived health, PA at work, diabetes, any musculoskeletal, neurological or psychiatric disorders and alcohol use	**All-cause mortality (N=998):**<br>Unchanged high (ref)<br>Increased from low/medium to high (1.21, 0.99-1.48)<br>Reduced from high to low/medium (1.35, 1.12-1.61)<br>Unchanged low/medium (1.33, 1.12-1.56)
Cheema, 2023[41]	National Health and Nutrition Examination Survey (NHANES) & National Health	55.8% women; 25-74 years	(11-year PA change)<br><br>PA measured between 1971-75 and 1982-84; mean 17.6-year follow-	Recreational PA measured by questionnaires	National Death Index	Inactive ("little or no" response), active ("much/moderate" response)	Competing risk Cox regression	Age, sex, race/colour, income, education, smoking, alcohol drinking, change in non-recreational activity from baseline to first follow-up, BMI	**CVD mortality (N=404):**<br>Stable active (ref)<br>Decreasing (1.50, 1.03-2.18)<br>Increasing (1.25, 0.97-1.61)<br>Stable inactive (1.58, 1.10-2.27).

	Epidemiologic Follow-up Study (NHEFS); USA; N= 4,921		up					
Choi, 2022[42]	National Health Insurance Service-National Sample Cohort; Korea; N= 286,402	46.7 % women; ≥20 years	(1.6-year PA change)<br><br>PA changes from 2009-2011 to 2010-2012; median follow-up of 4.3 years for mortality	Total MVPA measured by self-reported questionnaires; assessed in weekly MVPA frequency	Death registration database of Statistics Korea	(1) physical inactivity, (2) one to two MVPA sessions per week, (3) three to four MVPA sessions per week, and (4) more than five MVPA sessions per week	Cox regression	Sex, age, BMI, SBP, DBP, serum glucose, total cholesterol, alcohol consumption, cigarette smoking status, household income, location of residence, disability, and comorbidities	**All-cause mortality (N=4494):**<br>Remained inactive (ref)<br>Increased to 1-2 sessions per week (0.82, 0.73-0.92)<br>Increased to 3-4 sessions per week (0.72, 0.62-0.84)<br>Increased to 5+ sessions per week (0.73, 0.63-0.85)<br><br>**CVD mortality (N=820):**<br>Remained inactive (ref)<br>Increased to 1-2 sessions per week (0.68, 0.51-0.91)<br>Increased to 3-4 sessions per week (0.48, 0.31-0.74)<br>Increased to 5+ sessions per week (0.70, 0.50-0.98)
Coelho-Ravagnani, 2021[43]	Aerobics Center Longitudinal Study; USA; N= 15,441	0% women; 18–100 years	PA change from 1970 to 2002, at least 2 examinations; mean follow-up of 6.2 years from last examination	Leisure-time PA measured by questionnaires; assessed in MET.min/week	National Death Index	Inactive (<500 MET.min/week), active (≥500 MET.min/week)	Cox regression	Age, baseline BMI, change in smoking and drinking status, change in hypertension, diabetes, and hypercholesterolemia status, parental history of CVD and BMI change	**CVD mortality (N=439):**<br>Remained inactive (ref)<br>Became active (1.07, 0.85-1.35)<br>Became inactive (0.93, 0.60-1.43)<br>Remained active (0.53, 0.36-0.77).
Duarte Junior, 2024[44]	Seniors-ENRICA 1 and 2; Spain; N= 3,963	53% women; 60-96 years	(2.89-year PA change)<br><br>PA measured between baseline and wave 1, mean 2.89 years apart; mean 6.4-year follow-up for mortality	Leisure-time activities measured by questionnaires; calculated as the sum of the time spent in various PA types	National Death Index	Very low (<7 h/week); low, middle, and high calculated based on tertiles of those with ≥ 7h/week	Cox regression	Sex, age, educational level, smoking status, alcohol status, BMI status, cardiovascular disease, cancer, diabetes mellitus, hypertension, cohort, and sum of change over time in all other physical activities	**All-cause mortality (N=1063):**<br>Remained inactive (ref)<br>Decreased (0.64, 0.45-0.92)<br>Increased (0.43, 0.29-0.62)<br>Remained active (0.47, 0.32-0.67)
Dwyer, 2015[45]	Australian Diabetes, Obesity and Lifestyle (AUSDIAB) study; The Tasmanian Older Adult Cohort (TASOAC) study; The Tasmanian Study of Cognition and Gait (TASCOG) study; Australia; N=1,679	51.6% women; mean age 58.8 years	(3.7-year PA change)<br><br>Daily step activity change from baseline to 3.7 years later; followed up to more than 10 years to 2011	Daily steps measured by pedometer	Australian National Death Index	No information	Cox regression	Age, sex, BMI, total energy intake, smoking, alcohol intake and education at baseline, study cohort, change in BMI, and baseline daily steps.	**All-cause mortality (N=219):**<br>Compared with no increase or a decrease in daily steps, any increase in daily steps had risk ratio 0.38 (0.21-0.70).
Gregg, 2003[46]	Study of Osteoporotic Fractures (SOF); USA; N= 7,553	100% women; ≥65 years	(mean 5.7-year PA change)<br><br>PA changes between 1986-1988 and 1992-1994; Up to 6.7 years from last follow-up	Leisure-time PA measured by questionnaire; assessed in kilocalories per week	Death certificates	Inactive (<595 kcal/week), active (≥ 595 kcal/week)	Cox regression	Age, smoking, BMI, stroke, diabetes, hypertension, self-rated health status, CHD, cancer, COPD, incident hip fracture and baseline PA	**All-cause mortality (N=1029):**<br>Remained inactive (ref)<br>Inactive to active (0.52, 0.40-0.69)<br>Active to inactive (0.92, 0.77-1.09)<br>Remained active (0.68, 0.56-0.82)<br><br>**CVD mortality (N=386):**<br>Remained inactive (ref)<br>Inactive to active (0.64, 0.42-0.97)<br>Active to inactive (1.07, 0.81-1.42)<br>Remained active (0.62, 0.44-0.88)<br><br>**Cancer mortality (N=264):**<br>Remained inactive (ref)<br>Inactive to active (0.49, 0.29-0.84)<br>Active to inactive (0.61, 0.42-0.90)<br>Remained active (0.82, 0.58-1.16)
Hassan, 2023[47]	Cardiovascular Disease, Living and Ageing in Halle study (CARLA); Germany; N= 1,041	45.6% women; 45-83 years	(mean 8.8-year PA change)<br><br>PA change between 2002-2006, 2007-2010 and 2013; 6.2 years follow-up since 2013	Leisure-time PA and sports PA measured by questionnaires; assessed in summary score based on frequency (2-10)	Official death certificate	Low (mean 4.8), moderate (mean 6.2), high (mean 7.4)	Growth mixture modelling and Cox regression	Age, sex, smoking, alcohol, BMI, education, MI, stroke, cancer, lipid-lowering drugs	**All-cause mortality (N=121):**<br>Consistently low PA (ref)<br>Consistent moderate PA (0.47, 0.30–0.70)<br>High PA-levels at baseline but strongly decreasing PA across time (0.83, 0.50–1.40)
Hein, 1994[48]	The Copenhagen	0% women;	(15-year PA change)	Leisure-time PA	The National	Inactive <4h/week	Logistic regression	Analysis stratified by age (53–63 years and 64–75 years)	**All-cause mortality (N=270):**

	Male Study; Denmark; N=2894	40–59 years	PA measured between 1970–71 and 1985–86; 6-year follow-up from 1985–86	assessed via questionnaire; weekly duration and frequency assessed for different activities	Health Service register and from the Danish Institute of Clinical Epidemiology	Active ≥4h/week			53-63 years:<br>Remain <4h/week (ref)<br>Become more active (≥4h/week) (1.10, 0.40-3.50)<br><br>64-75 years:<br>Remain <4h/week (ref)<br>Become more active (≥4h/week) (1.00, 0.50-1.90)
Higueras-Fresnillo, 2017[49]	Universidad Autónoma de Madrid (UAM) cohort; Spain; N= 2,836	56.7% women; ≥60 years	(2-to3-year PA change)<br><br>PA change from 2000-2001 to 2003; mean follow-up 9 years from 2003 to 2014	Leisure-time PA measured by questionnaires; assessed via self-reported frequency	Spanish National Institute of Statistics database	Inactive; occasional; several times a month/several times a week	Cox regression	Age, sex, education, smoking, alcohol, subjective health, Mini-Mental State examination, BMI, SBP, hypercholesterolemia, agility disability, mobility disability, limitation in instrumental ADLs and the following self-reported diseases diagnosed: asthma/bronchitis, CHD, stroke, diabetes, depression, hip fracture and cancer	**CVD mortality (N=467):**<br>Consistently inactive (ref)<br>Increased to occasional/regular (0.75, 0.57-0.99)<br>Decreased to inactive (0.96, 0.68-1.34)<br>Remained occasional/regular (0.42, 0.31-0.58)
Holme, 2015[50]	Oslo study; Norway; N= 5,738	0% women; 40-49 years	(28-year PA change)<br><br>PA changes between 1972-1973 and 2000; follow-up of 12 years from last examination	Leisure-time PA measured by questionnaire; assessed in weekly duration	Death certificates	Inactive (sedentary); active (light /moderate /hard)	Cox regression	Age, education, smoking, diabetes, previous MI and previous stroke	**All-cause mortality (N=2154):**<br>Remained sedentary (ref),<br>Increased PA 0.56 (0.43-0.74)
Hsu, 2018[51]	Concord Health and Aging in Men Project (CHAMP); Australia; N= 1,705	0% women; ≥70 years	(5-year PA change)<br><br>PA change between 2005-2007 and 2 years and 5 years later; median follow-up of 7 years	Various exercise activities measured by questionnaires; assessed in PASE score based on weekly frequency, duration and intensity	New South Wales Registry of Births, Deaths, and Marriages	No information	Poisson regression with generalized estimating equations (GEE)	Age, comorbidity, smoking status, alcohol, BMI, ethnicity, education, cardiovascular disease, diabetes, self-rated health, ADL disability, depression and PASE score (based on walking, muscle and sport exercise)	**All-cause mortality (N=519):**<br>No exercise (ref)<br>Maintained or increased walking (0.95, 0.70-1.29)<br>Maintained or increased muscle strengthening exercise (0.72, 0.49-1.06)<br>Maintained or increased light sport (0.93, 0.64-1.36)<br>Maintained or increased moderate sport (0.75, 0.44-1.27)<br>Maintained or increased strenuous sport (0.65, 0.42-0.99)<br><br>**CVD mortality (N=185):**<br>No exercise (ref)<br>Maintained or increased walking (0.95, 0.58-1.56)<br>Maintained or increased muscle strengthening exercise (0.53, 0.27-1.04)<br>Maintained or increased light sport (0.64, 0.33-1.23)<br>Maintained or increased moderate sport (0.65, 0.26-1.68)<br>Maintained or increased strenuous sport (0.49, 0.23-1.03)<br><br>**Cancer mortality (N=151):**<br>No exercise (ref)<br>Maintained or increased walking (0.90, 0.55-1.46)<br>Maintained or increased muscle strengthening exercise (0.66, 0.33-1.29)<br>Maintained or increased light sport (1.23, 0.64-2.34)<br>Maintained or increased moderate sport (0.91, 0.42-2.00)<br>Maintained or increased strenuous sport (0.44, 0.20-0.95)
Huang, 2021[52]	Guangzhou Biobank Cohort Study; China; N= 18,104	73% women; ≥50 years	(5-year PA change)<br><br>PA change from 2003-2008 to 2008-2012; mean follow-up of 7.8 years from 2008-2012	Total PA measured by questionnaire; assessed in MET.min/week	Death Registry of the Guangzhou Centre for Disease Control and Prevention	Low (<480 MET.min/week vigorous PA), moderate (480 MET.min/week to 1500 MET.min/week vigorous PA), and high (>1500 MET.min/week vigorous PA)	Cox regression	Sex, age, occupation, personal income, education, alcohol use, smoking and self-rated health	**All-cause mortality (N=1461):**<br>Low-low (2.08, 0.51–8.40)<br>Low-moderate (0.88, 0.59–1.33)<br>Low-high (0.71, 0.52–0.97)<br>Moderate-low (1.54, 1.05–2.26)<br>Moderate-moderate (ref)<br>Moderate-high (0.90, 0.76–1.07)<br>High-low (1.41, 0.97–2.06)<br>High-moderate (0.93, 0.76–1.15)<br>High-high (0.83, 0.70–0.98)<br><br>**CVD mortality (N=703):**<br>Low-low (6.53, 1.58–26.91)<br>Low-moderate (0.87, 0.43–1.73)<br>Low-high (0.76, 0.45–1.28)

									Moderate-low (2.19, 1.28–3.78)<br>Moderate-moderate (ref)<br>Moderate-high (0.83, 0.62–1.11)<br>High-low (1.26, 0.67–2.39)<br>High-moderate (0.97, 0.69–1.36)<br>High-high (0.82, 0.62–1.08)
Hulsegge, 2016[53]	Doetinchem Cohort Study; The Netherlands; N= 5,263	54% women; 26-66 years	(5-year PA change)<br><br>PA changes between 1993–1997 and 1998–2002; mean follow-up of 12.2 years	Total PA measured by questionnaire; assessed in hours/week	Municipal population register; Statistics Netherlands	Not healthy (<3.5h/week), healthy (≥3.5h/week)	Cox regression	Age, sex, education, occupation, BMI, smoking, Mediterranean diet score and alcohol consumption	**All-cause mortality (N=338):**<br>Remained inactive (ref)<br>Changing from inactive to active (1.04, 0.63-1.71)
Jasiukaitiene, 2021[54]	Multinational Monitoring of Trends and Determinants in Cardiovascular Disease (MONICA) study; Lithuania; N= 2,416	56% women; 35-64 years	(mean 15.2-year PA change)<br><br>PA change between one of the initial surveys ((1986–1987, 1992–1993, 2001–2002) and 2006-2008; mean 10.6 years follow-up from 2006	Leisure-time PA measured by questionnaires; assessed in weekly duration	Regional mortality register	From lowest to highest and divided into three equal tertiles: physically inactive, moderate physically active, physically active	Cox regression	Baseline age, education and biological factors (i.e. systolic blood pressure, total cholesterol, triglycerides, fasting glucose, BMI) and smoking status	**All-cause mortality (N=382):**<br>Men:<br>Remained active (ref)<br>Increased PA level (0.85, 0.57-1.26)<br>Decreased PA level (1.03, 0.67-1.58)<br>Remained inactive (1.49, 0.99-2.26)<br><br>Women:<br>Remained active (ref)<br>Increased PA level (1.50, 0.96-2.33)<br>Decreased PA level (1.82, 1.03-3.21)<br>Remained inactive (1.67, 0.87-3.20)<br><br>**CVD mortality (N=67):**<br>Men:<br>Remained active (ref)<br>Increased PA level (0.69, 0.25-1.94)<br>Decreased PA level (0.93, 0.30-2.88)<br>Remained inactive (2.29, 0.85-6.18)<br><br>Women:<br>Remained active (ref)<br>Increased PA level (1.89, 0.56-6.45)<br>Decreased PA level (5.40, 1.41-20.7)<br>Remained inactive (3.01, 0.53-17.0)
Johansson, 1999[55]	Swedish Annual Level-of-Living Survey; Sweden; N=3843	51% women; 25–74 years	(8-year PA change)<br><br>PA measured between 1980–81 and 1988–89; 6-year follow-up from 1988–89	Leisure-time PA assessed via questionnaire; PA intensity and frequency assessed	Cause-of-Death Register	Being active: regular PA at least once a week	Cox regression	Sex, age, marital status, education, smoking, overweight, BMI and hypertension	**All-cause mortality (N=357):**<br>No-no (ref)<br>No-yes (0.74, 0.45-1.23)<br>Yes-no (0.93, 0.56-1.52)<br>Yes-yes (0.53, 0.39-0.74)
Karvinen, 2015[56]	Finish Twin Cohort; Finland; N= 4,190 pairs	55% women; 18-45 years	(15-year PA change)<br><br>PA change from 1975 to 1981 to 1990. follow-up of 23 years from 1990 to 2013	Leisure-time vigorous activity measured by questionnaires; assessed based on intensity categories	Population Register Centre of Finland	No vigorous activity (only walking); with vigorous activity (activities more intensive than normal walking, at least 3-5 times a month)	Cox regression	Sex, age, education, smoking status in 1990, alcohol consumption, BMI in 1990, work activity, Somatic Disease Index (self-reporting any doctor-diagnosed disease, or life event causing serious illness or injury, or self-reported permanent work disability) in 1990	**All-cause mortality (N=1478):**<br>No vigorous PA (ref)<br>Persistent vigorous PA (0.73, 0.61-0.88)<br>No-yes (0.86, 0.75-0.99)<br>Yes-no (0.95, 0.78-1.16)<br>Mixed PA (0.86, 0.72-1.03)
Keadle, 2015[57]	NIH-AARP Diet and Health Study; USA; N= 165,087	Men & women; 50-71 years	(10-year PA change)<br><br>PA measured in 1994-96 and 2004-06; follow-up of 6.6 years until 2011	Leisure-time MVPA measured by questionnaires; assessed in hours/week	Social Security Administration Death Master File and the National Death Index	MVPA <1, 1-4, and 4+ hours per week	Cox regression	Age, sex, race, education, smoking history, history of heart disease, other chronic conditions, depression, health status, television viewing and change in BMI category from Time 1 to time 2.	**All-cause mortality (N=20104):**<br>Remained <1 h/week (ref)<br><1 to 1-4 h/week (0.82, 0.77-0.87)<br><1 to 4+ h/week (0.67, 0.62-0.72).
Kieffer, 2019[58]	Nord-Trøndelag Health Study (HUNT); Norway; N= 24,880	52.3% women; ≥20 years	(11-year PA change)<br><br>Change in PAI scores from HUNT1 (1984-86) to HUNT2 (1995-97); median follow-up of 19.3 years	Leisure-time PA measured by self-reported questionnaires; PAI score calculated based on weekly PA duration, frequency and intensity	Norwegian cause of death registry	Low PAI (<100), high PAI (≥100)	Cox regression	Age, sex, education, alcohol, smoking status, diabetes, hypertension, BMI, total cholesterol, HDL cholesterol, triglycerides	**All-cause mortality (N=4782):**<br>Continually low PAI (ref)<br>Decreased PAI (0.92, 0.83-1.01)<br>Increased PAI (0.86, 0.79-0.95)<br>Continually high PAI (0.80, 0.71-0.91)<br><br>**CVD mortality (N=1560):**<br>Continually low PAI (ref)

									Decreased PAI (0.87, 0.73-1.04)<br>Increased PAI (0.87, 0.74-1.03)<br>Continually high PAI (0.68, 0.54-0.86)
Laddu, 2018[59]	Osteoporotic Fractures in Men (MrOS) Study; USA; N= 3,767	0% women; ≥65 years	(7-year PA trajectory)<br><br>PA change between up to four time points from 2000 to 2009; mean follow-up 7.1 years from 2009	Total PA measured by questionnaire; estimated from weekly intensity, frequency, and duration of various activities	Death certificates	Low, moderate high	Group-based trajectory modelling (GBTM) and cox regression	Age, race, clinic site, alcohol, depression, cognitive function, marital status, education, self-rated health, any one co-morbidity (COPD, stroke, MI, congestive heart failure, Parkinson's, diabetes), smoking and BMI	**All-cause mortality (N=1435):**<br>Low-declining (ref)<br>Moderate-declining (0.78, 0.70-0.88)<br>High-declining (0.69, 0.57-0.83)<br><br>**CVD mortality (N=484):**<br>Low-declining (ref)<br>Moderate-declining (0.81, 0.66-0.99)<br>High-declining (0.68, 0.49-0.95)<br><br>**Cancer mortality (N=343):**<br>Low-declining (ref)<br>Moderate-declining (0.79, 0.63-1.01)<br>High-declining (0.82, 0.55-1.19)
Lee, 2022[60]	National Health Insurance Service; Korea; N= 6,572,984	43.3% women; ≥20 years	(2-year PA change)<br><br>PA measured in 2009 and 2011; median 7.4-year follow-up	Total PA measured by questionnaires; assessed in frequency, duration, and intensity	NHIS database	Inactive (no regular exercise), active (regular exercise)	Cox regression	Age, sex, smoking, drinking, income, diabetes, hypertension, dyslipidaemia, and BMI; stratified by weight gain/loss	**All-cause mortality (N=91347):**<br>Remained active (ref)<br>Inactive to active (1.15, 1.11-1.20)<br>Active to inactive (1.24, 1.19-1.29)<br>Remained inactive (1.46, 1.41-1.50)
Lee, 2023[61]	Taiwan MJ cohort; China; N= 21,211	44.6% women; 18-90 years	(18-year PA change)<br><br>PA change from 1996 to 2014; median follow-up time 16.8 years to May 2021	Leisure-time PA measured by questionnaires; assessed in MET.h/week	No information	Low, moderate, high	Group-based trajectory modelling (GBTM) and cox regression	Age, sex, BMI, systolic blood pressure, smoking, history of cardiovascular disease, fasting plasma glucose, estimated glomerular filtration rate, and total cholesterol	**All-cause mortality (N=960):**<br>Low-stable (ref)<br>Medium-stable (0.84, 0.72–0.98)<br>Increasing (0.57, 0.33–0.97)<br>Decreasing (0.58, 0.27–1.22)<br>Fluctuating (0.99, 0.70–1.39)<br><br>**CVD mortality (N=177):**<br>Low-stable (ref)<br>Medium-stable (1.03, 0.72–1.48)<br>Increasing (0.82, 0.26–2.54)<br>Decreasing (1.48, 0.47–4.62)<br>Fluctuating (1.35, 0.68–2.67)<br><br>**Cancer mortality (N=399):**<br>Low-stable (ref)<br>Medium-stable (0.68, 0.52–0.89)<br>Increasing (0.71, 0.34–1.51)<br>Decreasing (0.40, 0.10–1.65)<br>Fluctuating (1.05, 0.62–1.77)
Lee, 2014[62]	Aerobics Center Longitudinal Study; USA; N= 20,647	26% women; 18-100 years	(5.9-year PA change)<br><br>PA changes between 1974 and 2002, mean interval of 5.9 years; mean follow-up of 14.7 years for all-cause mortality and 14.6 years for CVD mortality	Leisure-time running measured by questionnaire; assessed based on duration, distance, frequency, amount, and speed	National Death Index	Non-runners; runners	Cox regression	Baseline age (years), sex, examination year, interval between the baseline and last examinations (years), baseline smoking status, alcohol consumption, other physical activities except running, and parental cardiovascular disease	**All-cause mortality (N=1398):**<br>Non-runners (ref)<br>Became non-runners (0.79, 0.66-0.95)<br>Become runners (0.89, 0.74-1.07)<br>Remained runners (0.71, 0.59-0.87)<br><br>**CVD mortality (N=490):**<br>Non-runners (ref)<br>Became non-runners (0.66, 0.48-0.91)<br>Become runners (0.70, 0.50-0.99)<br>Remained runners (0.50, 0.34-0.74)
Lewis, 2018[63]	Hispanic Established Population for the Epidemiologic Study of the Elderly (H-EPESE); USA; N= 803	63% women; ≥67 years	(10-year PA change)<br><br>PA change from 1995-1996 to 2005-2006; follow-up of 7 years from 2005-2006	Total PA measured by questionnaires; assessed in PASE score based on frequency, duration and intensity	National Death Index	Low (<105.8), high (>105.8)	Cox regression	Age, gender, marital status, education, smoking, PA groups, BMI, depressive symptoms, cognition, functional disability and co-morbidities	**All-cause mortality (N=367):**<br>Unchanged low (ref)<br>Low-high (0.57, 0.34-0.97)<br>High-low (1.01, 0.76-1.34)<br>Unchanged high (0.77, 0.53-1.10)
Li, 2022[64]	English Longitudinal Study of Ageing (ELSA); England; N= 8,842	56.2% women; ≥50 years	(6-year PA change)<br><br>PA change between 2002-03 and 2008-09; followed up for 4 years	Leisure-time PA measured by questionnaires; assessed via frequency of mild,	Interview and data linkage	Low; moderate; high (derived from trajectory of weighted Z score)	Group-based trajectory modelling (GBTM) and cox regression	age, sex, ethnicity, education, cohabitation status, current smoking, alcohol consumption, depressive symptoms, hypertension, diabetes, stroke, cardiovascular diseases, chronic lung diseases, cancer, overweight status and cognitive status.	**All-cause mortality (N=163):**<br>Persistently low (ref)<br>Initially low then improving (0.31, 0.13-0.74)<br>Initially high then declining (0.50, 0.24-1.05)<br>Persistently moderate (0.55, 0.38-0.79)

			from 2008-09	moderate and vigorous PA					Persistently high (0.25, 0.14-0.45)
Lissner, 1996[65]	The Gothenburg Prospective Study of Women; Sweden; N= 1267	100% women; 38–60 years	(6-year PA change)<br><br>PA measured between 1968-69 and 1974-75; 14-year follow-up from 1974-75	Leisure-time PA assessed via questionnaire; assessed in intensity and duration	Swedish National Death Registry and the State Person and Address Registry	Activity group 1: completely inactive<br>Activity group 2: some PA ≥4h/week<br>Activity group 3: regular PA<br>Activity group 4: regular training/competition	Cox regression	Adulthood activity index, initial age, baseline levels of and 6-year changes in smoking, serum triglycerides, BMI, waist–hip ratio, and BP	**All-cause mortality (N=110):**<br>No change (ref)<br>Increased (1.11, 0.67-1.86)<br>Decreased (2.07, 1.39-3.09)
Moholdt, 2020[66]	HUNT Study; Norway; N= 32,811 in HUNT1 and 2	51 to 54% women; ≥20 years	(11-year PA change)<br><br>PA change between: HUNT1 and 2; follow-up of 18 years from HUNT2	Leisure-time PA measured by questionnaires; assessed in frequency, duration and intensity	National Cause of Death Registry	Inactive; below recommended; recommended (moderate PA to ≥150 min/week and vigorous PA to ≥60 min/week)	Cox regression	Age, sex, BMI, smoking, education, occupational physical activity, diabetes, systolic blood pressure	(PA change from HUNT1(1984-86) to HUNT2(1995-97))<br><br>**All-cause mortality (N=7749):**<br>Above-above (ref)<br>Above-below (1.24, 1.09-1.40)<br>Above-inactive (1.51, 1.33-1.71)<br>Below-above (1.04, 0.91-1.19)<br>Below-below (1.31, 1.16-1.47)<br>Below-inactive (1.41, 1.26-1.58)<br>Inactive-above (1.26, 1.10-1.44)<br>Inactive-below (1.32, 1.18-1.48)<br>Inactive-inactive (1.56, 1.40-1.73)<br><br>**CVD mortality (N=3064):**<br>Above-above (ref)<br>Above-below (1.41, 1.14-1.74)<br>Above-inactive (1.80, 1.46-2.21)<br>Below-above (1.11, 0.88-1.40)<br>Below-below (1.61, 1.32-1.96)<br>Below-inactive (1.65, 1.37-1.99)<br>Inactive-above (1.45, 1.16-1.83)<br>Inactive-below (1.56, 1.28-1.90)<br>Inactive-inactive (1.94, 1.62-2.32)
Mok, 2019[67]	EPIC-Norfolk cohort; UK; N= 14,599	57% women; 40-79 years	(7.6-year PA trajectory)<br><br>PA change from 1993-1997 to 7.6 years later; Median 12.5 years from the last follow-up	Total PA measured by questionnaires; assessed in physical activity energy expenditure (PAEE)	Death certificates	low (PAEE=0 kJ/kg/day), medium (0<PAEE<8.4 kJ/kg/day), high (PAEE≥8.4 kJ/kg/day)	Cox regression	Age, sex, smoking, education, social class, self-rated health, alcohol intake, energy intake, diet quality, co-morbidities (CVD, cancer, diabetes, asthma, COPD and bone fractures), BMI, BP, triglycerides, LDL, HDL cholesterol and time-updated for lifestyle and health-related variables at second visit	**All-cause mortality (N=3148):**<br>Low-maintainers (ref)<br>Low-increaser (0.76, 0.65-0.88)<br>Medium-decreaser (0.90, 0.81-1.00)<br>Medium-maintainer (0.72, 0.62-0.82)<br>Medium-increaser (0.62, 0.53-0.72)<br>High-decreaser (0.80, 0.71-0.91)<br>High-maintainer (0.67, 0.53-0.84)<br>high-increaser (0.58, 0.43-0.78)<br><br>**CVD mortality (N=950):**<br>For each 1 kJ/kg/day per year increase in PAEE 0.71 (0.62-0.82).<br><br>**Cancer mortality (N=1091):**<br>For each 1 kJ/kg/day per year increase in PAEE 0.89 (0.79-1.00).
Nauman, 2021[68]	Aerobics Center Longitudinal Study (ACLS); USA; N= 17,613	18.4% women; 20-82 years	(mean 6.3-year PA change)<br><br>Change in PAI between 1974 and 2002; median follow-up time of 9.3 years	Leisure-time PA measured by self-reported questionnaires; PAI score calculated based on weekly PA duration, frequency and intensity	National Death Index	Inactive (0 PAI), not active enough (0-99 PAI), active enough (≥100 PAI)	Cox regression	Age, number of clinical visits and number of years between the first and last examinations, parental cardiovascular disease, combination pattern of changes in confounders (BMI, smoking status, hypertension, diabetes, and hypercholesterolemia) between the first and last examinations, PAI score at first examination, and stratified by sex	**All-cause mortality (N=1144):**<br>Remained inactive (ref)<br>Remained active enough (0.58, 0.41-0.83)<br>Increase from inactive to active enough (0.82, 0.69-0.99)<br>Decrease from active enough to inactive (0.73, 0.50-1.07)<br><br>**CVD mortality (N=400):**<br>Remained inactive (ref)<br>Remained active enough (0.49, 0.26-0.95)<br>Increase from inactive to active enough (0.75, 0.55-1.02)<br>Decrease from active enough to inactive (0.77, 0.40-1.47)
Nordstoga, 2019[69]	HUNT Study; Norway; N= 34,257	52% women;	(11-year PA change)	Leisure-time PA measured by	Norwegian Cause of Death	Inactive (no exercise), active (any extent of	Cox regression	Age, sex, education, smoking status, alcohol consumption, insomnia symptoms and history of CVD	**All-cause mortality (N=8449):**<br>Remained active (ref)

		≥20 years	PA from 1984–1986 to 1995–1997; Median follow-up of 17 years from 1995-1997	questionnaires; assessed in weekly frequency and duration	Registry	exercise)			Inactive to active (1.22, 1.08-1.38)<br>Active to inactive (1.35, 1.21-1.50)<br>Remained inactive (1.29, 1.08-1.53)<br><br>**CVD mortality (N=3264):**<br>Remained active (ref)<br>Inactive to active (1.32, 1.10-1.59)<br>Active to inactive (1.60, 1.36-1.86)<br>Remained inactive (1.37, 1.05-1.79)
Østergaard, 2018[70]	Diet, Cancer and Health study; Denmark; N= 28,204	51% women; 50-64 years	(5-year PA change)<br><br>PA change between 1993-1997 and 5 years later; follow-up of 11 years from second examination	Total cycling, measured via questionnaires; assessed in weekly minutes	Civil Registration System	0, 1–60, 61–150, and >150 min/week	Cox regression	Age, gender, years of basic school, higher education, PA at work, leisure-time PA, smoking, monounsaturated, polyunsaturated fat, and saturated fat and coffee intake	**All-cause mortality (N=2942):**<br>No cycling (ref)<br>Stopped cycling (0.98, 0.87-1.11)<br>Initiated cycling (0.78, 0.67-0.90)<br>Consistent cycling (0.77, 0.71-0.84)
Paffenbarger, 1994[71]	Harvard College Alumni Study; USA; N= 14786	0% women; 45–84 years	(11 to 15-year PA change)<br><br>PA measured between 1962-66 and 1977; 12-year follow-up from 1977	Total PA measured via questionnaire; assessed in kcal/week	Death certificates	Active: >=1500 kcal/week	Poisson regression	Age, smoking, hypertension, overweight for height, alcohol, early parental death and chronic diseases	**All-cause mortality (N=2343):**<br>(>=1500 kcal/week)<br>No-no (ref)<br>No-yes (0.72, 0.64-0.82)<br>Yes-no (1.13, 1.01-1.26)<br>Yes-yes (0.77, 0.69-0.85)
Petersen, 2012[72]	Copenhagen City Heart Study; Denmark; N= 10,443	57% women; 20-93 years	(5-year PA change)<br><br>PA change from 1976-1978 to 1981-1983; follow-up of 37 years from 1981–1983	Leisure-time PA measured by questionnaires; assessed in weekly duration (hours) of various intensity	Danish Register of Causes of Death	Sedentary (<2h light PA), light (2-4h light PA) and moderate/vigorous (>4h light PA or >2h vigorous PA)	Cox regression	Age, education, smoking habits, alcohol consumption, BMI, diabetes, cholesterol and BP lowering therapy in 1981–1983	**All-cause mortality (N=6494):**<br>Men:<br>Remained moderate/vigorous (ref)<br>Remained sedentary (1.70, 1.46-2.00)<br>Sedentary to moderate/vigorous (1.24, 1.02-1.50)<br>Moderate/vigorous to sedentary (1.42, 1.01-1.80)<br><br>Women:<br>Remained moderate/vigorous (ref) Remained sedentary (1.58, 1.35-1.87)<br>Sedentary to moderate/vigorous (1.40, 1.13-1.75)<br>Moderate/vigorous to sedentary (1.31, 1.01-1.68)
Saint-Maurice, 2019[73]	NIH-AARP Diet and Health Study; USA; N= 315,059	42% women; 50-71 years	(PA over adulthood)<br><br>PA changes between age 15–18, 19–29, 35–39, and 40–61 years (10 years before 1995–1996); mean follow-up of 13.6 years from 1995–1996	Leisure-time PA measured by questionnaires; assessed in weekly duration in hours	Data linkage	0 hours per week, weekly but <1 hour, 1-3 hours/week, 4-7 hours/week, >7 hours/week	Group-based trajectory modelling (GBTM) and cox regression	Age, sex, education, race, smoking, energy intake, diet percent fat, alcohol, consumption of red meat, fruit and vegetable, vitamin/mineral supplementation and BMI at age 18	**All-cause mortality (N=71377):**<br>Consistently sedentary (ref)<br>Consistently low PA (0.84, 0.81-0.87)<br>Consistently moderate (0.66, 0.63-0.68)<br>Consistently high (0.71, 0.68-0.73)<br>Low-high (0.68, 0.65-0.72)<br>Low-moderate (0.65, 0.62-0.68)<br>High-low (0.96, 0.92-1.00)<br>Moderate-low (0.86, 0.83-0.90)<br><br>**CVD mortality (N=22219):**<br>Consistently sedentary (ref)<br>Consistently low PA (0.82, 0.77-0.88)<br>Consistently moderate (0.58, 0.54-0.63)<br>Consistently high (0.66, 0.62-0.70)<br>Low-high (0.58, 0.53-0.64)<br>Low-moderate (0.57, 0.53-0.61)<br>High-low (0.96, 0.90-1.03)<br>Moderate-low (0.86, 0.81-0.92)<br><br>**Cancer mortality (N=16388):**<br>Consistently sedentary (ref)<br>Consistently low PA (0.96, 0.88-1.04)<br>Consistently moderate (0.85, 0.78-0.93)<br>Consistently high (0.90, 0.83-0.97)<br>Low-high (0.83, 0.75-0.93)<br>Low-moderate (0.84, 0.77-0.92)<br>High-low (0.99, 0.91-1.08)<br>Moderate-low (0.99, 0.92-1.08)
Sanchez-Sanchez, 2020[74]	Toledo Study of Healthy Aging;	58% women; ≥65	(4-year PA trajectory)	Total PA measured by questionnaire;	Spanish National Death	Low, moderate, high	Group-based trajectory	Age, gender, Charlson Index, Mini-Mental State Examination, educational level, smoking status, Katz Index	**All-cause mortality (N=N.A.):**<br>High PA-consistent (ref)

	Spain; N= 1,679	years	PA change from 2006-2009 to 2011-2013; Mean 5.9 years from 2011–2013	assessed in PASE score	Index		modelling (GBTM) and cox regression	and polypharmacy	Moderate PA-decreasing (1.01, 0.74-1.39)<br>Low PA-increasing (1.26, 0.89-1.78)<br>Moderate PA-consistent (0.97, 0.68-1.40)<br>Low PA-decreasing (1.68, 1.21-2.31)
Schnohr, 2017[75]	Copenhagen City Heart Study; Denmark; N=12,314	56% women; 20-93 years	(5- to 27-year PA change)<br><br>PA change between 1976–1978, 1981–1983, 1991–1994, and 2001–2003 (at least 2 examinations); up to 33-year follow-up (all-cause); up to 31-year follow-up (CHD) from second examination	Leisure-time PA measured by questionnaires; assessed in MET.h/week	Danish Central Person Register (all-cause); Register of Causes of Death (CHD)	Sedentary (7 MET.h/week), light (17 MET.h/week), moderate (21 MET.h/week), high (30 MET.h/week)	Cox regression (time-varying covariates)	Age, sex, smoking, education, income, drinking habits, diabetes, examination number and incident CHD and cancer admissions during follow-up	**All-cause mortality (N=7841):**<br>Consistent-sedentary (ref)<br>Consistent-light (0.75, 0.69-0.80)<br>Consistent-moderate (0.62, 0.57-0.68)<br>Consistent-high (0.56, 0.46-0.69)<br><br>**CHD mortality (N=1089):**<br>Consistent-sedentary (ref)<br>Consistent-light (0.76, 0.63-0.92)<br>Consistent-moderate (0.52, 0.41-0.67)<br>Consistent-high (0.51, 0.30-0.88)
Schnohr, 2003[76]	Copenhagen City Heart Study; Denmark; N= 7,023	54.2% women; 20-93 years	(5-year PA change)<br><br>PA change from 1976-1978 to 1981-1983; followed up to 2000 from 1981-1983	Leisure-time PA measured by questionnaires; assessed based on weekly intensity and duration	National Central Person Register	Low (<2h/week light PA), moderate (2-4h/week light PA), and high (>4h/week light PA or >2h/week vigorous PA)	Cox regression	smoking, cholesterol, systolic blood pressure, diabetes, alcohol intake, BMI, education, and income measured at the second examination; analyses stratified by baseline PA and gender	**All-cause mortality (N=2725):**<br>Men:<br>Low-low (ref)<br>Low-moderate (0.64, 0.49-0.83)<br>Low-high (0.64, 0.47-0.87)<br>Moderate-low (0.73, 0.56-0.96)<br>Moderate-moderate (0.71, 0.57-0.88)<br>Moderate-high (0.64, 0.51-0.81)<br>High-low (1.11, 0.76-1.62)<br>High-moderate (0.66, 0.51-0.85)<br>High-high (0.61, 0.48-0.76)<br><br>Women:<br>Low-low (ref)<br>Low-moderate (0.75, 0.57-0.97)<br>Low-high (0.72, 0.50-1.05)<br>Moderate-low (0.70, 0.54-0.91)<br>Moderate-moderate (0.64, 0.52-0.79)<br>Moderate-high (0.58, 0.45-0.73)<br>High-low (0.72, 0.48-1.07)<br>High-moderate (0.61, 0.47-0.80)<br>High-high (0.66, 0.51-0.85)
Shaw, 2014[77]	The level-of-living survey (LNU) and the Swedish panel study of living conditions of the oldest old (SWEOLD); Sweden; N= 1,682	51% women; 33-55 years	(13-year PA change)<br><br>PA change from 1968 to 1981; followed up to 26 years from 1981	Total PA measured by questionnaire; assessed by a summary score	Swedish National Cause of Deaths Register	Inactive (summary score <2 points), active (summary score ≥2 points)	Cox regression	Age, sex, education, social class, and health problems (circulatory, mobility and psychological) in 1981	**All-cause mortality (N=875):**<br>Remained active (ref)<br>Inactive to active (1.0, 0.8-1.3)<br>Active to inactive (1.2, 1.0-1.4)<br>Remained inactive (1.2, 1.0-1.5)
Shortreed, 2013[78]	Framingham Heart Study; USA; N= 4,729	57% women; 38-68 years	(PA over adulthood)<br><br>PA change between 1956, 1970/1972, and 1986/1988; 40-year follow-up from 1956 to 1996	Total PA measured by self-report; assessed in summary score based on average daily hours in sedentary, slight, moderate and heavy PA	FHS, medical and hospitalization records	Inactive (<30.2), active (≥30.2)	Marginal structural model and logistic regression (marginal rate ratio calculated)	Baseline covariates (age, sex, education, job type, birth country, weight at 25 years, height) and covariates at follow-up visits: BMI, smoking, marital status, alcohol, blood glucose and diagnosis of a chronic disease (diabetes or cancer) or comorbid condition (arthritis, left ventricular hypertrophy, ankle oedema, pulmonary disease)	**All-cause mortality (N=3521):**<br>Always inactive (ref)<br>Always active (0.81, 0.71-0.93)<br>Always active in men (0.78, 0.64-0.95)<br>Always active in women (0.90, 0.74-1.16)<br><br>**CVD mortality (N=1313):**<br>Always inactive (ref)<br>Always active (0.83, 0.72-0.97)<br>Always active in men (0.67, 0.52-0.88)<br>Always active in women (1.00, 0.75-1.33).
Stamatakis, 2024[79]	Taiwan MJ Cohort; China; N= 105,461	49.7% women; ≥18 years	(6.3-year PA change)<br><br>PA measured between 1998-2016, two measures 6.3 years apart; 12.5 years follow-up for mortality	Occupational PA measured by questionnaires; assessed in intensities	Death data linkage	Light (mostly sedentary), moderate (repetitive motions while sitting or standing), moderately heavy (mostly standing or walking), heavy (heavy lifting, loading, or moving loads)	Cox regression	Sleep duration, alcohol, smoking, leisure-time physical activity, BMI, stratified by sex; participants with self-reported CVD or cancer were excluded	**All-cause mortality (N=20676):**<br>Men:<br>Maintained (ref)<br>Decreased (1.16, 1.01-1.33)<br>Increased (1.13, 0.98-1.30)<br><br>Women:<br>Maintained (ref)<br>Decreased (1.08, 0.93-1.26)<br>Increased (0.83, 0.70-0.97)

									**CVD mortality (N=3508):**<br>Men:<br>Maintained (ref)<br>Decreased (1.07, 0.76-1.51)<br>Increased (0.85, 0.59-1.24)<br><br>Women:<br>Maintained (ref)<br>Decreased (0.92, 0.63-1.34)<br>Increased (0.98, 0.67-1.44)<br><br>**Cancer mortality (N=8320):**<br>Men:<br>Maintained (ref)<br>Decreased (1.20, 0.99-1.46)<br>Increased (1.07, 0.85-1.33)<br><br>Women:<br>Maintained (ref)<br>Decreased (1.03, 0.83-1.28)<br>Increased (0.83, 0.65-1.05)
Trolle-Lagerros, 2005[80]	Women's Lifestyle and Health; Norway & Sweden; N= 99,099	100% women; 30-49 years	(10-year PA change from age 30)<br><br>PA measured at age 14, age 30 and at enrolment; 11.4 years follow-up for mortality	Total PA measured by questionnaires; assessed in scores by intensity and frequency	Death data linkage	None (1); low (2); moderate (3); high (4); vigorous (5)<br><br>Inactive (1,2); active (3,4,5)	Cox regression	Age at enrolment, years of education, BMI, alcohol intake, smoking status, number of cigarettes, years of smoking and country of origin	(PA change from age 30 to enrolment)<br><br>**All-cause mortality (N=1313):**<br>Remained inactive (ref)<br>Active to inactive (1.18, 0.95-1.47)<br>Inactive to active (0.75, 0.54-1.04)<br>Remained active (0.76, 0.63-0.92)
Vaes, 2014[81]	Copenhagen City Heart Study; Denmark; N= 8,734	57.6% women; ≥20 years	PA measured in 4 examinations, subjects with at least 2 consecutive examinations were included; mean follow-up of 17.1 years	Total PA measured by questionnaires; assessed in weekly intensity and duration	No information	Low (light PA<2h/week); moderate (light PA 2-4h/week); high (light PA > 4h/week)	Cox regression	Sex, age, self-reported comorbidities, smoking, smoking years, education, forced expiratory volume in 1 s and BMI	**All-cause mortality (N=5392):**<br>Remained low PA/sedentary (ref)<br>Low-moderate (0.707, 0.592-0.844)<br>Low-high (0.747, 0.599-0.931)
Wannamethee, 1998[82]	British Regional Heart Study; UK; N= 4260	0% women; 40–59 years	(12 to 14-year PA change)<br><br>PA measured between 1978-80 and 1992; 4-year follow-up for all-cause mortality; 3-year follow-up for CVD from 1992	Leisure-time PA measured via questionnaire; assessed in PA scores	National Health Service registers in Southport (England, Wales) and Edinburgh (Scotland)	Inactive (score 0–2); occasional (score 3–5: regular walking or recreational activity only); light (score 6–8: more frequent recreational activities, or sporting exercise less than once a week); moderate (score 9–12: cycling, or sporting activity once a week); moderately vigorous (score 13–20: sporting activity at least once a week); vigorous (score 21 or more: very frequent sporting exercise)	Cox regression	Age, smoking, social class, BMI and self-perception of health	**All-cause mortality (N=217):**<br>Remained inactive (ref)<br>Increased to at least light PA (0.55, 0.36-0.84)<br>Decreased to inactive (0.75, 0.50-1.14)<br>Remained at least light PA (0.58, 0.41-0.82)<br><br>**CVD mortality (N=92):**<br>Remained inactive (ref)<br>Increased to at least light PA (0.66, 0.35-1.23)<br>Decreased to inactive (1.02, 0.56-1.86)<br>Remained at least light PA (0.54, 0.31-0.94)
Williamson, 2019[83]	Melbourne Collaborative Cohort Study; Australia; N= 22,213	62.9% women; 40-69 years	8-year hypothetical intervention from 1995-1999 (T1) to 2003-2007 (T2); Median follow-up of 13.6 years from 1995–1999	Population-based hypothetical PA intervention	Victorian Registry of Births, Deaths and Marriages, and the National Death Index	Low (mostly walking), moderate, high (intensive PA ≥3 times/week)	Parametric g-formula	T0: sex, ethnicity, education, alcohol, BP, cholesterol, family history of heart attack; T1: age, BMI, smoking, living alone, history of angina, arthritis and asthma; T2: living alone, BMI, smoking, cholesterol	**All-cause mortality (N=2163):**<br>Low PA at both T1 and T2 (ref)<br>Single intervention of high PA at T1 (0.77, 0.68-0.87)<br>Intervention of high PA at both T1 and T2 (0.73, 0.63-0.83)
Wolin, 2010[84]	Cancer Prevention Study (CPS) II Nutrition Cohort; USA; N= 158,253	53% women; 40-92 years	(10-year PA change)<br><br>PA change from 1982 to 1992; follow-up of 14 years from 1992	Leisure-time PA measured by questionnaire; assessed in intensity and MET.h/week	National Death Index	Low (<17.5 MET.h/week), high (≥17.5 MET.h/week)	Cox regression	Sex, education, smoking, use of postmenopausal hormones (among women only), intake of multivitamin, red meat, folate, alcohol, NSAIDs, fibre, calcium, total energy and BMI	**Colon cancer mortality (N=846):**<br>Consistently low (ref)<br>Consistently high (0.86, 0.69-1.06)<br>Increasing (1.08, 0.77-1.51)<br>Decreasing (0.91, 0.77-1.09)

Xue, 2012[85]	Women's Health and Aging Study (WHAS) II; USA; N=433	100% women; 70–79 years	(median 12-year PA trajectory)<br><br>PA assessed in 1994 and 6 follow-up visits (18 months apart); followed up for mortality till 2009	Leisure-time PA assessed via questionnaire; assessed in kcal/day/kg	Follow-up interviews with proxies, obituaries, and matching with the National Death Index	Inactive (<1.5 kcal/day/kg), moderately active (≥1.5 and <3 kcal/day/kg), and very active (≥3 kcal/day/kg)	Cox regression; latent class analysis model	Age, race, educational level and NHIS activity status at baseline	**All-cause mortality (N=149):**<br>Always active (ref)<br>Fast declining (2.34, 1.20-4.59)<br>Stable moderate (1.24, 0.63-2.47)<br>Always sedentary (3.34, 1.72-6.47)
Yang, 2021[86]	Australian Diabetes, Obesity and Lifestyle Study (AusDiab); Australia; N= 6,377	54.7% women; mean age 56.5 years in 2004–2005	(7-year PA change)<br><br>PA change between 2004-2005 to 2011-2012; mean follow-up 13 years from 2004-2005	Population-based hypothetical PA intervention; assessed in total weekly leisure-time physical activity (min/week) based on self-reported frequency and duration of LTPA	Australian National Death Index	Insufficiently active (<150 min/week), sufficient active (150–300 min/week), optimal active (>300 min/week)	Parametric g-formula	Time-fixed: sex, baseline age, quintiles of an area-based index of relative socio-economic advantage and disadvantage, country of birth and level of education.<br><br>Time-varying: self-reported history of high cholesterol, high blood pressure, heart disease, and diabetes and self-reported general health status; waist circumference, Mediterranean diet score, smoking status and alcohol intake	**All-cause mortality (N=781):**<br>Remained inactive (worst scenario) (ref)<br>Sustained PA at >300min/week (0.66, 0.46-0.86)
Yin, 2023[87]	Chinese Longitudinal Healthy Longevity Survey (CLHLS); China; N= 22,463	57% women; mean 91.1 years	Current and past PA measured in 7 surveys from 1998 to 2014; median follow-up of 3 years for mortality to 2018	Exercise PA measured by questionnaires; assessed by exercise status	Death certificates or interview	Inactive ("no" response), active ("yes" response)	Accelerated failure time Weibull survival regression models (event time ratio calculated)	Age, sex, socioeconomic factors (residence, marital status, education, occupation, and ethnicity), lifestyle factors (smoking and alcohol consumption), chronic diseases	**All-cause mortality (N=15707):**<br>Remained inactive (ref)<br>Remained active (1.14, 1.11-1.17)<br>Inactive to active (1.14, 1.08-1.20)<br>Active to inactive (0.99, 0.94-1.04)
**Time-varying PA**									
Byberg, 2009[40]	Uppsala Longitudinal Study of Adult Men; Sweden; N= 2205	0% women; 49-51 years	(up to 35-year PA)<br><br>PA measured in ages 50, 60, 70, 77 and 82; 35-year follow-up from age 50	Leisure-time PA measured by questionnaire; assessed in intensity and duration	Swedish National Population Register	low (mostly sedentary); medium (often walking/cycling for pleasure); high (active sport at least 3 hours per week or hard physical training)	Time-varying cox regression	Smoking, obesity, height and weight, self-perceived health, PA at work, diabetes, any musculoskeletal, neurological or psychiatric disorders, alcohol use, BP, use of antihypertensive drugs, total serum cholesterol, education and socio-economic group	**All-cause mortality (N=1329):**<br>High (ref)<br>Medium (1.22, 1.08-1.37)<br>Low (1.40, 1.18-1.66))
Hamer, 2014[88]	English Longitudinal Study of Ageing (ELSA); England; N= 10,426	54.4% women; ≥50 years	(6-year PA)<br><br>PA measured from 2002 to 2008, at every 2 years; average follow-up of 7.8 years	Total PA measured by questionnaires, based on the frequency of participation in vigorous, moderate, and mild physical activities	National Health Service registries	Inactive, vigorous, moderate, and mild PA	Time-varying Cox regression	Age, gender, marital status, socioeconomic position, time-varying covariates (self-reported chronic diseases, smoking, depressive symptoms, BMI and waist circumference)	**All-cause mortality (N=1896):**<br>Physical inactivity (ref)<br>Mild PA 0.76 (0.69-0.83)<br>Moderate PA 0.54 (0.49-0.59)<br>Vigorous PA 0.44 (0.39-0.50)<br><br>**CVD mortality (N=671):**<br>Physical inactivity (ref)<br>Mild PA 0.74 (0.64-0.85)<br>Moderate PA 0.48 (0.41- 0.55)<br>Vigorous PA 0.46 (0.38-0.57)<br><br>**Cancer mortality (N=596):**<br>Physical inactivity (ref)<br>Mild PA 1.02 (0.85-1.24)<br>Moderate PA 0.86 (0.72-1.03)<br>Vigorous PA 0.65 (0.52-0.81)
Joseph, 2019[89]	Copenhagen City Heart Study; Denmark; N= 18,974	54% women; ≥20 years	(27-year PA)<br><br>PA measured in 1976-1978, 1981-1983, 1991-1994, 2001-2003; mean follow-up of 23.4 years from baseline	Leisure-time PA measured by questionnaires; assessed in weekly duration & intensities	Danish Register of Causes of Death	Inactivity, light activity (light PA 2-4 h/week), moderate/high activity (light PA >4h/week or vigorous PA >2h/week)	Time-varying Cox regression	Age, sex, smoking status, education, diabetes, previous CVD, BMI and calendar time. Stratified by blood pressure	**All-cause mortality (N=13355):**<br>Inactivity (ref)<br>Light activity (0.76, 0.64-0.91)<br>Moderate activity (0.68, 0.56-0.84)
Kaplan, 1996[90]	The Alameda County Study; USA; N= 6131	54% women; 16–94 years	(18-year PA)<br><br>PA measured in 1965, 1974, and 1983; 28-year follow-up from 1965	Leisure-time PA assessed via questionnaire; frequency and intensity of different activities assessed	California State mortality tapes; death certificates	Never, sometimes, or often and were scored as 0, 2, or 4; PA scale had a range of 0 to 12 for each follow-up	Time-varying cox regression	Time-fixed: age, sex, ethnicity<br>Time-varying: education, health conditions, smoking, BMI, social isolation	**All-cause mortality (N=1226):**<br>Per 4-point increase in the 12-point PA scale (0.84, 0.77-0.92)<br><br>**CVD mortality (N=709):**<br>Per 4-point increase in the 12-point PA scale (0.81, 0.71-0.93)
Lantz, 2010[91]	Americans'	53%	(15-year PA)	Total PA measured	National Death	Index score divided into	Time-varying Cox	Age, gender, race, residence, education, income, smoking,	**All-cause mortality (N=1409):**

	Changing Lives (ACL) longitudinal study; USA; N= 3,617	women; ≥25 years	PA measured in 1986, 1989, 1994, 2001/2002; followed up to 19 years from baseline	by questionnaires; assessed in PA index	Index & death certificates	5 sub-groups of comparable size	regression	alcohol use, BMI, physical impairment, and self-rated health	Quintile 1 (sedentary) (ref)<br>Quintile 2 (0.74, 0.49-1.13)<br>Quintile 3 (0.68, 0.47-1.00)<br>Quintile 4 (0.73, 0.49-1.10)<br>Quintile 5 (most active) (0.63, 0.48-0.83)
Lee, 2003[92]	The College Alumni Health Study; USA; N= 32,687	7% women; mean age at baseline 47. 1 years	(33-year PA)<br><br>PA measured in Harvard: 1962/1966, 1977, 1988, 1993; PA measured in Pennsylvania: 1962, 1980, 1993; followed up to 1995	Total PA measured by questionnaires, assessed in energy expenditure per week	Death certificates	<2100, 2100-4199, 4200-10499, and ≥10500 kJ/week	Time-varying Cox regression	Age, sex, smoking and diabetes	**Pancreatic cancer mortality (N=212):**<br><2100 kJ/week (ref)<br>2100-4199 kJ/week (0.98, 0.65-1.49)<br>4200-10499 kJ/week (0.92, 0.62-1.35)<br>>10500 kJ/week (1.31, 0.69-1.92)
Lee, 2004[93]	Harvard Alumni Health Study; USA; N= 8,421	0% women; mean 66 years	(5-year PA)<br><br>PA measured in 1988 and 1993; followed up to 1997	Total PA measured by questionnaires; assessed in energy expenditure per week	Death certificates	"sedentary" (expending <500 kcal/week), "insufficiently active" (500-999 kcal/week), "weekend warriors" (≥1,000 kcal/week from sports/recreation 1-2 times/week), or "regularly active" (all others expending ≥1,000 kcal/week)	Time-varying Cox regression	Age, cigarette smoking, alcohol consumption, red meat intake, vegetable intake, vitamin/mineral supplements, and early parental mortality; chronic diseases excluded at baseline	**All-cause mortality (N=1234)**:<br>Sedentary (ref)<br>Insufficiently active (0.75, 0.62-0.91)<br>Weekend warriors (0.85, 0.65-1.11)<br>Regularly active (0.64, 0.55-0.73)
O'Donovan, 2024[94]	Mexico City Prospective Study; Mexico; N=10,023	67% women; ≥35 years	(15-year PA)<br><br>PA measured from 1998-2004 to 2015-2019; mean follow-up of 19.7 years mortality follow-up to 2020	Leisure-time PA measured by questionnaires; assessed in frequency and session duration	Data linkage	No sport or exercise, weekend warrior (once/twice per week), regularly active (three or more per week)	Time-varying Cox regression	Baseline measures of age and sex, and baseline and resurvey measures of education, income, smoking, fruit and vegetable intake, and alcohol	**All-cause mortality (N=843):**<br>Being inactive or becoming inactive (ref)<br>Being a weekend warrior or becoming a weekend warrior (0.86, 0.65-1.12)<br>Regularly active or becoming regularly active (0.85, 0.70-1.03)
Opdal, 2021[95]	The Tromsø Study (TS); Norway; N= 12,241	52% women; 25-87 years	(13-year PA)<br><br>PA measured in 1994-95, 2001 and 2007-08; mean follow-up of 18.8 years	Light and hard leisure-time PA measured by questionnaires, based on weekly duration	Norwegian National Causes of Death Registry	Sedentary, some PA (<1h/week); moderate PA (1-2h/week), vigorous PA (≥3h/week)	Time-varying Cox regression	Time fixed: Age, sex<br>Time-dependent: co-morbidities, mental health symptoms, CVD risk factors, BMI, smoking habits, self-reported health	**All-cause mortality (N=N.A.):**<br>No light PA (ref)<br>Light PA <1 h/week (1.42, 0.80-2.53)<br>Light PA 1-2 h/week (1.22, 0.75-1.98)<br>Light PA >3 h/week (1.03, 0.71-1.50)<br><br>Sedentary (ref)<br>Low hard PA (0.74, 0.58-0.95)<br>Moderate hard PA (0.68, 0.53-0.86)<br>Vigorous hard PA (0.64, 0.55-0.75)
Patterson, 2020[96]	The office for National Statistics Longitudinal Study of England and Wales; UK; N=394,746	46.9% women; ≥16 years	Exposure measured in at least one eligible census (1991, 2001, and 2011); followed up for mortality until 2016	Commute mode measured by questionnaires	Death registration	Private motorised mode, public transport, walking, or cycling	Time-varying Cox regression	Age, sex, housing tenure, marital status, ethnicity, university education, car access, population density, socioeconomic classification, Carstairs index quintile, long-term illness, and year entered the study, stratified by socioeconomic group	**All-cause mortality (N=13983):**<br>Inactive commuting (ref)<br>Cycling (0.80, 0.73–0.89)<br>Walking (0.98, 0.92-1.03)<br><br>**CVD mortality (N=3172):**<br>Inactive commuting (ref)<br>Cycling (0.76, 0.61–0.93)<br>Walking (0.91, 0.81-1.03)<br><br>**Cancer mortality (N=6509):**<br>Inactive commuting (ref)<br>Cycling (0.84, 0.73–0.98)<br>Walking (0.98, 0.91-1.06)
Reinikainen, 2015[97]	Finnish cohorts of the Seven Countries Study; Finland; N= 1,540	0% women; 40-59 years	(10-year PA)<br><br>PA change between 1959, 1964 and 1969; median follow-up of 23.1 years from baseline	Occupational PA measured by questionnaires	National Causes of Death Register	Sedentary, light work, heavy work	Time-varying Cox regression	SBP, cholesterol, heart rate, smoking	**CVD mortality (N=850):**<br>Heavy work (ref)<br>Sedentary 1.81 (1.18, 2.79)<br>Light work 1.31 (1.13, 1.53)

Sabia, 2012[98]	Whitehall II Study; UK; N= 7,456	30.1% women; mean 55.9 years	(5-year PA)<br><br>PA measured in 1997-99 and 5 years later; mean follow-up of 9.6 years	Total PA and PA types and intensity measured by questionnaires; assessed in hour/week	National Health Services Central Registry	<1h/week; 1.0-3.4h/week; ≥3.5h/week	Time-varying Cox regression	Age, mutually physical activity intensity levels/types, gender, socioeconomic status, and marital status, smoking, alcohol consumption, fruit and vegetable consumption, diabetes, coronary heart disease, stroke, and self-rated health	**All-cause mortality (N=317):**<br>Mild activity:<br><5.5h/week (ref)<br>5.5-8.9h/week (0.85, 0.64-1.12)<br>≥9h/week (0.93, 0.71-1.23)<br><br>Moderate activity:<br><1h/week (ref)<br>1-3.4 h/week (0.67, 0.51-0.88)<br>≥3.5h/week (0.67, 0.50-0.91)<br><br>Vigorous activity:<br>None (ref)<br>0.1-0.9h/week (0.82, 0.62-1.08)<br>≥1h/week (0.90, 0.64-1.28)
Sheehy, 2020[99]	Black Women's Health Study; USA; N= 52,993	100% women; 21-69 years	(20-year PA)<br><br>PA measured from 1995 to 2015, every 2-year interval. Follow-up of 22 years from baseline	Walking exercise and vigorous exercise measured by questionnaires; assessed in hours/week and MET.h/week	National Death Index & death certificate	None, <1, 1-2, 3-4, ≥5 hours/week OR None, 0-7.5, 7.5-15, 15-30, ≥30 MET.h/week	Time-varying Cox regression	Age, calendar year, neighbourhood SES, years of education, BMI, cigarette smoking, alcohol consumption, diet quality, hypertension, diabetes	**(vigorous PA in hour/week)**<br><br>**All-cause mortality (N=4719):**<br>None (ref)<br><1 h/week (0.74, 0.68-0.81)<br>1-2 h/week (0.67, 0.60-0.74)<br>3-4 h/week (0.53, 0.46-0.61)<br>>=5 h/week (0.58, 0.50-0.67)<br><br>**CVD mortality (N=1697):**<br>None (ref)<br><1 h/week (0.74, 0.62-0.88)<br>1-2 h/week (0.54, 0.43-0.68)<br>3-4 h/week (0.57, 0.43-0.74)<br>>=5 h/week (0.66, 0.50-0.87)<br><br>**Cancer mortality (N=1256):**<br>None (ref)<br><1 h/week (0.80, 0.66-0.96)<br>1-2 h/week (0.67, 0.54-0.84)<br>3-4 h/week (0.48, 0.35-0.64)<br>>=5 h/week (0.52, 0.38-0.72)
Stessman, 2009[100]	Jerusalem Longitudinal Cohort Study; Israel; N= 1,861	43% women; 70-88 years	(15-year PA)<br><br>PA measured at age 70, 78, and 85; follow-up to 2008	Total PA measured by questionnaires, based on weekly duration	Death certificate	Inactive (<4h/week), active (≥4h/week)	Time-varying Cox regression	Sex, financial status, origin, BMI, smoking, ease of performance in ADLs, hypertension, ischaemic heart disease, diabetes, history of neoplasm and renal disease	**All-cause mortality (N=512):**<br>Inactive (ref)<br>Active 0.66 (0.46, 0.95)
Talbot, 2007[101]	Baltimore Longitudinal Study of Aging; USA; N= 2,092	37% women; 19-90+ years	(38-and 18-year rate of LTPA change)<br><br>PA change between baseline and at 2-year intervals from 1958 to 1996 (men)/1978 to 1996 (women); mean 21.2-year follow-up for men, mean 10.2-year follow-up for women	Leisure-time PA measured by questionnaires; calculated in MET.min/day	National death index	NA (continuous exposure)	Cox regression for baseline LTPA and rate of LTPA change during follow-up; Linear mixed-effect model to describe rate of change in PA	Stratified by age and sex, adjusted for BMI, current smoking status, total cholesterol, and hypertension	**All-cause mortality (N=628):**<br>A SD increase in baseline LTPA is associated with lower all-cause mortality (0.66, 0.57-0.75) in men<70 years; (0.89, 0.77-1.04) in men >=70 years; (0.68, 0.47-0.97) in women <70 years; (0.77, 0.59-1.03) in women >=70 years<br><br>A SD increase in the rate of change in LTPA is associated with lower all-cause mortality (0.86, 0.77-0.97) in men<70 years; (1.08, 0.94-1.24) in men >=70 years; (1.04, 0.79-1.37) in women <70 years; (1.03, 0.81-1.31) in women >=70 years
Williamson, 2019[83]	Melbourne Collaborative Cohort Study; Australia; N= 22,213	62.9% women; 40-69 years	(8-year PA)<br><br>PA measured from 1995-1999 (T1) to 2003-2007 (T2); Median follow-up 13.6 years from 1995-1999	Total PA measured by questionnaires, based on amount and intensity	Victorian Registry of Births, Deaths and Marriages, and the National Death Index	Low, moderate and high	Time-varying Cox regression	T0: sex, ethnicity, education, alcohol, BP, cholesterol, family history of heart attack; T1: age, BMI, smoking, living alone, history of angina, arthritis and asthma; T2: living alone, smoking, BMI, and plasma cholesterol	**All-cause mortality (N=2163):**<br>low PA (ref)<br>Moderate PA (0.75, 0.67-0.84)<br>High PA (0.71, 0.62-0.81)
**Averaged/cumulative PA**									

Bembom, 2009[102]	The Study of Physical Performance and Age-related Changes in Sonomans; USA; N= 5,233	53% women; ≥54 years	PA measured in 1993-94, 1995-96, 1998-99, and 2000-01; 2-year follow-up from a given survey	Leisure-time PA measured by questionnaires; assessed by frequency in different leisure-time activities	Death certificates	Divided by whether meet the recommendation of 22.5 METs/week: low (<22.5 METs); high (≥22.5 METs)	History-adjusted marginal structural model	Time-fixed: past PA, sex, baseline reported exposure to environmental tobacco smoke.<br><br>Time-varying: BMI, having at least 1 chronic disease, depression score, current use of antidepressant, smoking, overall health, living arrangements and functional limitation	**All-cause mortality (N=235):**<br>Age <75:<br>Low (-0.015, -0.031 — 0.003)<br>High (-0.007, -0.026 — 0.011)<br><br>Age ≥75:<br>Low (-0.078, -0.130 — -0 .033)<br>High (-0.102, -0.179 — -0 .042)
Emberson, 2005[103]	British Regional Heart Study; UK; N= 6,452	0% women; 40-59 years	(20-year PA)<br><br>PA measured in 1978-1981, 1992, 1996, 1998-2000; follow-up of 20 years from baseline	Average leisure-time PA assessed in questionnaires; PA score derived based on frequency	National Health Service registers	None, occasional, light, moderate, moderately vigorous, and vigorous	Cox regression	Age	**All-cause mortality (N=1525):**<br>None (ref)<br>Occasional (0.62, 0.52-0.74)<br>Light (0.45, 0.37-0.54)<br>Moderate (0.38, 0.31-0.47)<br>Moderately vigorous (0.31, 0.25-0.40)<br>Vigorous (0.41, 0.31-0.55)
Fontana, 2024[104]	Turin Longitudinal Study (TLS) 2011; Italy; N= 156,670	51.7% women; mean 39.8 years	(6-year PA)<br><br>PA from 2012 to 2018; 6-year follow-up	Cumulative occupational PA assessed in questionnaires; computed in ergonomic exposure index (Ergo-index)	Death data linkage	Divided into 4 quartiles	Poisson regression (incident rate ratio calculated)	Age, household typology, Charlson index, area deprivation index, educational level	**All-cause mortality (N=N.A.):**<br>Men:<br>First quartile (ref)<br>Second quartile (0.97, 0.79-1.20)<br>Third quartile (1.12, 0.93–1.35)<br>Fourth quartile (1.27, 1.06–1.51)<br><br>Women:<br>First quartile (ref)<br>Second quartile (1.29, 1.02–1.64)<br>Third quartile (1.52, 1.20–1.92)<br>Fourth quartile (1.73, 1.35–2.22)
Holtermann, 2012[105]	Copenhagen City Heart Study; Denmark; N= 7,819	58% women; 25-66 years	(5-year PA)<br><br>PA from 1976-78 to 1981-1983; mortality follow-up to 2010	Cumulative occupational PA assessed by questionnaires based on summary scores 2-8	Civil registration system	Low (2-3), moderate (4-5), high (6-8)	Cox regression	Age, smoking, alcohol, BMI, leisure time physical activity, systolic blood pressure, diabetes, cholesterol, blood pressure medication and household income	**All-cause mortality (N=2888):**<br>Men:<br>Low OPA (ref)<br>Moderate (1.15, 1.01-1.33)<br>High (1.22, 1.05-1.41)<br><br>Women:<br>Low OPA (ref)<br>Moderate (0.88, 0.78-1.00)<br>High (0.95, 0.79-1.13)
Hu, 2004[106]	Nurses' Health Study; USA; N= 116,564	100% women; 30-55 years	(18-year PA)<br><br>PA measured from 1980 to 1998; up to 20 years of follow-up	Cumulative average number of hours of MVPA calculated at each time point	National death index, Death certificates	<1.0 h/week; 1.0-3.4 h/week; ≥3.5 h/week	Cox regression	Age, smoking status, parental history with coronary heart disease; menopausal status and hormone use, alcohol consumption; Stratified by BMI	**All-cause mortality (N=10282):**<br>BMI<25:<br>≥3.5 (ref)<br>1-3.4 (1.18, 1.09-1.29)<br><1.0 (1.55, 1.42-1.70)<br><br>BMI 25-29.9:<br>≥3.5 (1.28, 1.12-1.46)<br>1-3.4 (1.33, 1.20-1.47)<br><1.0 (1.64, 1.46-1.83)<br><br>BMI ≥30:<br>≥3.5 (1.91, 1.60-2.30)<br>1-3.4 (2.05, 1.82-2.30)<br><1.0 (2.42, 2.14-2.73)<br><br>**CVD mortality (N=2370):**<br>BMI<25:<br>≥3.5 (ref)<br>1-3.4 (1.51, 1.22-1.87)<br><1.0 (1.89, 1.51-2.37)<br><br>BMI 25-29.9:<br>≥3.5 (1.58, 1.15-2.16)<br>1-3.4 (2.06, 1.62-2.60)<br><1.0 (2.52, 1.96-3.25)<br><br>BMI ≥30:<br>≥3.5 (2.87, 1.94-4.25)<br>1-3.4 (4.26, 3.33-5.44)

									<1.0 (4.73, 3.68-6.09)<br><br>**Cancer mortality (N=5223):**<br>BMI<25:<br>≥3.5 (ref)<br>1-3.4 (1.09, 0.98-1.22)<br><1.0 (1.32, 1.17-1.50)<br><br>BMI 25-29.9:<br>≥3.5 (1.22, 1.02-1.45)<br>1-3.4 (1.20, 1.05-1.38)<br><1.0 (1.39, 1.19-1.62)<br><br>BMI ≥30:<br>≥3.5 (1.57, 1.21-2.03)<br>1-3.4 (1.44, 1.21-1.71)<br><1.0 (1.68, 1.40-2.01)
Huerta, 2016[107]	European Prospective Investigation into Cancer and Nutrition study (EPIC)-Spanish branch; Spain; N= 38,379	62% women; 30-65 years	(3.3-year PA)<br><br>PA measured in 1992-1996 and 3.3 years later; mean follow-up of 13.6 years	Occupational activity; average household activity (MET.h/week); average leisure-time PA (MET.h/week); measured by questionnaires	Spanish National Statistics Institute	Leisure-time PA categorised into <7.5, 7.5-14.9, 15.0-29.9, and ≥30.0 MET.h/week	Cox regression	Centre, educational level, BMI, waist and hip circumference, baseline hypertension, hyperlipidaemia, or diabetes, smoking, alcohol consumption, energy intake, Mediterranean diet score, mutual PA variables	(average leisure-time PA)<br><br>**All-cause mortality (N=1371):**<br>Men:<br><7.5 MET.h/week (ref)<br>7.5-14.9 MET.h/week (1.06, 0.79-1.42)<br>15.0–29.9 MET.h/week (0.96, 0.73–1.26)<br>≥ 30.0 MET.h/week (0.96, 0.73–1.25)<br><br>Women:<br><7.5 MET.h/week (ref)<br>7.5-14.9 MET.h/week (0.75, 0.54-1.05)<br>15.0–29.9 MET.h/week (0.87, 0.65–1.16)<br>≥ 30.0 MET.h/week (0.71, 0.52–0.98)<br><br>**CVD mortality (N=291):**<br>Men:<br><7.5 MET.h/week (ref)<br>7.5-14.9 MET.h/week (1.15, 0.64-2.06)<br>15.0–29.9 MET.h/week (1.11, 0.65–1.91)<br>≥ 30.0 MET.h/week (0.82, 0.47–1.43)<br><br>Women:<br><7.5 MET.h/week (ref)<br>7.5-14.9 MET.h/week (1.09, 0.46-2.58)<br>15.0–29.9 MET.h/week (1.18, 0.54–2.60)<br>≥ 30.0 MET.h/week (0.64, 0.26–1.58)<br><br>**Cancer mortality (N=758):**<br>Men:<br><7.5 MET.h/week (ref)<br>7.5-14.9 MET.h/week (0.96, 0.63-1.46)<br>15.0–29.9 MET.h/week (0.96, 0.66–1.40)<br>≥ 30.0 MET.h/week (0.86, 0.59–1.24)<br><br>Women:<br><7.5 MET.h/week (ref)<br>7.5-14.9 MET.h/week (0.81, 0.50-1.29)<br>15.0–29.9 MET.h/week (1.09, 0.72–1.65)<br>≥ 30.0 MET.h/week (0.96, 0.62–1.47)
Lee, 2022[108]	Nurses' Health Study (NHS) & Health Professionals Follow-up Study (HPFS); USA; N= 116,221	63% women; 40-76 years	(up to 30-year PA)<br><br>PA measured from 1988 and updated every 2 years to up to 15 repeated measures; median follow-up of 26 years	Leisure-time PA assessed by questionnaire; calculated in cumulative average	National death index	MPA and VPA divided into 0-19/0, 20-74/1-74, 75-149, 150-224, 225-299, 300-374, 375-449, 450-599, ≥600 min/week	Cox regression	Year, cohort, race, family history of CVD, family history of cancer, postmenopausal hormone use, alcohol intake, total energy intake, smoking status, sleep duration, Alternate Healthy Eating Index score, and BMI	**All-cause mortality (N=47596):**<br>VPA:<br>0 (ref)<br>1–74 (0.87, 0.82–0.93)<br>75–149 (0.81, 0.76–0.87)<br>150–224 (0.79, 0.74–0.85)<br>225–299 (0.77, 0.72–0.84)<br>300–374 (0.78, 0.72–0.85)<br>375–449 (0.73, 0.65–0.81)<br>450–599 (0.76, 0.68–0.85)<br>≥600 (0.74, 0.65–0.85)

									MPA:<br>0-19 (ref)<br>20-74 (0.91, 0.88–0.94)<br>75–149 (0.84, 0.81–0.88)<br>150–224 (0.80, 0.77–0.83)<br>225–299 (0.79, 0.76–0.82)<br>300–374 (0.74, 0.70–0.77)<br>375–449 (0.74, 0.70–0.78)<br>450–599 (0.69, 0.65–0.73)<br>≥600 (0.68, 0.64–0.73)<br><br>**CVD mortality (N=10410):**<br>VPA:<br>0 (ref)<br>1–74 (0.76, 0.67–0.86)<br>75–149 (0.69, 0.60–0.78)<br>150–224 (0.73, 0.64–0.85)<br>225–299 (0.67, 0.57–0.79)<br>300–374 (0.68, 0.56–0.82)<br>375–449 (0.65, 0.52–0.82)<br>450–599 (0.67, 0.53–0.84)<br>≥600 (0.71, 0.54–0.93)<br><br>MPA:<br>0-19 (ref)<br>20-74 (0.87, 0.81–0.94)<br>75–149 (0.79, 0.73–0.85)<br>150–224 (0.78, 0.72–0.84)<br>225–299 (0.75, 0.68–0.82)<br>300–374 (0.72, 0.65–0.79)<br>375–449 (0.71, 0.63–0.79)<br>450–599 (0.62, 0.55–0.69)<br>≥600 (0.63, 0.56–0.71)
Lissner, 1996[65]	The Gothenburg Prospective Study of Women; Sweden; N= 1405	100% women; 38–60 years	(adulthood PA average)<br><br>PA assessed at age 20-38 years, 39-60 years, and in 1968-69; 20-year follow-up from 1968-69	Leisure-time PA assessed via questionnaire; assessed in intensity and duration (adulthood activity index created)	Swedish National Death Registry and the State Person and Address Registry	NA (continuous PA)	Cox regression	Age, smoking, waist–hip ratio, peak expiratory flow, BP and serum triglycerides	**All-cause mortality (N=147):**<br>Per 1 unit increase in average adulthood activity index (0.42, 0.24-0.74)<br><br>**CVD mortality (N=22):**<br>Per 1 unit increase in average adulthood activity index (0.36, 0.08-1.59)
Martinez-Gomez, 2022[109]	Taiwan MJ cohort; China; N=210327	50.6% women; 18+ years	(up to 19-year cumulative average PA)<br><br>Average PA from at least 2 surveys between 1997 and 2016; up to 23-year follow-up to 2020	Leisure-time PA assessed via questionnaire; assessed in MET.h/week	Linkage to Taiwan's National Death File registry	(1) 'none' (0 MET hours/week), (2) 'insufficient' (0.01–7.49 MET hours/week), (3) 'recommended' (7.50–15.00 MET hours/week) and (4) 'additional' (>15.00 MET hours/week)	Cox regression adjust for time-varying covariates	Sex, educational attainment, marital status, smoking, alcohol consumption, regular meal patterns, occupational PA, BMI, cancer, CVD, hypertension, dyslipidaemia, diabetes and electrocardiogram	**All-cause mortality (N=10539):**<br>None (ref)<br>Insufficient (0.74, 0.69-0.80)<br>Recommended (0.64, 0.60-0.70)<br>Additional (0.59, 0.54-0.64)<br><br>**CVD mortality (N=1919):**<br>None (ref)<br>Insufficient (0.68, 0.58-0.80)<br>Recommended (0.56, 0.47-0.67)<br>Additional (0.56, 0.47-0.68)
Orsini, 2009[110]	Population-based cohort of Swedish men; Sweden; N= 45,887	0% women; 45-79 years	PA measured at baseline, 30- and 50-years age; followed up from 1998 to 2007	Lifetime average total PA (MET.h/day); work/occupational activity level; leisure-time walking or bicycling (min/day); measured by questionnaires	Swedish National Cancer Register and the Regional Cancer Register	Total PA divided into four quartiles; walking or bicycling as hardly ever, <20min/day, 20-40 min/day, 41-60 min/day and >60 min/day	Cox regression	Baseline age, waist-to-hip ratio, height, diabetes, alcohol consumption, smoking status, years of education, total energy intake, consumption of dairy product and red meat and parental history with respect to prostate cancer	(leisure-time walking or bicycling)<br><br>**Fatal prostate cancer (N=190):**<br>Hardly ever (1.81, 0.64–5.12)<br><20min/day (1.21, 0.74–1.97)<br>20-40 min/day (ref)<br>41-60 min/day (0.90, 0.56–1.46)<br>>60 min/day (0.72, 0.44–1.18)

Stenholm, 2016[111]	InCHIANTI study; Italy; N= 1,149	56% women; ≥ 65 years	(up to 45-year PA)<br><br>PA measured in 3 time points (20-40 years, 40-60 years, and baseline); followed up from 1998-2000 to 2010	Total PA assessed by questionnaires; cumulative physical activity score derived	Mortality General Registry and death certificates	Inactive (0); moderately active (1); physically active (2)	Cox regression	Age, sex, education, smoking, BMI, alcohol consumption, coronary heart disease, stroke, peripheral arterial disease, diabetes, lung disease, knee or hip osteoarthritis, depressive symptoms, and cancer	**All-cause mortality (N=471):**<br>Score 5-6 (most active) (ref)<br>Score 3-4 (1.44, 0.97-2.14)<br>Score 1-2 (1.47, 0.95-2.26)<br>Score 0 (1.86, 1.12-3.09)
**Other**									
Hirvensalo, 2000[112]	Evergreen Project; Finland; N= 1109	65% women; 65–84 years	(PA across life span)<br><br>PA assessed at baseline (1988), recalled at age 10-19, 20-39, 40-64 years; 8-year follow-up from 1988	Total PA assessed via questionnaire; frequency and intensity of PA assessed	Official register of the Province of Central Finland and from hospital records	(1) Intact mobility and physically active (Mobile-Active), (2) Intact mobility and sedentary (Mobile-Sedentary)	Cox regression adjusted for past PA	Age, gender, marital status, education, presence of asthma, diabetes, neurological diabetes, stroke, mental diseases, CVD, musculoskeletal diseases, smoking and past PA	**All-cause mortality (N=389):**<br>Men:<br>Mobile-active (ref)<br>Mobile-sedentary (0.92, 0.53-1.59)<br><br>Women:<br>Mobile-active (ref)<br>Mobile-sedentary (0.87, 0.55-1.40)
Sherman, 1999[113]	Framingham Heart Study; USA; N= 2372	59% women; 38–70 years	(up to 15-year PA interval)<br><br>PA assessed in 1956-58 and 1969-73; 16-year follow-up from 1969-73	Total PA assessed via questionnaire; duration and intensity of PA assessed to calculate a PA score	FHS, medical and hospitalization records	NA (continuous PA score)	Cox regression for distant and recent PA (independent analyses)	Baseline levels of age, systolic blood pressure, total cholesterol, cigarettes/day, Metropolitan weight, glucose intolerance, left ventricular hypertrophy, chronic obstructive pulmonary disease, and cancer.	**All-cause mortality (N=N.A.):**<br>Men: per 1 unit increase in distant PA (0.99, 0.93-1.07); per 1 unit increase in recent PA (0.85, 0.77-0.94)<br>Women: per 1 unit increase in distant PA (1.01, 0.96-1.07); per 1 unit increase in recent PA (0.84, 0.76-0.93)<br><br>**CVD mortality (N=N.A.):**<br>Men: per 1 unit increase in distant PA (0.92, 0.81-1.01); per 1 unit increase in recent PA (0.87, 0.72-1.07)<br>Women: per 1 unit increase in distant PA (1.01, 0.90-1.11); per 1 unit increase in recent PA (1.08, 0.93-1.29)

Supplementary Table 4. Studies chosen from the same cohort for meta-analyses.

Study Cohort	Study chosen	Sample size	Follow-up period	Outcome for analyses
British Regional Heart Study	Aggio, 2020	3,231	20-year PA trajectory; median follow-up of 16.4 years	All-cause mortality<br>CVD mortality
Copenhagen City Heart Study	Schnohr, 2017	12,314	PA change between at least 2 examinations; up to 33 years follow-up	All-cause mortality<br>CVD mortality
HUNT study	Moholdt, 2020	32,811	Up to 18 years follow-up from 1995-97	All-cause mortality<br>CVD mortality
Aerobics Centre Longitudinal Study	Nauman, 2021	17,613	Change in PAI from 1974 to 2002; median follow-up of 9.3 years	All-cause mortality<br>CVD mortality
National Health Insurance Service	Lee, 2022	6,572,984	PA from 2009 to 2011; median 7.4-year follow-up	All-cause mortality
	Choi, 2022	286,402	PA from 2009-11 to 2010-12; median follow-up of 4.3 years	CVD mortality
Australian Diabetes, Obesity and Lifestyle study	Yang, 2021	6,377	PA change between 2004-2005 to 2011-2012; mean follow-up 13 years from 2004-2005	All-cause mortality
NIH-AARP Diet and Health Study	Saint-Maurice, 2019	315,059	PA change between age 15–18, 19–29, 35–39, 40–61 (baseline); mean follow-up 13.6 years from baseline	All-cause mortality<br>CVD mortality<br>Cancer mortality
Taiwan MJ cohort	Ahmadi, 2022	116,228	PA change between 1998-2013 and repeated measures 4.6 years apart; followed up for mortality for 11.9 years	All-cause mortality<br>CVD mortality<br>Cancer mortality
Nurses' Health Study	Hu, 2004	116,564	PA measured from 1980 to 1998; up to 20 years of follow-up	All-cause mortality<br>CVD mortality<br>Cancer mortality

Supplementary Table 5. E-values of associations.

	All-cause mortality		CVD mortality		Cancer mortality	
	**RR (95%CI)**	**E-value (CI)**	**RR (95%CI)**	**E-value (CI)**	**RR (95%CI)**	**E-value (CI)**
**Total PA**						
**PA trajectory**						
Consistently inactive	Reference		Reference		Reference	
Consistently active	0.71 (0.67, 0.76)	2.12 (1.96)	0.61 (0.44, 0.84)	2.66 (1.67)	0.80 (0.66, 0.96)	1.81 (1.25)
Increasing PA	0.78 (0.75, 0.82)	1.88 (1.74)	0.33 (0.06, 1.84)	NA	0.81 (0.60, 1.10)	NA
Decreasing PA	0.97 (0.86, 1.08)	NA	0.46 (0.14, 1.45)	NA	0.88 (0.75, 1.04)	NA
**Time-varying PA**						
Inactive	Reference		Reference		Reference	
Active	0.61 (0.50, 0.74)	2.66 (2.04)	0.54 (0.38, 0.77)	3.11 (1.92)	0.72 (0.48, 1.09)	NA
**Cumulative PA**						
Inactive	Reference		Reference		Reference	
Active	0.71 (0.62, 0.81)	2.17 (1.77)	0.57 (0.51, 0.65)	2.91 (2.45)	0.81 (0.73, 0.89)	1.77 (1.50)
**Leisure-time PA**						
**PA trajectory**						
Consistently inactive	Reference		Reference			
Consistently active	0.61 (0.55, 0.67)	2.55 (2.26)	0.62 (0.53, 0.72)	2.61 (2.12)	NA	NA
Increasing PA	0.74 (0.66, 0.84)	2.08 (1.70)	0.70 (0.60, 0.82)	2.21 (1.74)	NA	NA
Decreasing PA	0.95 (0.92, 0.99)	1.29 (1.11)	0.97 (0.91, 1.02)	NA	NA	NA
**Time-varying PA**						
Inactive	Reference					
Active	0.68 (0.60, 0.77)	2.35 (1.85)	NA	NA	NA	NA
**Cumulative PA**						
Inactive	Reference					
Active	0.64 (0.48, 0.85)	2.50 (1.63)	NA	NA	NA	NA

PA: physical activity; RR: relative risk

Supplementary Table 6. Sub-group analyses by potential sources of heterogeneity, for associations between PA trajectories and all-cause mortality.

Variables	Consistently active				Increasing PA				Decreasing PA			
	No. of studies	RR (95%CI)	I^2, $P_{heterogeneity}$	P (subgroup difference)	No. of studies	RR (95%CI)	I^2, $P_{heterogeneity}$	P (subgroup difference)	No. of studies	RR (95%CI)	I^2, $P_{heterogeneity}$	P (subgroup difference)
**All studies**	32	0.67 (0.64, 0.71)	63.8%, <0.01	-	21	0.76 (0.71, 0.81)	52.8%, <0.01	-	21	0.95 (0.89, 1.01)	58.9%, <0.01	-
**Adjustment for adiposity-related covariates**				0.16				0.25				0.34
Yes	22	0.68 (0.65, 0.72)	59.4%, <0.01		14	0.75 (0.69, 0.81)	67.7%, <0.01		15	0.93 (0.87, 1.01)	70.7%, <0.01	
No	10	0.61 (0.52, 0.71)	72.8%, <0.01		7	0.81 (0.73, 0.90)	0%, 0.84		6	0.99 (0.90, 1.09)	0%, 0.97	
**Exclusion of first few years of follow-up**				0.92				0.82				0.64
Yes	13	0.67 (0.61, 0.73)	62.5%, <0.01		9	0.77 (0.68, 0.88)	57.2%, 0.02		8	0.97 (0.89, 1.05)	23.7%, 0.24	
No	19	0.67 (0.63, 0.72)	68.0%, <0.01		12	0.76 (0.71, 0.82)	30.1%, 0.14		13	0.94 (0.86, 1.02)	63.6%, <0.01	
**Gender composition**				0.93				0.95				0.26
0-49% women	13	0.67 (0.63, 0.72)	66.8%, <0.01		9	0.76 (0.70, 0.82)	59.5%, 0.01		10	0.92 (0.83, 1.01)	77.4%, <0.01	
50-100% women	21	0.67 (0.62, 0.72)	63.5%, <0.01		13	0.75 (0.68, 0.84)	47.1%, 0.03		12	0.98 (0.92, 1.05)	0%, 0.71	
**Baseline age range**				0.03				0.03				0.05
Young-mid age (18-50 years)	2	0.74 (0.65, 0.85)	0%, 0.76		2	0.84 (0.74, 0.96)	0%, 0.45		2	1.05 (0.85, 1.30)	51.8%, 0.15	
Mid-age (30-69 years)	9	0.73 (0.67, 0.80)	55.6%, 0.02		4	0.82 (0.73, 0.92)	0%, 0.79		2	0.99 (0.89, 1.10)	0%, 0.78	
Mid-old age (40-92 years)	6	0.65 (0.56, 0.75)	73.9%, <0.01		4	0.68 (0.61, 0.76)	37.4%, 0.19		5	0.98 (0.85, 1.15)	64.0%, 0.03	
Old age (60-96 years)	8	0.61 (0.53, 0.71)	53.3%, 0.03		6	0.68 (0.57, 0.82)	43.0%, 0.10		8	0.91 (0.81, 1.02)	30.8%, 0.17	
All ages	7	0.62 (0.57, 0.68)	47.8%, 0.07		5	0.79 (0.76, 0.83)	0%, 0.84		4	0.86 (0.82, 0.90)	2.4%, 0.38	
**Number of PA categories**				0.02				0.47				0.05
2	16	0.70 (0.66, 0.75)	63.1%, <0.01		16	0.78 (0.73, 0.82)	20.7%, 0.21		12	0.99 (0.91, 1.08)	64.0%, <0.01	
≥3	16	0.62 (0.57, 0.68)	66.0%, <0.01		5	0.73 (0.62, 0.85)	64.3%, 0.02		8	0.83 (0.72, 0.97)	58.4%, 0.02	
**Trajectory years**				0.02				0.35				0.03
<7 years	11	0.61 (0.55, 0.68)	71.3%, <0.01		10	0.73 (0.65, 0.82)	50.3%, 0.03		9	0.88 (0.83, 0.93)	6.7%, 0.38	
≥7 years	20	0.70 (0.66, 0.75)	54.4%, <0.01		11	0.78 (0.72, 0.85)	48.1%, 0.03		12	0.98 (0.91, 1.06)	52.2%, 0.01	
**Follow-up years**				0.01				0.17				<0.01
<10 years	13	0.59 (0.52, 0.66)	53.3%, 0.01		10	0.70 (0.61, 0.80)	46.9%, 0.04		11	0.85 (0.81, 0.89)	1.3%, 0.43	
≥10 years	18	0.69 (0.65, 0.74)	63.3%, <0.01		11	0.78 (0.72, 0.85)	54.0%, 0.01		10	1.00 (0.96, 1.05)	11.3%, 0.34	
**Continents**				0.13				0.04				<0.01
Europe	20	0.64 (0.59, 0.70)	67.6%, <0.01		14	0.81 (0.76, 0.87)	0%, 0.60		12	0.99 (0.93, 1.06)	0%, 0.83	
North America	7	0.72 (0.66, 0.79)	55.3%, 0.04		4	0.69 (0.62, 0.77)	55.0%, 0.06		7	0.92 (0.81, 1.04)	73.9%, <0.01	
Asia	3	0.62 (0.51, 0.75)	64.5%, 0.06		2	0.71 (0.42. 1.22)	23.8%, 0.25		2	0.85 (0.81, 0.89)	0%, 0.76	
Oceania	2	0.72 (0.63, 0.81)	0%, 0.56		-	-			-	-		

**Study quality**				0.78				0.38				0.62
Good	14	0.68 (0.64, 0.71)	55.1%, 0.01		9	0.77 (0.69, 0.85)	71.6%, <0.01		9	0.94 (0.86, 1.02)	68.6%, <0.01	
Fair	8	0.67 (0.60, 0.75)	63.1%, 0.01		5	0.70 (0.60, 0.83)	66.6%, 0.02		6	0.92 (0.78, 1.09)	75.6%, <0.01	
Poor	10	0.64 (0.56, 0.73)	73.4%, <0.01		7	0.81 (0.73, 0.90)	0%, 0.84		6	0.99 (0.90, 1.09)	0%, 0.97	

PA: physical activity; RR: relative risk

Supplementary Table 7. Assessment of credibility of subgroup difference for the association between PA patterns and all-cause mortality based on ICEMAN.

Variable	Q1	Q2	Q3	Q4	Q5	Q6	Q7	Q8	Overall credibility
**Consistently active**									
Adjustment for adiposity-related covariates	Completely between	Unclear	Large	Probably no	Chance a very likely explanation	Probably no	Definitely yes	Not applicable	Low
Exclusion of first few years of follow-up	Completely between	Unclear	Large	Definitely no	Chance a very likely explanation	Probably no	Definitely yes	Not applicable	Low
Gender composition	Mostly between	Unclear	Large	Unclear	Chance a very likely explanation	Probably no	Definitely yes	Not applicable	Low
Baseline age range	Completely between	Not applicable	Very small	Probably yes	Chance a likely explanation	Probably no	Definitely yes	Not applicable	Low
Number of PA categories	Completely between	Not applicable	Large	Probably yes	Chance a likely explanation	Probably no	Definitely yes	Definitely yes	Maximum moderate
Trajectory years	Completely between	Not applicable	Large	Probably yes	Chance a likely explanation	Probably no	Definitely yes	Definitely yes	Maximum moderate
Follow-up years	Completely between	Not applicable	Large	Probably yes	Chance may not explain	Probably no	Definitely yes	Definitely yes	Maximum moderate
Continents	Completely between	Not applicable	Very small	Unclear	Chance a very likely explanation	Probably no	Definitely yes	Not applicable	Low
Study quality	Completely between	Not applicable	Rather large	Unclear	Chance a very likely explanation	Probably no	Definitely yes	Not applicable	Low
**Increasing PA**									
Adjustment for adiposity-related covariates	Completely between	Unclear	Rather large	Probably no	Chance a very likely explanation	Probably no	Definitely yes	Not applicable	Low
Exclusion of first few years of follow-up	Completely between	Unclear	Rather large	Definitely no	Chance a very likely explanation	Probably no	Definitely yes	Not applicable	Low
Gender composition	Mostly between	Unclear	Rather large	Unclear	Chance a very likely explanation	Probably no	Definitely yes	Not applicable	Low
Baseline age range	Completely between	Not applicable	Very small	Probably yes	Chance a likely explanation	Probably no	Definitely yes	Not applicable	Low
Number of PA categories	Completely between	Not applicable	Rather large	Probably no	Chance a very likely explanation	Probably no	Definitely yes	Definitely yes	Low
Trajectory years	Completely between	Not applicable	Large	Probably yes	Chance a very likely explanation	Probably no	Definitely yes	Definitely yes	Low
Follow-up years	Completely between	Not applicable	Large	Probably yes	Chance a very likely explanation	Probably no	Definitely yes	Definitely yes	Low

Continents	Completely between	Not applicable	Very small	Unclear	Chance a likely explanation	Probably no	Definitely yes	Not applicable	Low
Study quality	Completely between	Not applicable	Rather large	Unclear	Chance a very likely explanation	Probably no	Definitely yes	Not applicable	Low
**Decreasing PA**									
Adjustment for adiposity-related covariates	Completely between	Unclear	Rather large	Probably no	Chance a very likely explanation	Probably no	Definitely yes	Not applicable	Low
Exclusion of first few years of follow-up	Completely between	Unclear	Rather large	Definitely no	Chance a very likely explanation	Probably no	Definitely yes	Not applicable	Low
Gender composition	Mostly between	Unclear	Large	Unclear	Chance a very likely explanation	Probably no	Definitely yes	Not applicable	Low
Baseline age range	Completely between	Not applicable	Very small	Probably yes	Chance a likely explanation	Probably no	Definitely yes	Not applicable	Low
Number of PA categories	Completely between	Not applicable	Rather large	Probably yes	Chance a likely explanation	Probably no	Definitely yes	Definitely yes	Maximum moderate
Trajectory years	Completely between	Not applicable	Rather large	Probably yes	Chance a likely explanation	Probably no	Definitely yes	Definitely yes	Maximum moderate
Follow-up years	Completely between	Not applicable	Large	Probably yes	Chance an unlikely explanation	Probably no	Definitely yes	Definitely yes	Maximum moderate
Continents	Completely between	Not applicable	Very small	Unclear	Chance an unlikely explanation	Probably no	Definitely yes	Not applicable	Low
Study quality	Completely between	Not applicable	Rather large	Unclear	Chance a very likely explanation	Probably no	Definitely yes	Not applicable	Low

Q, question; Q1, Is the analysis of effect modification based on comparison within rather than between trials? Q2, For within-trial comparisons, is the effect modification similar from trial to trial? Q3, For between-trial comparisons, is the number of trials large? Q4, Was the direction of the effect modification correctly hypothesized priori? Q5, Does a test for interaction suggest that chance is an unlikely explanation of the apparent effect modification? Q6, Did the authors test only a small number of effect modifiers? Q7, Did the authors use a random effects model? Q8, If the effect modifier is a continuous variable, were arbitrary cut points avoided?

Supplementary Table 8. Comparison between main analyses and studies only reporting hazard ratios.

	**Consistently active**		**Increasing PA**		**Decreasing PA**		**Time-varying PA**		**Cumulative PA**	
	Total PA	Leisure PA	Total PA	Leisure PA	Total PA	Leisure PA	Total PA	Leisure PA	Total PA	Leisure PA
**Main analyses**										
No. of studies	N=17	N=15	N=11	N=10	N=10	N=11	N=6	N=5	N=2	N=3
RR (95% CI)	0.71 (0.67, 0.76)	0.61 (0.55, 0.67)	0.78 (0.75, 0.82)	0.74 (0.66, 0.84)	0.97 (0.86, 1.08)	0.95 (0.92, 0.99)	0.61 (0.50, 0.74)	0.68 (0.60, 0.77)	0.71 (0.62, 0.81)	0.64 (0.48, 0.85)
**Studies reporting hazard ratio only**										
No. of studies	N=14	N=14	N=10	N=9	N=9	N=11	N=5	N=5	N=2	N=3
RR (95% CI)	0.69 (0.64, 0.74)	0.60 (0.55, 0.67)	0.79 (0.76, 0.83)	0.74 (0.65, 0.83)	0.94 (0.84, 1.04)	0.95 (0.92, 0.99)	0.59 (0.48, 0.73)	0.68 (0.60, 0.77)	0.71 (0.62, 0.81)	0.64 (0.48, 0.85)

Supplementary Table 9. Comparison between main analyses and results excluding sample size<500 and published before 2003.

	**Consistently active**		**Increasing PA**		**Decreasing PA**		**Time-varying PA**		**Cumulative PA**	
	Total PA	Leisure PA	Total PA	Leisure PA	Total PA	Leisure PA	Total PA	Leisure PA	Total PA	Leisure PA
**Main analyses**										
No. of studies	N=17	N=15	N=11	N=10	N=10	N=11	N=6	N=5	N=2	N=3
RR (95% CI)	0.71 (0.67, 0.76)	0.61 (0.55, 0.67)	0.78 (0.75, 0.82)	0.74 (0.66, 0.84)	0.97 (0.86, 1.08)	0.95 (0.92, 0.99)	0.61 (0.50, 0.74)	0.68 (0.60, 0.77)	0.71 (0.62, 0.81)	0.64 (0.48, 0.85)
**Excluding sample size <500 and published before 2003**										
No. of studies	N= 14	N=13	N= 9	N=8	N= 7	N= 9	N=6	N=5	N=2	N=3
RR (95% CI)	0.72 (0.67, 0.76)	0.63 (0.57, 0.69)	0.78 (0.75, 0.82)	0.73 (0.65, 0.83)	0.94 (0.83, 1.06)	0.95 (0.92, 0.99)	0.61 (0.50, 0.74)	0.68 (0.60, 0.77)	0.71 (0.62, 0.81)	0.64 (0.48, 0.85)

Supplementary Table 10A. Summary of PA questionnaires on validity and ability to detect changes over time.

Study measuring PA changes	PA Questionnaire/measurements	Questionnaire tested for validity?	Does the questionnaire show ability to detect changes over time?
Aggio, 2020	PA questions in British Regional Heart Study	Y	-
Ahmadi, 2022	International Physical Activity Questionnaire (IPAQ) short form	Y	Y
Ahmadi, 2022	PA questions in Taiwan MJ cohort	Y	-
Andersen, 2004	Saltin-Grimby Physical Activity Level Scale (SGPALS)	Y	Y
Balboa-Castillo, 2011	Single PA question	-	-
Barbiellini, 2022	Hours spent each week on specific activities	-	-
Bauman, 2017	Saltin-Grimby Physical Activity Level Scale (SGPALS)	Y	Y
Bergwall, 2021	Minutes per week spent on 17 common physical activities	-	-
Breidablik, 2023	PA questions in HUNT study	Y	Y
Byberg, 2009	PA questionnaire in ULSAM study	Y	Y
Cheema, 2023	PA questions in NHANES I augmentation	-	-
Choi, 2022	International Physical Activity Questionnaire (IPAQ)	Y	Y
Coelho-Ravagnani, 2021	PA questions in Aerobics Center Longitudinal Study	Y	-
Duarte Junior, 2024	PA questionnaire from EPIC cohort study	Y	Y
Dwyer, 2015	Pedometer data	Y	Y
Gregg, 2003	Harvard Alumni Activity Survey (HAAS)	Y	-
Hassan, 2023	Baecke questionnaire	Y	-
Higueras-Fresnillo, 2017	Single PA question from the Spanish National Health Survey	-	-
Holme, 2015	Gothenburg PA questionnaire	Y	-
Hsu, 2018	Physical Activity Scale for the Elderly (PASE)	Y	-
Huang, 2021	International Physical Activity Questionnaire (IPAQ)	Y	Y
Hulsegge, 2016	PA questionnaire from EPIC cohort study	Y	Y
Jasiukaitiene, 2021	Mean length of time spent per week during leisure time in PA	Y	-
Karvinen, 2015	Single PA question on intensity categories	Y	-
Keadle, 2015	PA questions in NIH-AARP Diet and Health Study	-	-
Kieffer, 2019	PA questions in HUNT study	Y	Y
Laddu, 2018	Physical Activity Scale for the Elderly (PASE)	Y	-
Lee, 2014	PA questions in Aerobics Center Longitudinal Study	Y	-
Lee, 2022	International Physical Activity Questionnaire (IPAQ)	Y	Y
Lee, 2023	PA questions in Taiwan MJ cohort	Y	-
Lewis, 2018	Physical Activity Scale for the Elderly (PASE)	Y	-
Li, 2022	PA questions in English Longitudinal Study of Ageing (ELSA) cohort	-	-

Moholdt, 2020	PA questions in HUNT study	Y	Y
Mok, 2019	EPIC-Norfolk PA questionnaire	Y	-
Nauman, 2021	PA questions in Aerobics Center Longitudinal Study	Y	-
Nordstoga, 2019	PA questions in HUNT study	Y	Y
Petersen, 2012	Saltin-Grimby Physical Activity Level Scale (SGPALS)	Y	Y
Saint-Maurice, 2019	Risk Factor Questionnaire for past PA	-	-
Sanchez-Sanchez, 2020	Physical Activity Scale for the Elderly (PASE)	Y	-
Schnohr, 2003	Saltin-Grimby Physical Activity Level Scale (SGPALS)	Y	Y
Schnohr, 2017	Saltin-Grimby Physical Activity Level Scale (SGPALS)	Y	Y
Shaw, 2014	Index of PA in active behaviours	-	-
Shortreed, 2013	Number of daily hours spent on PA	-	-
Stamatakis, 2024	PA questions in Taiwan MJ cohort	Y	-
Talbot, 2007	Amount of time spent performing PA activities	-	-
Trolle-Lagerros, 2005	Single PA question on intensity categories	-	-
Vaes, 2014	Saltin-Grimby Physical Activity Level Scale (SGPALS)	Y	Y
Williamson, 2019	Risk Factor Prevalence Study<br>& International Physical Activity Questionnaire (IPAQ)	Y	Y
Wolin, 2010	Single PA question on intensity categories	-	-
Yang, 2021	Active Australia Survey	Y	Y
Yin, 2023	Two questions in current and past PA	-	-
Østergaard, 2018	PA questionnaire from EPIC cohort study	Y	Y
Xue, 2012	Minnesota Leisure Time Activities Questionnaire	Y	-
Aijo, 2016	PA questionnaire based on Saltin-Grimby Physical Activity Level Scale (SGPALS)	Y	Y
Bijnen, 1999	Zutphen Physical Activity Questionnaire	Y	-
Hein, 1994	The Copenhagen Male Study PA questionnaire	-	-
Johansson, 1999	Face-to-face PA interview	Y	Y
Lissner, 1996	Saltin-Grimby Physical Activity Level Scale (SGPALS)	Y	Y
Paffenbarger, 1994	Harvard Alumni Activity Survey (HAAS)	Y	-
Wannamethee, 1998	PA questions in British Regional Heart Study	Y	-

Supplementary Table 10B. Evidence for 1) validity of questionnaires (in comparison to device-based measurements) to assess overall PA volume and MVPA; and 2) validity/ability of questionnaires to detect PA change.

	**Validity of questionnaires to assess overall PA and MVPA**		**Validity/ability of questionnaires to detect PA change**		
**Questionnaires (corresponding studies)**	**Evidence**	**Correlation coefficient**	**Evidence**	**Assessments/measures**	**Results**
PA questions in HUNT study ([39 58 66 69])	10.1177/1403494807085373<br>10.1007/s10654-007-9110-9	Overall PA: ~0.48<br>MVPA: 0.30-0.46	10.1007/s00520-012-1530-8	Wilcoxon's statistic for differences between 2 assessments	Effect size 0.42 (p-value <0.001)
Saltin-Grimby Physical Activity Level Scale (SGPALS) ([32 33 36 65 72 75 76 80])<br>&<br>PA questionnaire in ULSAM study (originating from the Saltin-Grimby questionnaire) ([40])	10.1371/journal.pone.0273480	Overall PA: ~0.40<br>MVPA: ~0.35	10.1111/sms.12611<br>10.1023/A:1024682523710	Comparison between changes in PA with changes in blood lipids/serum cholesterol and BMI over 25 years	Similar patterns observed, indicating questionnaire validity remained stable over time
International Physical Activity Questionnaire (IPAQ) ([30 42 53 83 108])	10.1186/s12889-015-1785-3	Overall PA & MVPA: 0.33–0.40	10.1513/AnnalsATS.201804-265OC	Responsiveness analysis	Self-reported change in weekly MVPA was significantly different between 2 assessments (p-value = 0.004)
	10.1016/j.jsams.2024.04.016<br>10.1111/j.1753-6405.2008.00305.x	Overall PA: 0.42-0.61<br>MVPA: 0.30-0.48	10.1007/s00520-012-1530-8	Wilcoxon's statistic for differences between 2 assessments	Effect size 0.42 (p-value <0.001)
Active Australia Survey ([86])	10.1186/1479-5868-5-33<br>10.1371/journal.pone.0087085	Overall PA: ~0.29<br>MVPA: 0.30-0.36	10.1186/s12889-019-6717-1	Comparison between individual residual change scores from the Active Australia Survey and those from the ActiGraph	A correlation of 0.32 for moderate PA, 0.35 for MVPA

PA questionnaire from EPIC cohort study ([44 53 70])*			10.1186/1479-5868-5-33	Long-term (10-month) repeatability test of measured PA levels	The authors suggested that differences in self-reported PA may reflect true changes in PA levels in 10 months

*The ability of this questionnaire to detect PA changes was not strongly validated (only suggested by the authors). Exclusion of the studies using EPIC questionnaire did not significantly change the results.

Supplementary Table 10C. Comparison between main analyses and analyses using only the studies showing validity for overall PA/MVPA and ability to detect potential changes.

	**Consistently active**		**Increasing PA**		**Decreasing PA**	
	Total	Leisure	Total	Leisure	Total	Leisure
**Main results**						
No. of studies	N=17	N=15	N=11	N=10	N=10	N=11
RR (95% CI)	0.71 (0.67, 0.76)	0.61 (0.55, 0.67)	0.78 (0.75, 0.82)	0.74 (0.66, 0.84)	0.97 (0.86, 1.08)	0.95 (0.92, 0.99)
**Results using validated questionnaires showing sensitivity to changes**						
No. of studies	N= 6	N= 6	N= 5	N= 3	N= 4	N= 3
RR (95% CI)	0.68 (0.61, 0.75)	0.61 (0.53, 0.70)	0.79 (0.75, 0.83)	0.83 (0.72, 0.95)	0.90 (0.81, 1.00)	0.98 (0.86, 1.12)
**Results using validated questionnaires showing sensitivity to changes***						
No. of studies	N= 5	N= 6	N= 3	N= 3	N= 3	N= 3
RR (95% CI)	0.64 (0.56, 0.73)	0.61 (0.53, 0.70)	0.79 (0.75, 0.83)	0.83 (0.72, 0.95)	0.85 (0.81, 0.90) ^	0.98 (0.86, 1.12)

*Further excluding studies using EPIC questionnaire.

^Results largely due to Lee et al. (2022).

Reference

29. Aggio D, Papachristou E, Papacosta O, et al. Trajectories of physical activity from midlife to old age and associations with subsequent cardiovascular disease and all-cause mortality. *J Epidemiol Community Health* 2020;74(2):130-36. doi: 10.1136/jech-2019-212706 [published Online First: 20191108]
30. Ahmadi MN, Gill JMR, Stamatakis E. Association of Changes in Physical Activity and Adiposity With Mortality and Incidence of Cardiovascular Disease: Longitudinal Findings From the UK Biobank. *Mayo Clin Proc* 2022;97(5):847-61. doi: 10.1016/j.mayocp.2021.11.026 [published Online First: 20220409]
31. Ahmadi MN, Lee IM, Hamer M, et al. Changes in physical activity and adiposity with all-cause, cardiovascular disease, and cancer mortality. *Int J Obes (Lond)* 2022;46(10):1849-58. doi: 10.1038/s41366-022-01195-z [published Online First: 20220801]
32. Aijo M, Kauppinen M, Kujala UM, Parkatti T. Physical activity, fitness, and all-cause mortality: An 18-year follow-up among old people. *J Sport Health Sci* 2016;5(4):437-42. doi: 10.1016/j.jshs.2015.09.008 [published Online First: 20150925]
33. Andersen LB. Relative risk of mortality in the physically inactive is underestimated because of real changes in exposure level during follow-up. *Am J Epidemiol* 2004;160(2):189-95. doi: 10.1093/aje/kwh195
34. Balboa-Castillo T, Guallar-Castillon P, Leon-Munoz LM, et al. Physical activity and mortality related to obesity and functional status in older adults in Spain. *Am J Prev Med* 2011;40(1):39-46. doi: 10.1016/j.amepre.2010.10.005
35. Barbiellini Amidei C, Trevisan C, Dotto M, et al. Association of physical activity trajectories with major cardiovascular diseases in elderly people. *Heart* 2022;108(5):360-66. doi: 10.1136/heartjnl-2021-320013 [published Online First: 20220214]
36. Bauman AE, Grunseit AC, Rangul V, Heitmann BL. Physical activity, obesity and mortality: does pattern of physical activity have stronger epidemiological associations? *BMC Public Health* 2017;17(1):788. doi: 10.1186/s12889-017-4806-6 [published Online First: 20171005]
37. Bergwall S, Acosta S, Ramne S, et al. Leisure-time physical activities and the risk of cardiovascular mortality in the Malmo diet and Cancer study. *BMC Public Health* 2021;21(1):1948. doi: 10.1186/s12889-021-11972-6 [published Online First: 20211026]
38. Bijnen FC, Feskens EJ, Caspersen CJ, et al. Baseline and previous physical activity in relation to mortality in elderly men: the Zutphen Elderly Study. *Am J Epidemiol* 1999;150(12):1289-96. doi: 10.1093/oxfordjournals.aje.a009960
39. Breidablik HJ, Hufthammer KO, Rangul V, et al. Lower levels of physical activity volume are beneficial, and it's never too late to start: Results from the HUNT Study, Norway. *Scand J Public Health* 2024;52(4):476-85. doi: 10.1177/14034948231162729 [published Online First: 20230324]
40. Byberg L, Melhus H, Gedeborg R, et al. Total mortality after changes in leisure time physical activity in 50 year old men: 35 year follow-up of population based cohort. *BMJ* 2009;338:b688. doi: 10.1136/bmj.b688 [published Online First: 20090305]
41. Cheema BS, Shi Z, White RL, Atlantis E. Associations of Recreational and Nonrecreational Physical Activity and Body Weight Change on Cardiovascular Disease Mortality During the Obesogenic Transition in the United States: National Health and Nutrition Examination Survey Follow-up Study. *J Phys Act Health* 2023;20(10):971-79. doi: 10.1123/jpah.2022-0624 [published Online First: 20230718]
42. Choi Y, Choi JW. Changes in the Frequency of Moderate-to-Vigorous Physical Activity and Subsequent Risk of All-Cause and Cardiovascular Disease Mortality. *Int J Environ Res Public Health* 2022;19(1) doi: 10.3390/ijerph19010504 [published Online First: 20220103]
43. Coelho-Ravagnani CF, Almeida JA, Sui X, et al. Changes in Compliance With Physical Activity Guidelines and Cardiovascular Disease Mortality. *J Phys Act Health* 2021;18(6):638-43. doi: 10.1123/jpah.2020-0740 [published Online First: 20210409]
44. Duarte Junior MA, Martinez-Gomez D, Pintos-Carrillo S, et al. Associations of physical activity type, volume, intensity, and changes over time with all-cause mortality in older adults: The Seniors-ENRICA cohorts. *Scand J Med Sci Sports* 2024;34(1):e14536. doi: 10.1111/sms.14536 [published Online First: 20231115]
45. Dwyer T, Pezic A, Sun C, et al. Objectively Measured Daily Steps and Subsequent Long Term All-Cause Mortality: The Tasped Prospective Cohort Study. *PLoS One* 2015;10(11):e0141274. doi: 10.1371/journal.pone.0141274 [published Online First: 20151104]
46. Gregg EW, Cauley JA, Stone K, et al. Relationship of changes in physical activity and mortality among older women. *JAMA* 2003;289(18):2379-86. doi: 10.1001/jama.289.18.2379

47. Hassan L, Huhndorf P, Mikolajczyk R, Kluttig A. Physical activity trajectories at older age and all-cause mortality: A cohort study. *PLoS One* 2023;18(1):e0280878. doi: 10.1371/journal.pone.0280878 [published Online First: 20230126]
48. Hein HO, Suadicanl P, Sørensen H, Gyntelberg F. Changes in physical activity level and risk of ischaemic heart disease. *Scandinavian Journal of Medicine & Science in Sports* 1994;4(1):57-64. doi: https://doi.org/10.1111/j.1600-0838.1994.tb00406.x
49. Higueras-Fresnillo S, Guallar-Castillon P, Cabanas-Sanchez V, et al. Changes in physical activity and cardiovascular mortality in older adults. *J Geriatr Cardiol* 2017;14(4):280-81. doi: 10.11909/j.issn.1671-5411.2017.04.009
50. Holme I, Anderssen SA. Increases in physical activity is as important as smoking cessation for reduction in total mortality in elderly men: 12 years of follow-up of the Oslo II study. *Br J Sports Med* 2015;49(11):743-8. doi: 10.1136/bjsports-2014-094522
51. Hsu B, Merom D, Blyth FM, et al. Total Physical Activity, Exercise Intensity, and Walking Speed as Predictors of All-Cause and Cause-Specific Mortality Over 7 Years in Older Men: The Concord Health and Aging in Men Project. *J Am Med Dir Assoc* 2018;19(3):216-22. doi: 10.1016/j.jamda.2017.08.018 [published Online First: 20171006]
52. Huang Y, Jiang C, Xu L, et al. Mortality in relation to changes in physical activity in middle-aged to older Chinese: An 8-year follow-up of the Guangzhou Biobank Cohort Study. *J Sport Health Sci* 2021;10(4):430-38. doi: 10.1016/j.jshs.2020.08.007 [published Online First: 20200820]
53. Hulsegge G, Looman M, Smit HA, et al. Lifestyle Changes in Young Adulthood and Middle Age and Risk of Cardiovascular Disease and All-Cause Mortality: The Doetinchem Cohort Study. *J Am Heart Assoc* 2016;5(1) doi: 10.1161/JAHA.115.002432 [published Online First: 20160113]
54. Jasiukaitiene V, Luksiene D, Kranciukaite-Butylkiniene D, Tamosiunas A. Changes in physical activity and mortality risk among an adult Lithuanian urban population: results from a cohort study. *Public Health* 2021;191:3-10. doi: 10.1016/j.puhe.2020.11.024 [published Online First: 20210115]
55. Johansson SE, Sundquist J. Change in lifestyle factors and their influence on health status and all-cause mortality. *Int J Epidemiol* 1999;28(6):1073-80. doi: 10.1093/ije/28.6.1073
56. Karvinen S, Waller K, Silvennoinen M, et al. Physical activity in adulthood: genes and mortality. *Sci Rep* 2015;5:18259. doi: 10.1038/srep18259 [published Online First: 20151215]
57. Keadle SK, Arem H, Moore SC, et al. Impact of changes in television viewing time and physical activity on longevity: a prospective cohort study. *Int J Behav Nutr Phys Act* 2015;12:156. doi: 10.1186/s12966-015-0315-0 [published Online First: 20151218]
58. Kieffer SK, Croci I, Wisloff U, Nauman J. Temporal Changes in a Novel Metric of Physical Activity Tracking (Personal Activity Intelligence) and Mortality: The HUNT Study, Norway. *Prog Cardiovasc Dis* 2019;62(2):186-92. doi: 10.1016/j.pcad.2018.09.002 [published Online First: 20180915]
59. Laddu D, Parimi N, Cauley JA, et al. The Association Between Trajectories of Physical Activity and All-Cause and Cause-Specific Mortality. *J Gerontol A Biol Sci Med Sci* 2018;73(12):1708-13. doi: 10.1093/gerona/gly037
60. Lee CH, Han KD, Kim DH, Kwak MS. Continuing regular physical activity and maintaining body weight have a synergistic interaction in improving survival: a population-based cohort study including 6.5 million people. *Eur J Prev Cardiol* 2022;29(3):547-55. doi: 10.1093/eurjpc/zwab190
61. Lee CL, Liu WJ, Chen CH, Wang JS. Associations of Long-Term Physical Activity Trajectories With All-Cause Mortality in a General Population. *Int J Public Health* 2023;68:1605332. doi: 10.3389/ijph.2023.1605332 [published Online First: 20230116]
62. Lee DC, Pate RR, Lavie CJ, et al. Leisure-time running reduces all-cause and cardiovascular mortality risk. *J Am Coll Cardiol* 2014;64(5):472-81. doi: 10.1016/j.jacc.2014.04.058
63. Lewis ZH, Markides KS, Ottenbacher KJ, Al Snih S. The Impact of 10-Year Physical Activity Changes on 7-Year Mortality in Older Mexican Americans. *J Phys Act Health* 2018;15(1):30-39. doi: 10.1123/jpah.2016-0454 [published Online First: 20170922]
64. Li C, Ma Y, Hua R, et al. Long-term physical activity participation trajectories were associated with subsequent cognitive decline, risk of dementia and all-cause mortality among adults aged >/=50 years: a population-based cohort study. *Age Ageing* 2022;51(3) doi: 10.1093/ageing/afac071
65. Lissner L, Bengtsson C, Bjorkelund C, Wedel H. Physical activity levels and changes in relation to longevity. A prospective study of Swedish women. *Am J Epidemiol* 1996;143(1):54-62. doi: 10.1093/oxfordjournals.aje.a008657

66. Moholdt T, Skarpsno ES, Moe B, Nilsen TIL. It is never too late to start: adherence to physical activity recommendations for 11-22 years and risk of all-cause and cardiovascular disease mortality. The HUNT Study. *Br J Sports Med* 2020 doi: 10.1136/bjsports-2020-102350 [published Online First: 20200928]
67. Mok A, Khaw KT, Luben R, et al. Physical activity trajectories and mortality: population based cohort study. *BMJ* 2019;365:l2323. doi: 10.1136/bmj.l2323 [published Online First: 20190626]
68. Nauman J, Arena R, Zisko N, et al. Temporal changes in personal activity intelligence and mortality: Data from the aerobics center longitudinal study. *Prog Cardiovasc Dis* 2021;64:127-34. doi: 10.1016/j.pcad.2020.12.001 [published Online First: 20201225]
69. Nordstoga AL, Zotcheva E, Svedahl ER, et al. Long-term changes in body weight and physical activity in relation to all-cause and cardiovascular mortality: the HUNT study. *Int J Behav Nutr Phys Act* 2019;16(1):45. doi: 10.1186/s12966-019-0809-2 [published Online First: 20190520]
70. Ostergaard L, Jensen MK, Overvad K, et al. Associations Between Changes in Cycling and All-Cause Mortality Risk. *Am J Prev Med* 2018;55(5):615-23. doi: 10.1016/j.amepre.2018.06.009
71. Paffenbarger RS, Jr., Kampert JB, Lee IM, et al. Changes in physical activity and other lifeway patterns influencing longevity. *Med Sci Sports Exerc* 1994;26(7):857-65.
72. Petersen CB, Gronbaek M, Helge JW, et al. Changes in physical activity in leisure time and the risk of myocardial infarction, ischemic heart disease, and all-cause mortality. *Eur J Epidemiol* 2012;27(2):91-9. doi: 10.1007/s10654-012-9656-z [published Online First: 20120205]
73. Saint-Maurice PF, Coughlan D, Kelly SP, et al. Association of Leisure-Time Physical Activity Across the Adult Life Course With All-Cause and Cause-Specific Mortality. *JAMA Netw Open* 2019;2(3):e190355. doi: 10.1001/jamanetworkopen.2019.0355 [published Online First: 20190301]
74. Sanchez-Sanchez JL, Izquierdo M, Carnicero-Carreno JA, et al. Physical activity trajectories, mortality, hospitalization, and disability in the Toledo Study of Healthy Aging. *J Cachexia Sarcopenia Muscle* 2020;11(4):1007-17. doi: 10.1002/jcsm.12566 [published Online First: 20200312]
75. Schnohr P, O'Keefe JH, Lange P, et al. Impact of persistence and non-persistence in leisure time physical activity on coronary heart disease and all-cause mortality: The Copenhagen City Heart Study. *Eur J Prev Cardiol* 2017;24(15):1615-23. doi: 10.1177/2047487317721021 [published Online First: 20170721]
76. Schnohr P, Scharling H, Jensen JS. Changes in leisure-time physical activity and risk of death: an observational study of 7,000 men and women. *Am J Epidemiol* 2003;158(7):639-44. doi: 10.1093/aje/kwg207
77. Shaw BA, Agahi N. Smoking and physical inactivity patterns during midlife as predictors of all-cause mortality and disability: A 39-year prospective study. *Eur J Ageing* 2014;11(3):195-204. doi: 10.1007/s10433-013-0298-0
78. Shortreed SM, Peeters A, Forbes AB. Estimating the effect of long-term physical activity on cardiovascular disease and mortality: evidence from the Framingham Heart Study. *Heart* 2013;99(9):649-54. doi: 10.1136/heartjnl-2012-303461 [published Online First: 20130308]
79. Stamatakis E, Ahmadi MN, Elphick TL, et al. Occupational physical activity, all-cause, cardiovascular disease, and cancer mortality in 349,248 adults: Prospective and longitudinal analyses of the MJ Cohort. *J Sport Health Sci* 2024;13(4):579-89. doi: 10.1016/j.jshs.2024.03.002 [published Online First: 20240308]
80. Trolle-Lagerros Y, Mucci LA, Kumle M, et al. Physical activity as a determinant of mortality in women. *Epidemiology* 2005;16(6):780-5. doi: 10.1097/01.ede.0000181312.35964.22
81. Vaes AW, Garcia-Aymerich J, Marott JL, et al. Changes in physical activity and all-cause mortality in COPD. *Eur Respir J* 2014;44(5):1199-209. doi: 10.1183/09031936.00023214 [published Online First: 20140725]
82. Wannamethee SG, Shaper AG, Walker M. Changes in physical activity, mortality, and incidence of coronary heart disease in older men. *Lancet* 1998;351(9116):1603-8. doi: 10.1016/S0140-6736(97)12355-8
83. Williamson EJ, Polak J, Simpson JA, et al. Sustained adherence to a Mediterranean diet and physical activity on all-cause mortality in the Melbourne Collaborative Cohort Study: application of the g-formula. *BMC Public Health* 2019;19(1):1733. doi: 10.1186/s12889-019-7919-2 [published Online First: 20191226]
84. Wolin KY, Patel AV, Campbell PT, et al. Change in physical activity and colon cancer incidence and mortality. *Cancer Epidemiol Biomarkers Prev* 2010;19(12):3000-4. doi: 10.1158/1055-9965.EPI-10-0764 [published Online First: 20101026]
85. Xue QL, Bandeen-Roche K, Mielenz TJ, et al. Patterns of 12-year change in physical activity levels in community-dwelling older women: can modest levels of physical activity help older women live longer? *Am J Epidemiol* 2012;176(6):534-43. doi: 10.1093/aje/kws125 [published Online First: 20120830]

86. Yang YI, Hodge AM, Dugue PA, et al. Mortality Effects of Hypothetical Interventions on Physical Activity and TV Viewing. *Med Sci Sports Exerc* 2021;53(2):316-23. doi: 10.1249/MSS.0000000000002479
87. Yin R, Wang Y, Li Y, et al. Changes in physical activity and all-cause mortality in the oldest old population: Findings from the Chinese Longitudinal Healthy Longevity Survey (CLHLS). *Prev Med* 2023;175:107721. doi: 10.1016/j.ypmed.2023.107721 [published Online First: 20231004]
88. Hamer M, de Oliveira C, Demakakos P. Non-exercise physical activity and survival: English longitudinal study of ageing. *Am J Prev Med* 2014;47(4):452-60. doi: 10.1016/j.amepre.2014.05.044 [published Online First: 20140718]
89. Joseph G, Marott JL, Torp-Pedersen C, et al. Dose-Response Association Between Level of Physical Activity and Mortality in Normal, Elevated, and High Blood Pressure. *Hypertension* 2019;74(6):1307-15. doi: 10.1161/HYPERTENSIONAHA.119.13786 [published Online First: 20191014]
90. Kaplan GA, Strawbridge WJ, Cohen RD, Hungerford LR. Natural history of leisure-time physical activity and its correlates: associations with mortality from all causes and cardiovascular disease over 28 years. *Am J Epidemiol* 1996;144(8):793-7. doi: 10.1093/oxfordjournals.aje.a009003
91. Lantz PM, Golberstein E, House JS, Morenoff J. Socioeconomic and behavioral risk factors for mortality in a national 19-year prospective study of U.S. adults. *Soc Sci Med* 2010;70(10):1558-66. doi: 10.1016/j.socscimed.2010.02.003 [published Online First: 20100220]
92. Lee IM, Sesso HD, Oguma Y, Paffenbarger RS, Jr. Physical activity, body weight, and pancreatic cancer mortality. *Br J Cancer* 2003;88(5):679-83. doi: 10.1038/sj.bjc.6600782
93. Lee IM, Sesso HD, Oguma Y, Paffenbarger RS, Jr. The "weekend warrior" and risk of mortality. *Am J Epidemiol* 2004;160(7):636-41. doi: 10.1093/aje/kwh274
94. O'Donovan G, Petermann-Rocha F, Ferrari G, et al. Associations of the 'weekend warrior' physical activity pattern with all-cause, cardiovascular disease and cancer mortality: the Mexico City Prospective Study. *Br J Sports Med* 2024;58(7):359-65. doi: 10.1136/bjsports-2023-107612 [published Online First: 20240321]
95. Opdal IM, Larsen LS, Hopstock LA, et al. A prospective study on the effect of self-reported health and leisure time physical activity on mortality among an ageing population: results from the Tromso study. *BMC Public Health* 2020;20(1):575. doi: 10.1186/s12889-020-08681-x [published Online First: 20200428]
96. Patterson R, Panter J, Vamos EP, et al. Associations between commute mode and cardiovascular disease, cancer, and all-cause mortality, and cancer incidence, using linked Census data over 25 years in England and Wales: a cohort study. *Lancet Planet Health* 2020;4(5):e186-e94. doi: 10.1016/S2542-5196(20)30079-6
97. Reinikainen J, Laatikainen T, Karvanen J, Tolonen H. Lifetime cumulative risk factors predict cardiovascular disease mortality in a 50-year follow-up study in Finland. *Int J Epidemiol* 2015;44(1):108-16. doi: 10.1093/ije/dyu235 [published Online First: 20141212]
98. Sabia S, Dugravot A, Kivimaki M, et al. Effect of intensity and type of physical activity on mortality: results from the Whitehall II cohort study. *Am J Public Health* 2012;102(4):698-704. doi: 10.2105/AJPH.2011.300257 [published Online First: 20111128]
99. Sheehy S, Palmer JR, Rosenberg L. Leisure Time Physical Activity in Relation to Mortality Among African American Women. *Am J Prev Med* 2020;59(5):704-13. doi: 10.1016/j.amepre.2020.05.013 [published Online First: 20200902]
100. Stessman J, Hammerman-Rozenberg R, Cohen A, et al. Physical activity, function, and longevity among the very old. *Arch Intern Med* 2009;169(16):1476-83. doi: 10.1001/archinternmed.2009.248
101. Talbot LA, Morrell CH, Fleg JL, Metter EJ. Changes in leisure time physical activity and risk of all-cause mortality in men and women: the Baltimore Longitudinal Study of Aging. *Prev Med* 2007;45(2-3):169-76. doi: 10.1016/j.ypmed.2007.05.014 [published Online First: 20070602]
102. Bembom O, van der Laan M, Haight T, Tager I. Leisure-time physical activity and all-cause mortality in an elderly cohort. *Epidemiology* 2009;20(3):424-30. doi: 10.1097/EDE.0b013e31819e3f28
103. Emberson JR, Whincup PH, Morris RW, et al. Lifestyle and cardiovascular disease in middle-aged British men: the effect of adjusting for within-person variation. *Eur Heart J* 2005;26(17):1774-82. doi: 10.1093/eurheartj/ehi224 [published Online First: 20050408]
104. Fontana D, Ceron R, d'Errico A. Occupational physical activity, all-cause mortality and incidence of cardiovascular diseases: results from three Italian cohorts. *Int Arch Occup Environ Health* 2024;97(1):81-100. doi: 10.1007/s00420-023-02028-w [published Online First: 20231215]
105. Holtermann A, Marott JL, Gyntelberg F, et al. Occupational and leisure time physical activity: risk of all-cause mortality and myocardial infarction in the Copenhagen City Heart Study. A prospective cohort study. *BMJ Open* 2012;2(1):e000556. doi: 10.1136/bmjopen-2011-000556 [published Online First: 20120213]

106. Hu FB, Willett WC, Li T, et al. Adiposity as compared with physical activity in predicting mortality among women. *N Engl J Med* 2004;351(26):2694-703. doi: 10.1056/NEJMoa042135
107. Huerta JM, Chirlaque MD, Tormo MJ, et al. Work, household, and leisure-time physical activity and risk of mortality in the EPIC-Spain cohort. *Prev Med* 2016;85:106-12. doi: 10.1016/j.ypmed.2016.02.009 [published Online First: 20160206]
108. Lee DH, Rezende LFM, Joh HK, et al. Long-Term Leisure-Time Physical Activity Intensity and All-Cause and Cause-Specific Mortality: A Prospective Cohort of US Adults. *Circulation* 2022;146(7):523-34. doi: 10.1161/CIRCULATIONAHA.121.058162 [published Online First: 20220725]
109. Martinez-Gomez D, Cabanas-Sanchez V, Yu T, et al. Long-term leisure-time physical activity and risk of all-cause and cardiovascular mortality: dose-response associations in a prospective cohort study of 210 327 Taiwanese adults. *Br J Sports Med* 2022;56(16):919-26. doi: 10.1136/bjsports-2021-104961 [published Online First: 20220406]
110. Orsini N, Bellocco R, Bottai M, et al. A prospective study of lifetime physical activity and prostate cancer incidence and mortality. *Br J Cancer* 2009;101(11):1932-8. doi: 10.1038/sj.bjc.6605404 [published Online First: 20091027]
111. Stenholm S, Koster A, Valkeinen H, et al. Association of Physical Activity History With Physical Function and Mortality in Old Age. *J Gerontol A Biol Sci Med Sci* 2016;71(4):496-501. doi: 10.1093/gerona/glv111 [published Online First: 20150818]
112. Hirvensalo M, Rantanen T, Heikkinen E. Mobility difficulties and physical activity as predictors of mortality and loss of independence in the community-living older population. *J Am Geriatr Soc* 2000;48(5):493-8. doi: 10.1111/j.1532-5415.2000.tb04994.x
113. Sherman SE, D'Agostino RB, Silbershatz H, Kannel WB. Comparison of past versus recent physical activity in the prevention of premature death and coronary artery disease. *Am Heart J* 1999;138(5 Pt 1):900-7. doi: 10.1016/s0002-8703(99)70015-3
