# Supplementary material for: Physical activity trajectories and accumulation over adulthood and their associations with all-cause and cause-specific mortality: a systematic review and meta-analysis
Source: Br J Sports Med. 2025 Jul 10;59(17):e109122. doi: 10.1136/bjsports-2024-109122 (PMC12418563; doi:10.1136/bjsports-2024-109122)
Supplement: online supplemental file 2 [file bjsports-59-17-s002.pdf]

Supplementary Figures

Supplementary Figure 1. Directed acyclic graph for longitudinal physical activity exposure and mortality outcome. Minimal adjustment sets for estimating the association between PA_0, PA_1, and mortality include sociodemographic factor, obesity_0, health_0, and lifestyle_0. 2

Supplementary Figure 2. Physical activity exposure harmonization diagram. Marginal METs: energy expenditure above resting metabolic rate.................. 3

Supplementary Figure 3. Contours for new study impact (consistent, increasing, and decreasing PA with all-cause mortality). 4

Supplementary Figure 4. Meta-analysis of studies determining the association between physical activity trajectories and cardiovascular disease (CVD) mortality... 5

Supplementary Figure 5. Meta-analysis of studies determining the association between physical activity trajectories and cancer mortality. 6

Supplementary Figure 6. Meta-analysis of studies determining the association between time-varying and cumulative physical activity and cardiovascular diseases (CVD) mortality. 7

Supplementary Figure 7. Meta-analysis of studies determining the association between time-varying and cumulative physical activity and cancer mortality.8

Supplementary Figure 8. Dose-response associations between time-varying and cumulative physical activity with all-cause mortality. 9

Supplementary Figure 9. Dose-response associations between consistently active, increasing, and decreasing physical activity with cardiovascular diseases (CVD) mortality. 10

Supplementary Figure 10. Consistent, increasing, decreasing PA patterns with all-cause mortality using harmonised exposure for low (≤4.4 mMET.h/week) versus high (≥8.75 mMET.h/week). 11

Supplementary Figure 11. Time-varying and cumulative PA with all-cause mortality using harmonised exposure for low (≤4.4 mMET.h/week) versus high (≥8.75 mMET.h/week). 12

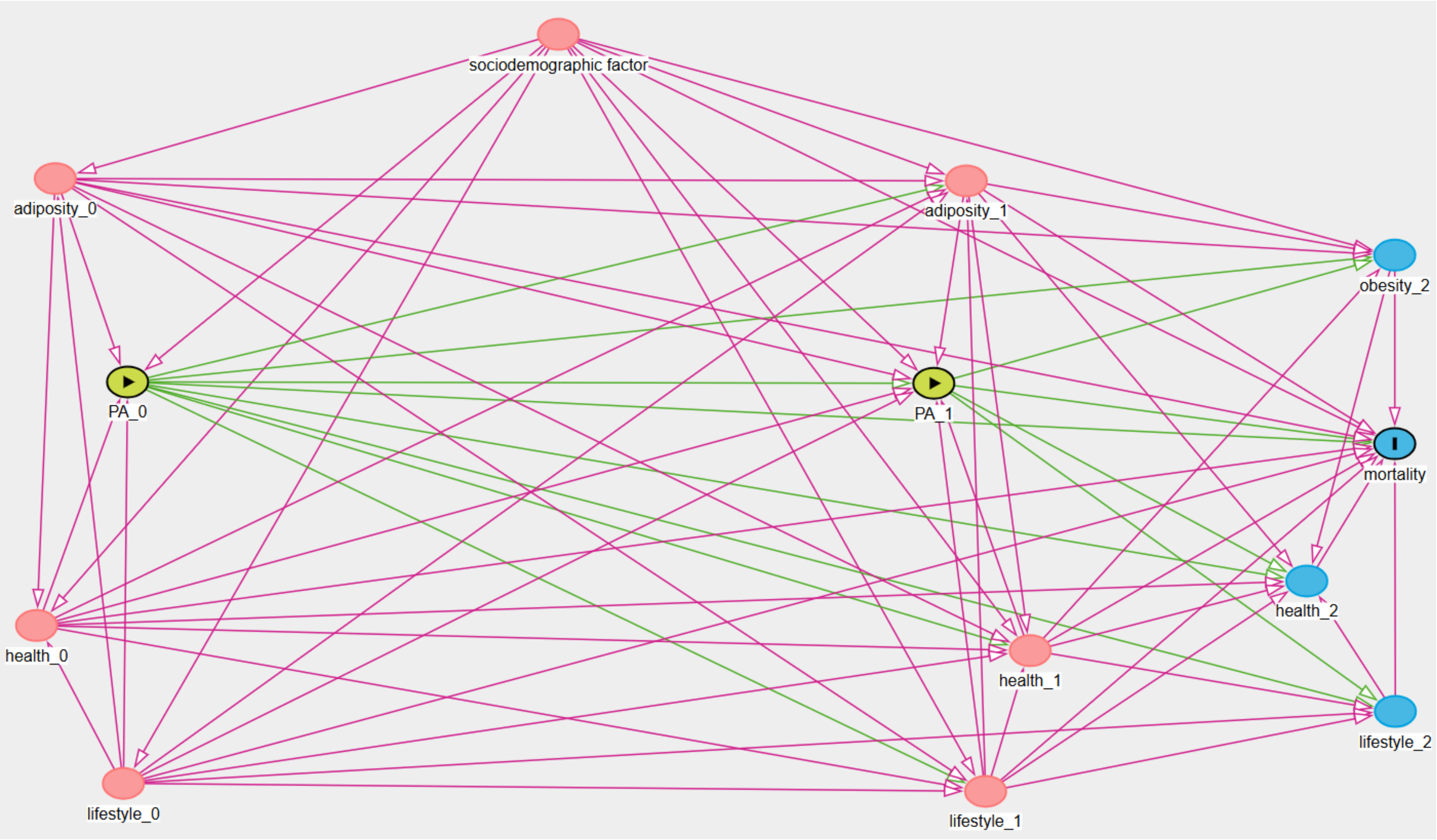

Supplementary Figure 1. Directed acyclic graph for longitudinal physical activity exposure and mortality outcome. Minimal adjustment sets for estimating the association between PA_0, PA_1, and mortality include sociodemographic factor, obesity_0, health_0, and lifestyle_0.

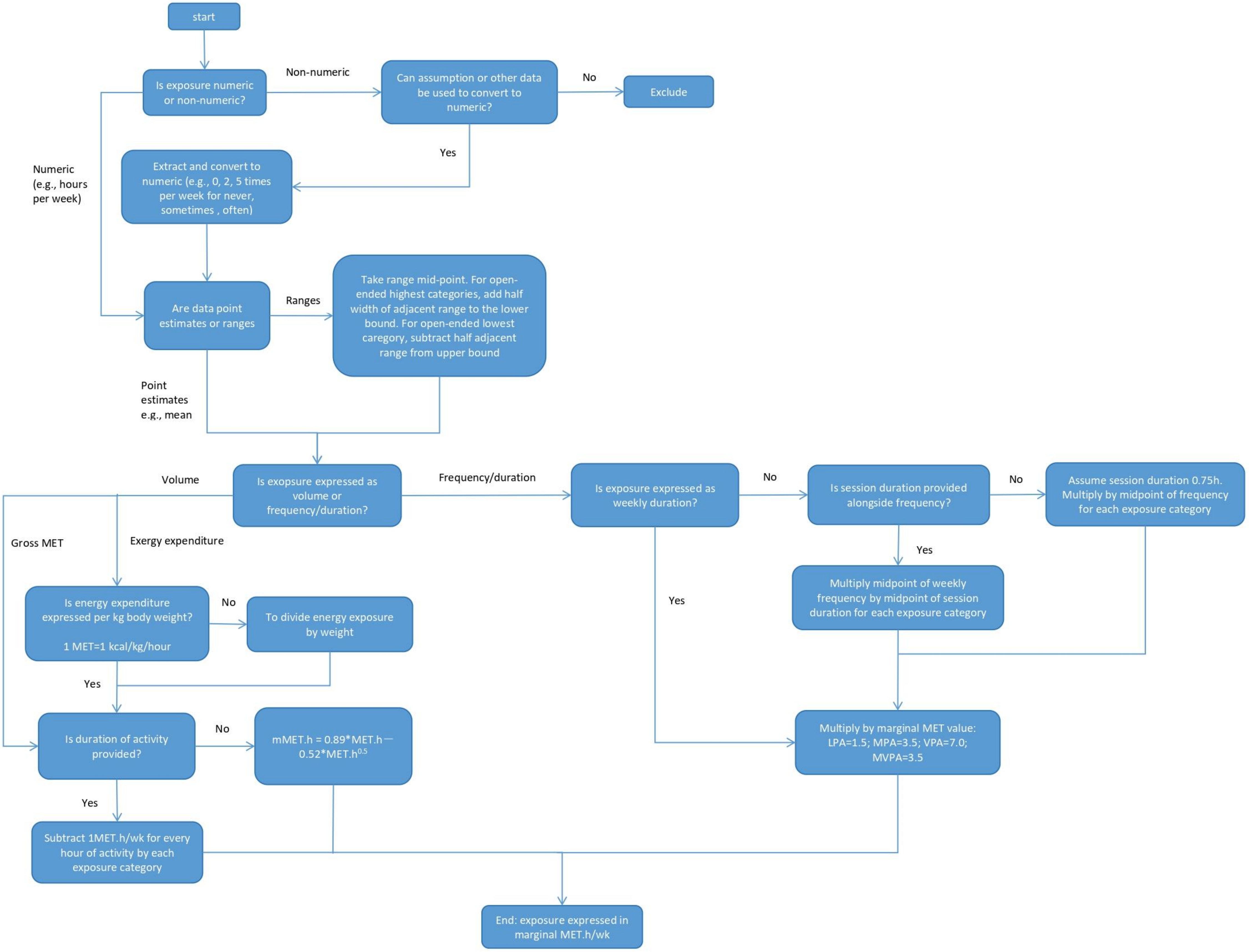

Supplementary Figure 2. Physical activity exposure harmonization diagram. Marginal METs: energy expenditure above resting metabolic rate.

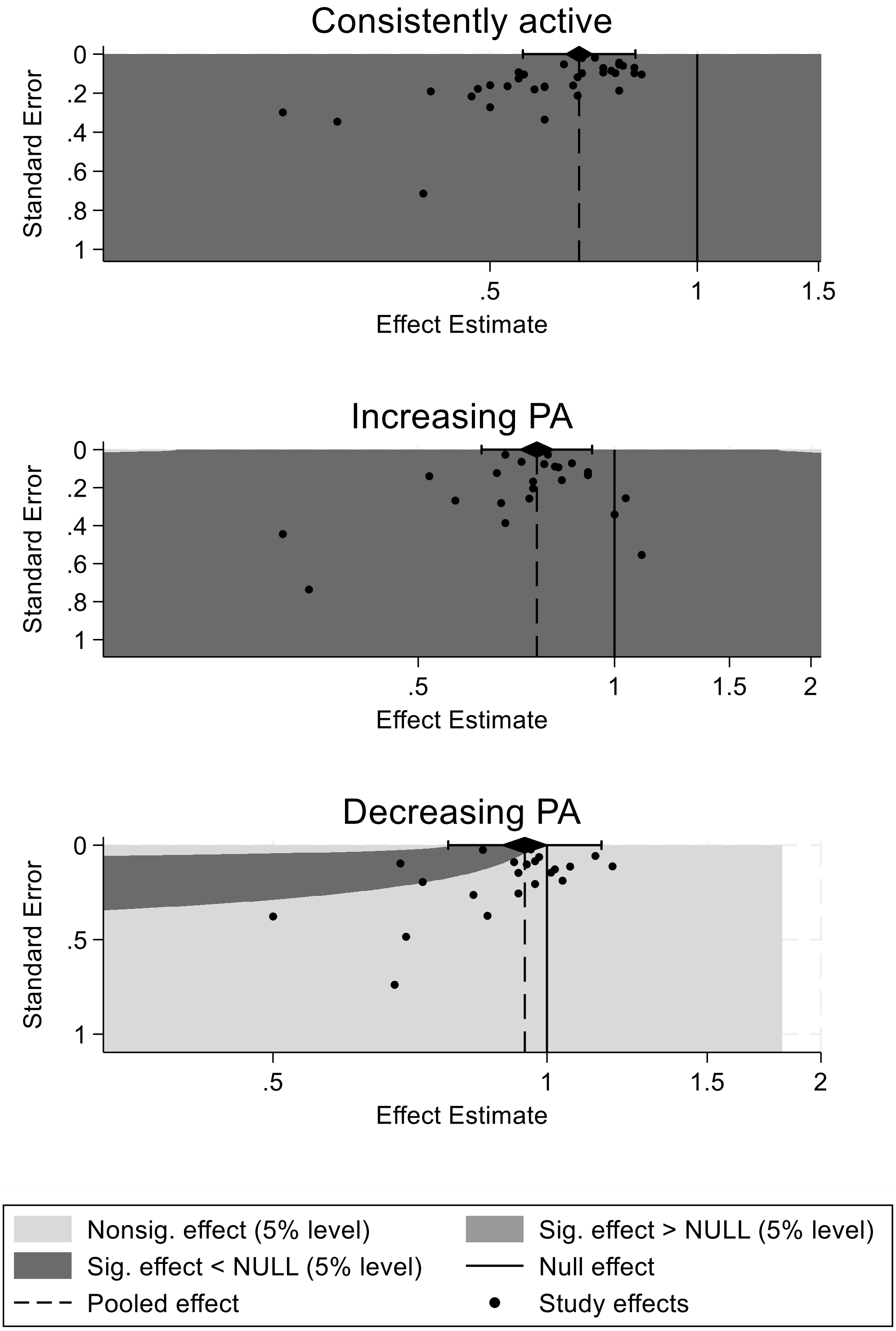

Supplementary Figure 3. Contours for new study impact (consistent, increasing, and decreasing PA with all-cause mortality).

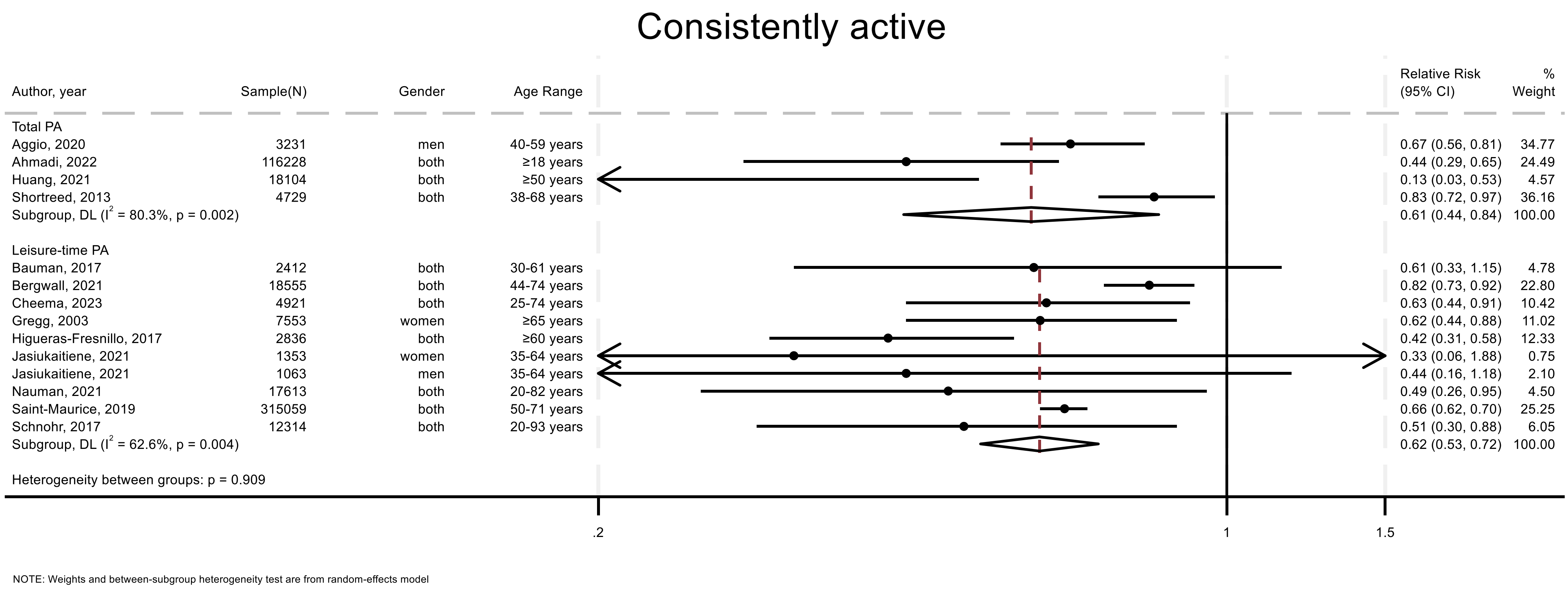

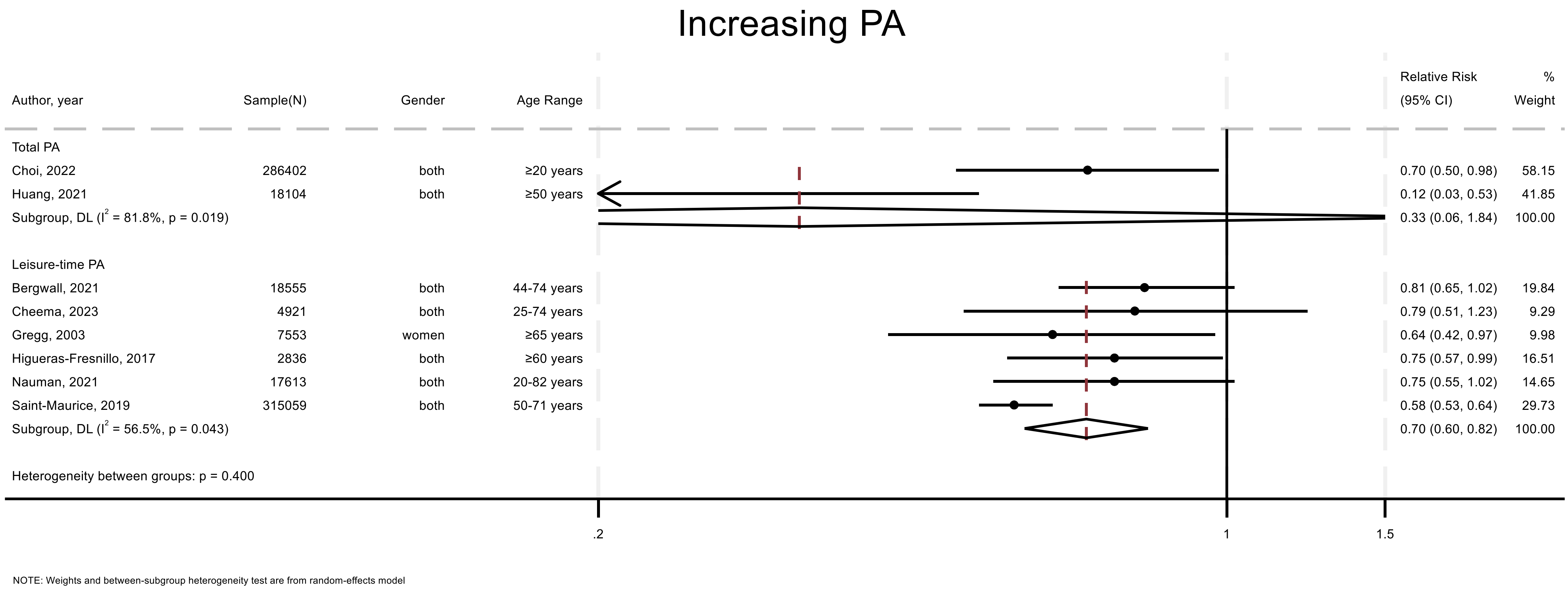

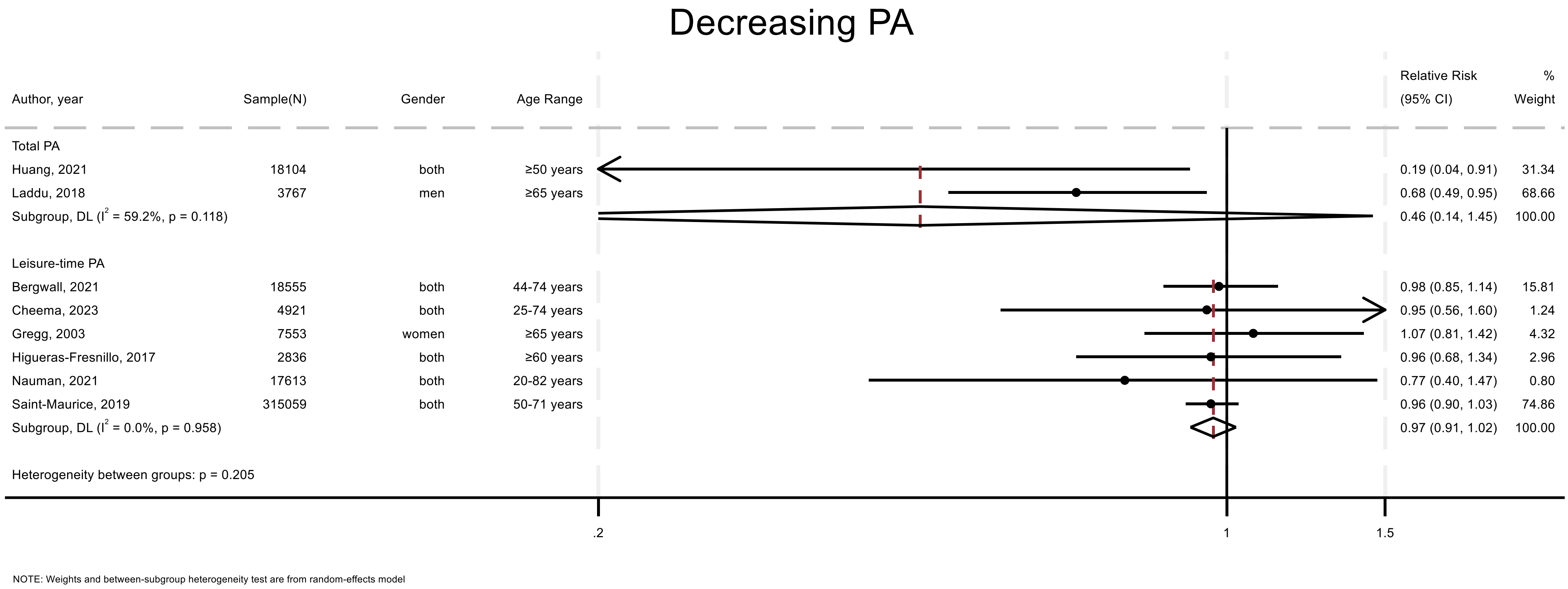

CI: confidence interval; DL: DerSimonian-Laird random-effects model

Supplementary Figure 4. Meta-analysis of studies determining the association between physical activity trajectories and cardiovascular disease (CVD) mortality.

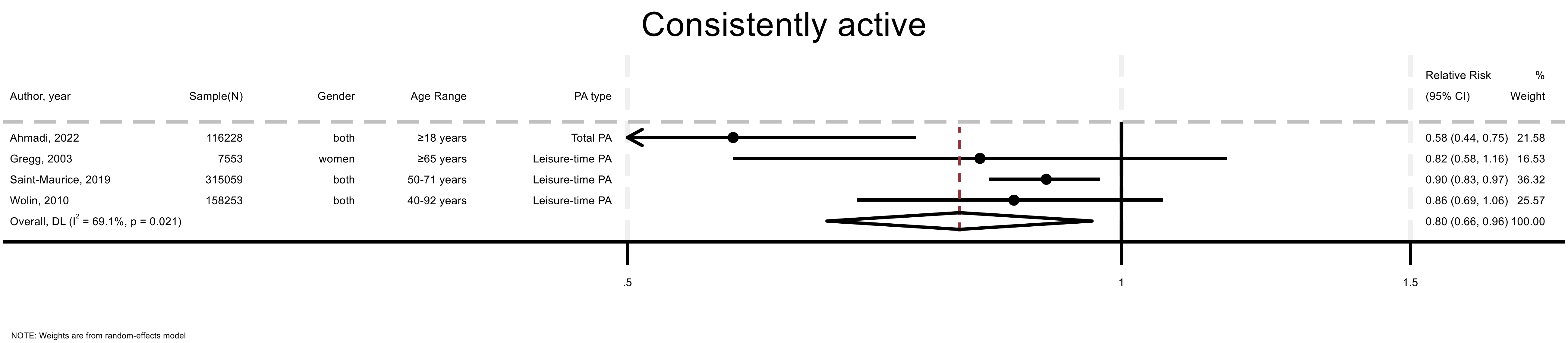

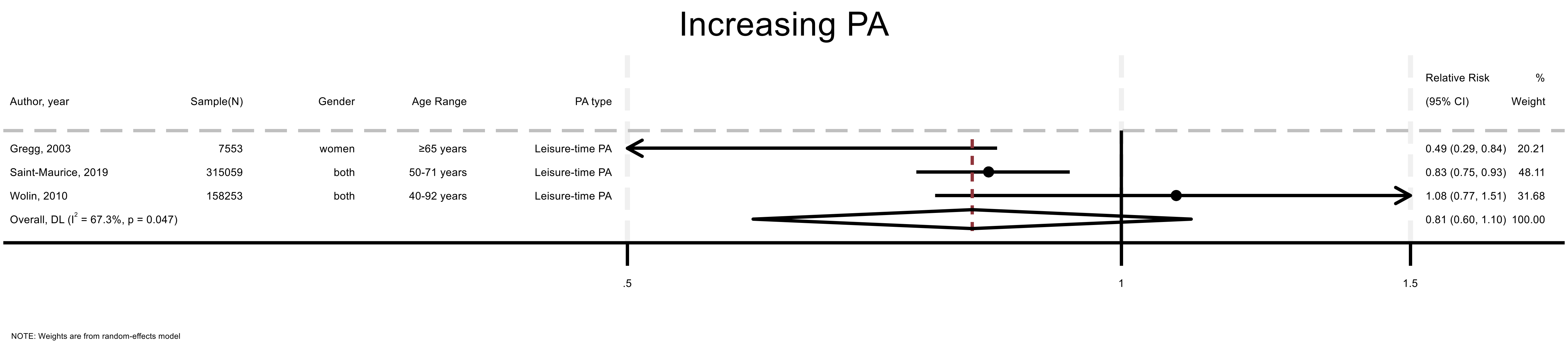

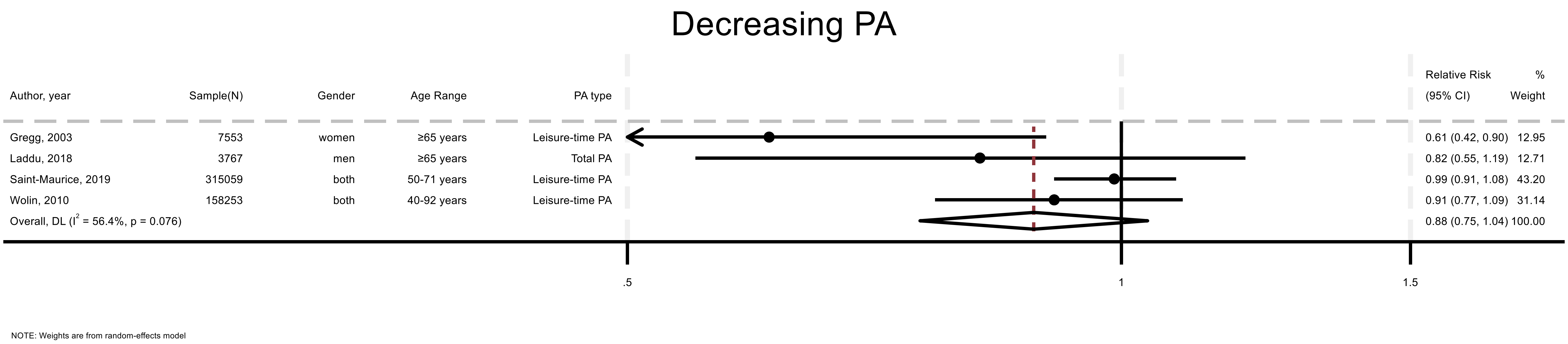

CI: confidence interval; DL: DerSimonian-Laird random-effects model

Supplementary Figure 5. Meta-analysis of studies determining the association between physical activity trajectories and cancer mortality.

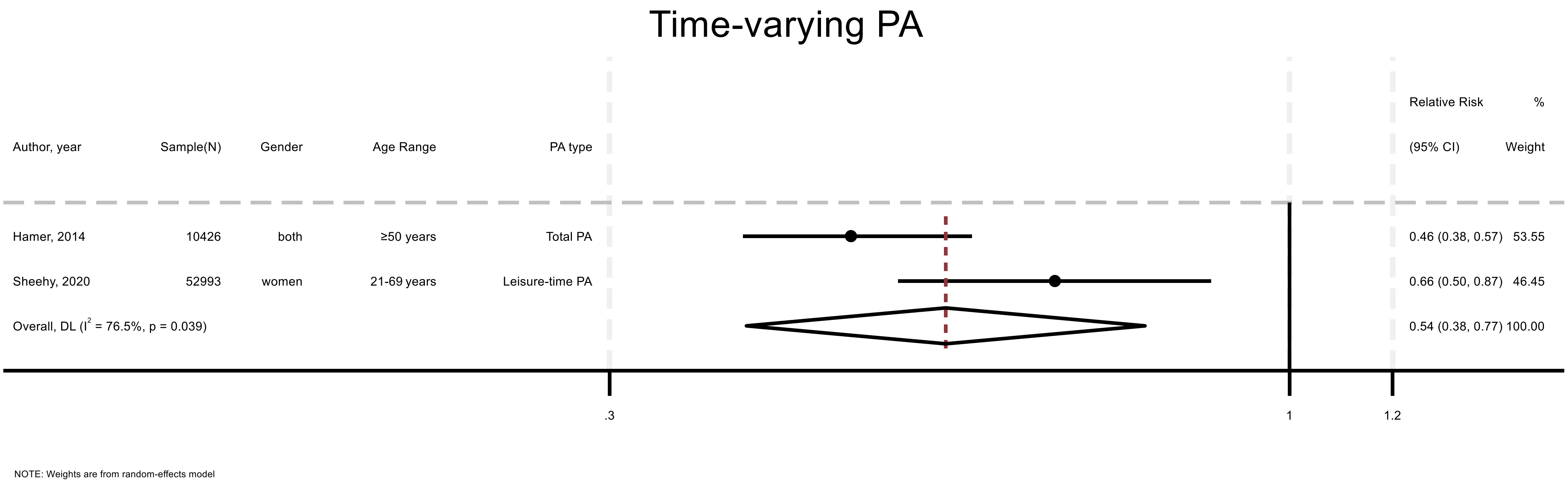

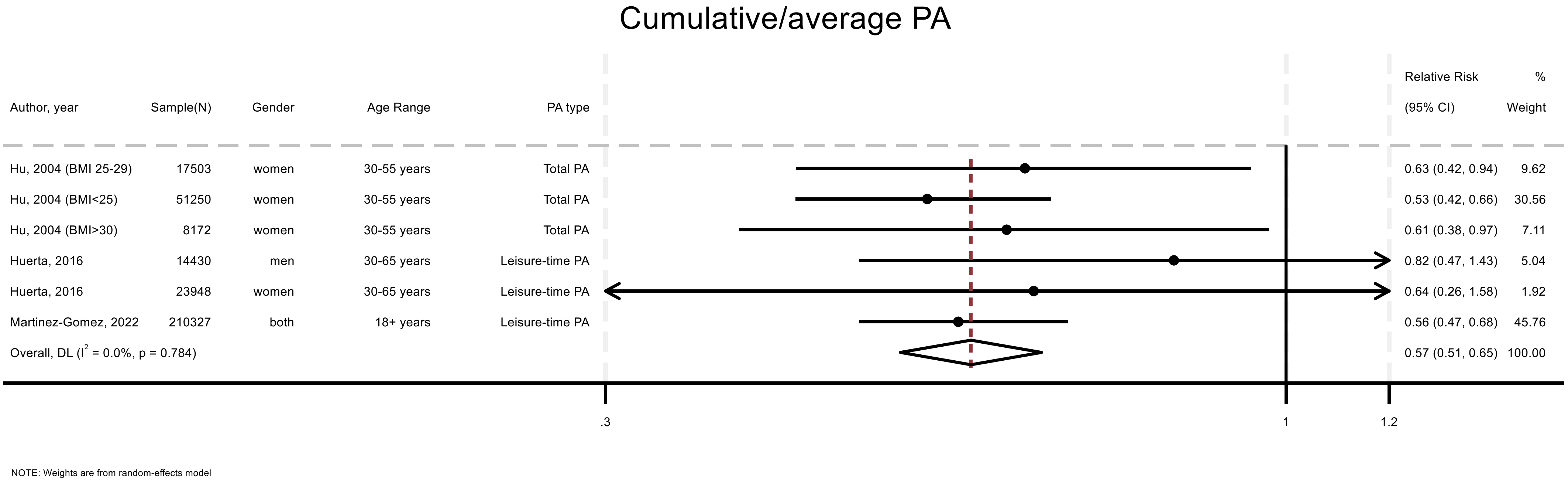

CI: confidence interval; DL: DerSimonian-Laird random-effects model

Supplementary Figure 6. Meta-analysis of studies determining the association between time-varying and cumulative physical activity and cardiovascular diseases (CVD) mortality.

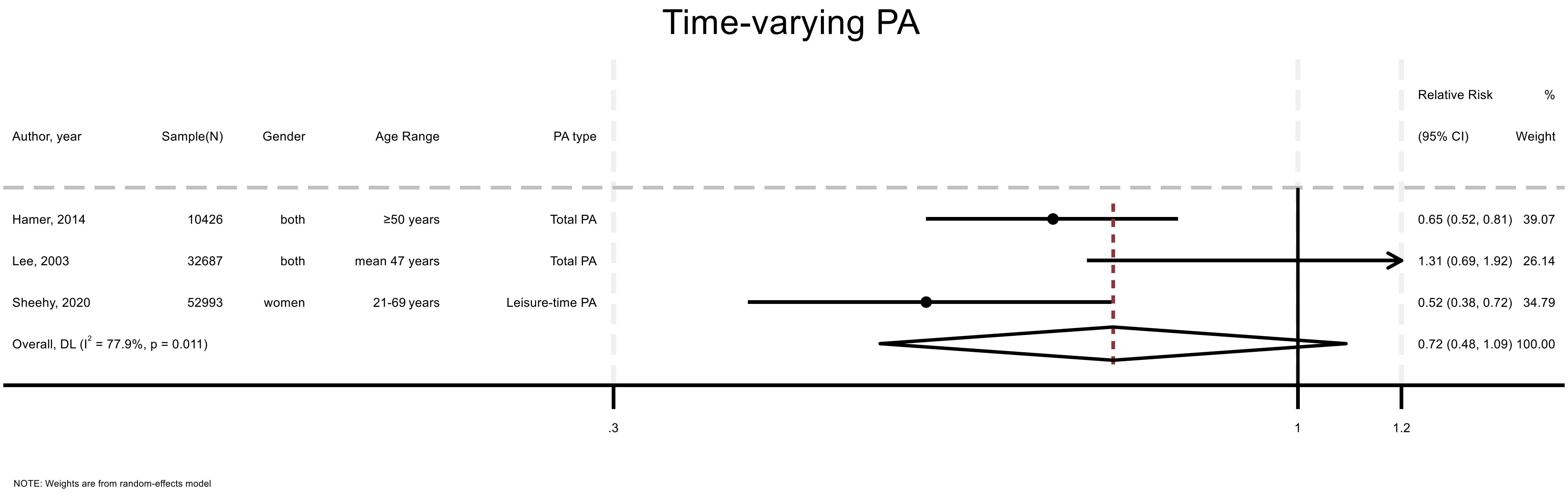

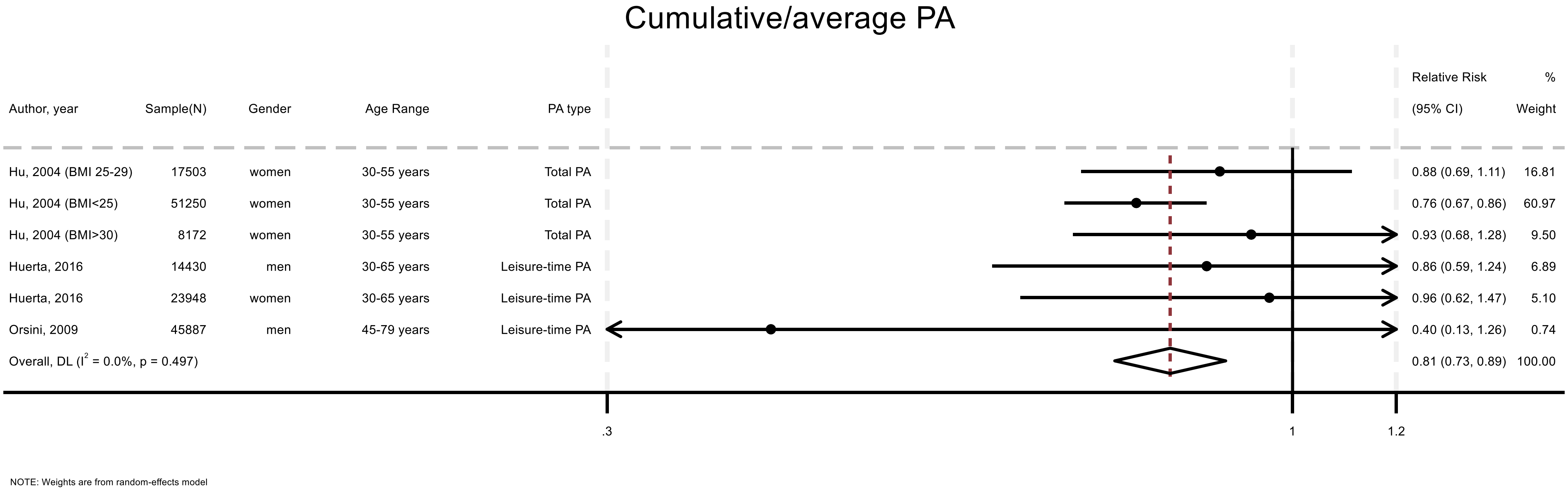

CI: confidence interval; DL: DerSimonian-Laird random-effects model

Supplementary Figure 7. Meta-analysis of studies determining the association between time-varying and cumulative physical activity and cancer mortality.

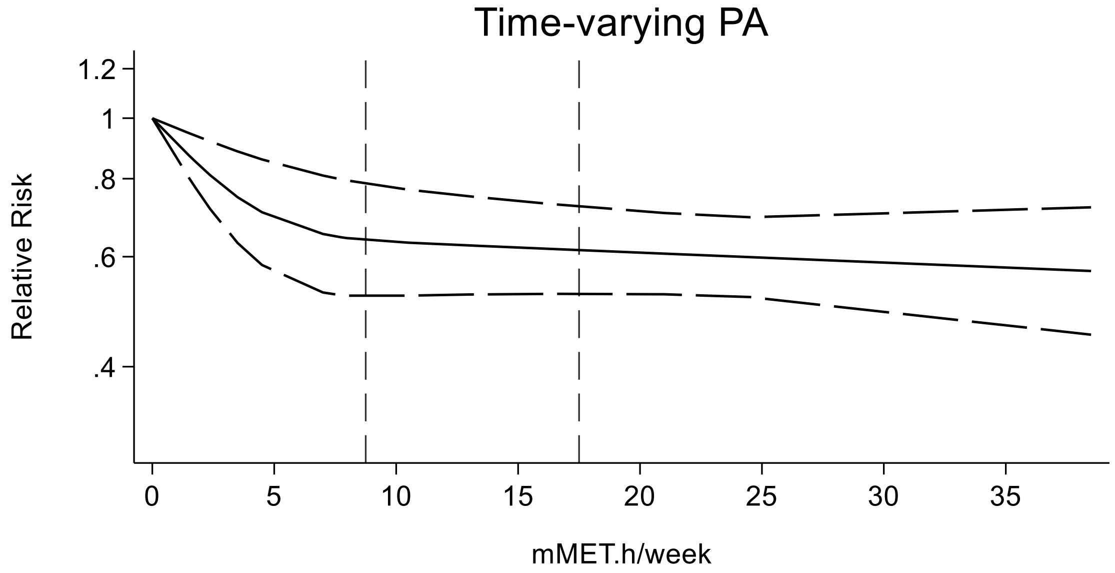

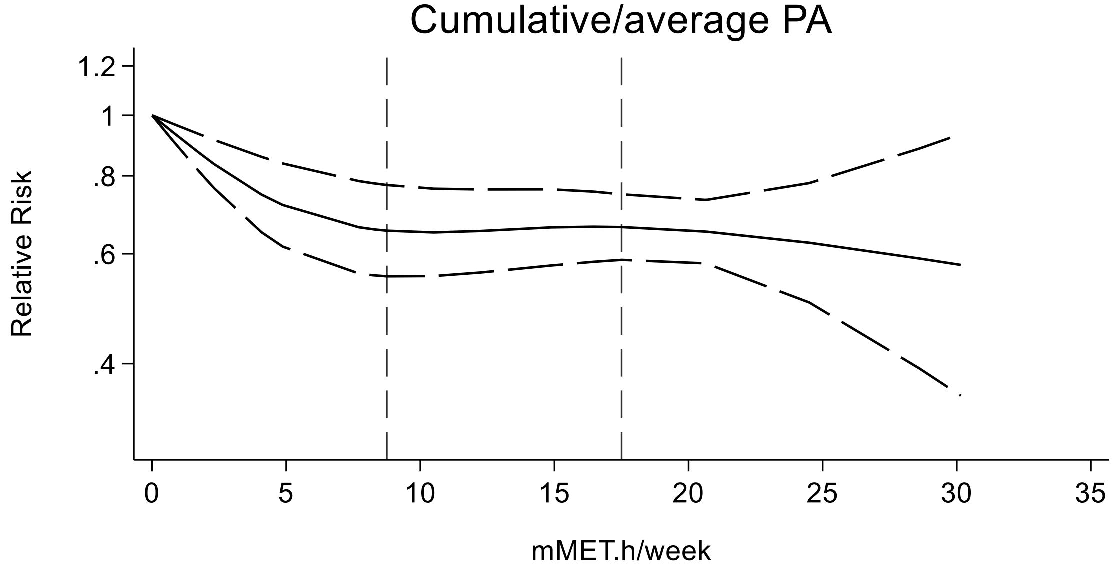

Supplementary Figure 8. Dose-response associations between time-varying and cumulative physical activity with all-cause mortality.

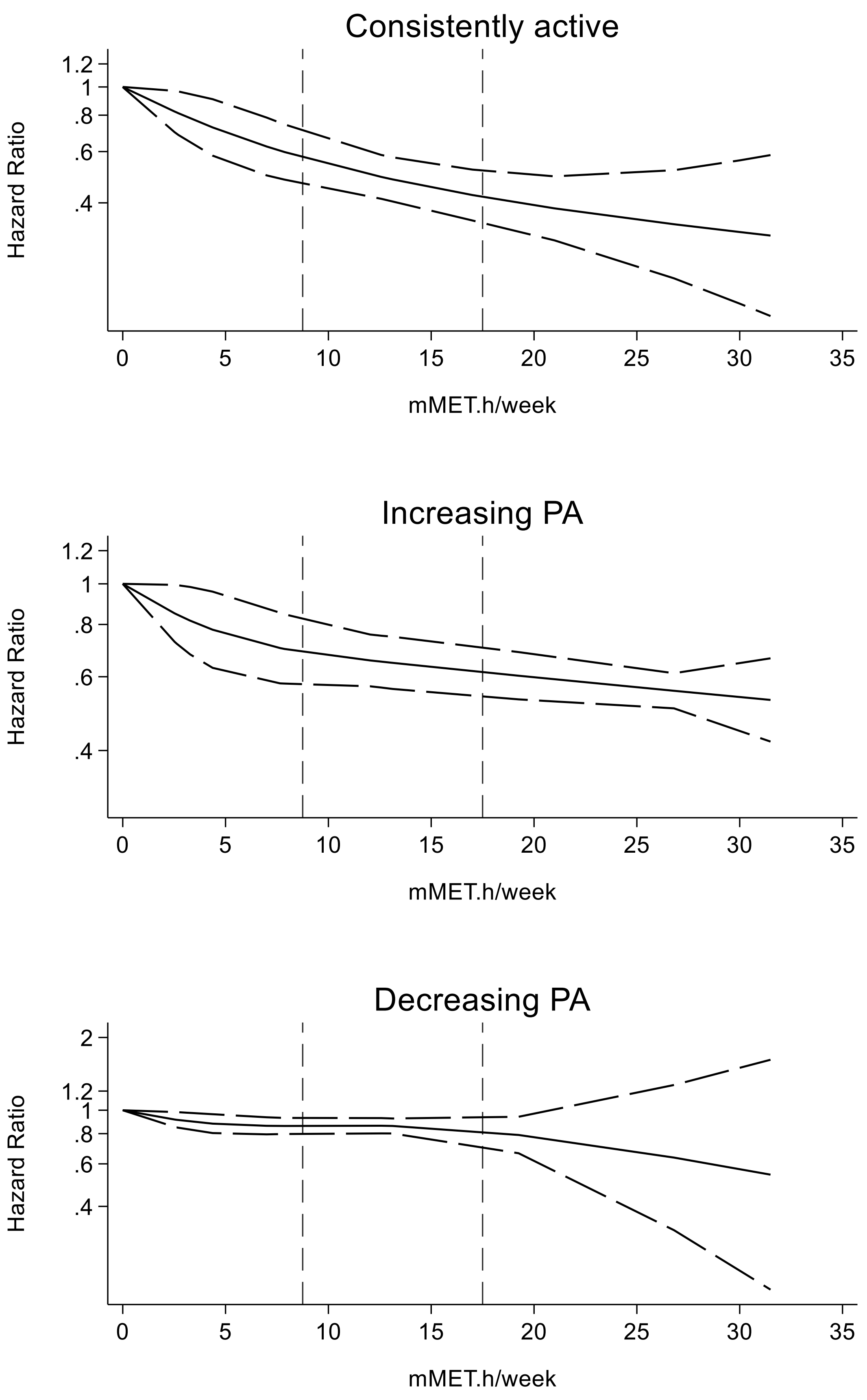

Supplementary Figure 9. Dose-response associations between consistently active, increasing, and decreasing physical activity with cardiovascular diseases (CVD) mortality.

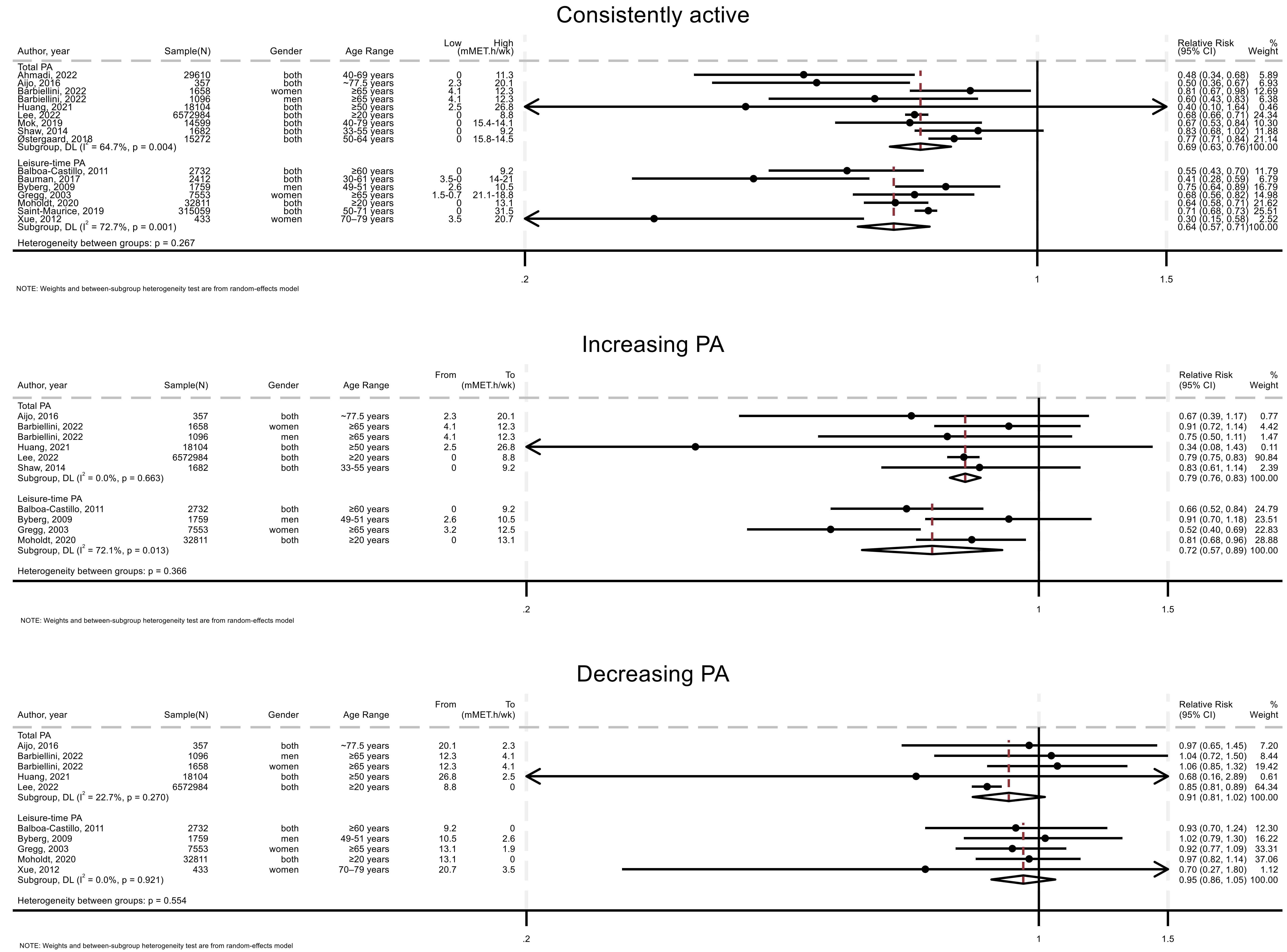

Supplementary Figure 10. Consistent, increasing, decreasing PA patterns with all-cause mortality using harmonised exposure for low (≤4.4 mMET.h/week) versus high (≥8.75 mMET.h/week).

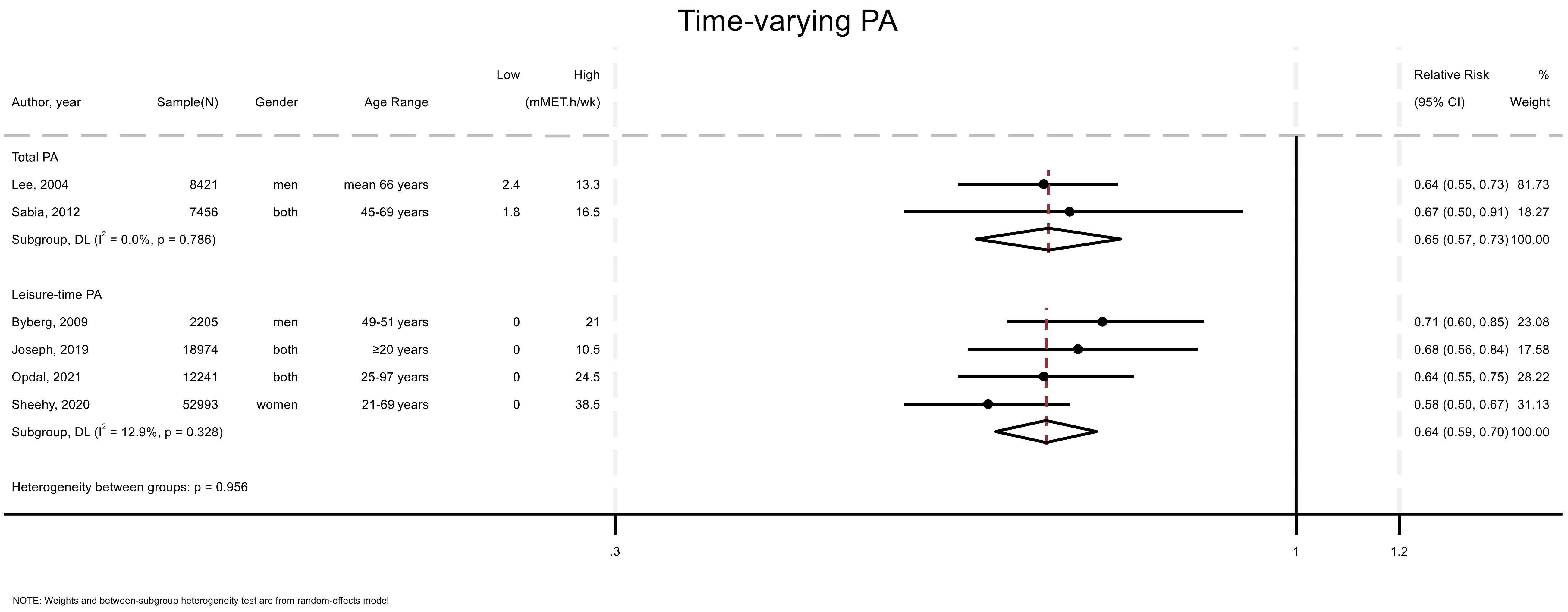

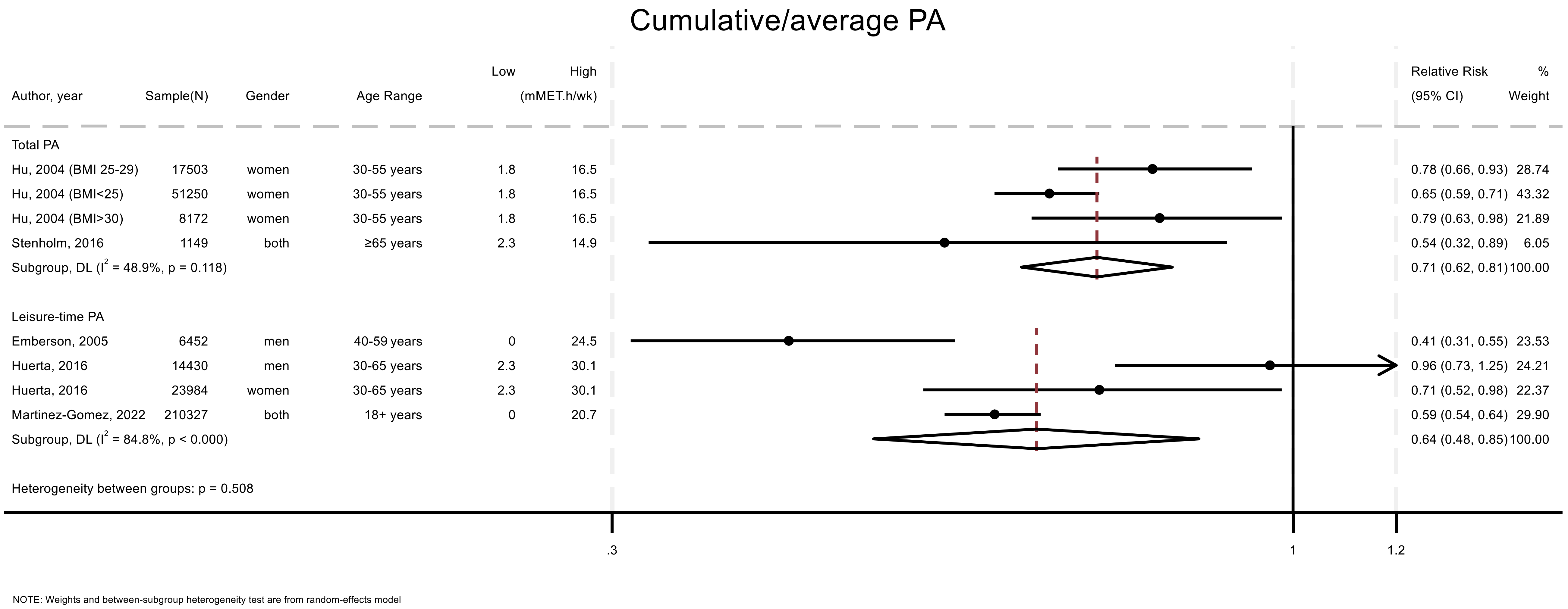

Supplementary Figure 11. Time-varying and cumulative PA with all-cause mortality using harmonised exposure for low (≤4.4 mMET.h/week) versus high (≥8.75 mMET.h/week).
